# The genome of the square archaeon *Haloquadratum walsbyi* : life at the limits of water activity

**DOI:** 10.1186/1471-2164-7-169

**Published:** 2006-07-04

**Authors:** Henk Bolhuis, Peter Palm, Andy Wende, Michaela Falb, Markus Rampp, Francisco Rodriguez-Valera, Friedhelm Pfeiffer, Dieter Oesterhelt

**Affiliations:** 1 Department of Microbial Ecology, Centre for Ecological and Evolutionary Studies, University of Groningen, The Netherlands; 2 Department of Membrane Biochemistry, Max Planck Institute of Biochemistry, Martinsried, Germany; 3 Computer Center of the Max-Planck-Society, Max Planck Institute of Plasma Physics, Garching, Germany; 4 Evolutionary Genomics Group and División de Microbiología, Universidad Miguel Hernandez, Alicante, Spain

## Abstract

**Background:**

The square halophilic archaeon *Haloquadratum walsbyi *dominates NaCl-saturated and MgCl_2 _enriched aquatic ecosystems, which imposes a serious desiccation stress, caused by the extremely low water activity. The genome sequence was analyzed and physiological and physical experiments were carried out in order to reveal how *H. walsbyi *has specialized into its narrow and hostile ecological niche and found ways to cope with the desiccation stress.

**Results:**

A rich repertoire of proteins involved in phosphate metabolism, phototrophic growth and extracellular protective polymers, including the largest archaeal protein (9159 amino acids), a homolog to eukaryotic mucins, are amongst the most outstanding features. A relatively low GC content (47.9%), 15–20% less than in other halophilic archaea, and one of the lowest coding densities (76.5%) known for prokaryotes might be an indication for the specialization in its unique environment

**Conclusion:**

Although no direct genetic indication was found that can explain how this peculiar organism retains its square shape, the genome revealed several unique adaptive traits that allow this organism to thrive in its specific and extreme niche.

## Background

Halophilic archaea (hereafter haloarchaea) predominate in NaCl-saturated aquatic ecosystems in which the salinity increases up to about ten-times the average seawater concentration. Further concentration of thalassic (seawater derived) hypersaline environments leads to the precipitation of magnesium salts thereby forming the absolute limit for life, since magnesium saturated waters (bitterns) are devoid of active life [1]. And yet, up to this catastrophic event, haloarchaea are plentiful and reach population densities that rival the most productive natural aquatic environments known on Earth. Out of the more than 15 genera of haloarchaea, only one is responsible for the booming population explosion that follows the precipitation of NaCl. Square, non-motile, pigmented archaea dominate in most thalassic NaCl-saturated environments, reaching population densities of over 10^7 ^cells per ml. The two unique features of these cells are the wafer like rectangular shape and a cell thickness of not more than 0.1 μm. Already known since the early 1980s as Walsby's square bacterium [2], the organism resisted attempts to isolation for the next 25 years. However, in 2004, two strains of the square archaeon were independently isolated from a Spanish [3] and an Australian solar saltern [4]. In their specific habitat these squares are challenged by the sub-lethal conditions of an extremely high MgCl_2 _concentration and high solar irradiance. The hygroscopic properties of the divalent Mg^2+ ^ions dramatically decrease the water activity (A_w_), a measure for the availability of free water molecules for biological processes [5,6]. The A_w _is 1.0 for pure water, 0.75 for a saturated NaCl solution and 0.3 for a saturated MgCl_2_ solution [7]. The actual A_w _of the MgCl_2 _enriched brines is unknown, but will decrease upon further concentration. Currently an Aw of 0.6 is recognized as the lower limit for life [6]. This means that although the organism thrives in an aqueous environment it suffers severe desiccation stress. Special mechanisms are therefore required to maintain optimal water activity within the cell and at the cell surface. Concomitant with the extremely high salinity, the amount of dissolved oxygen decreases to near anoxia and some essential nutrients (e.g. phosphates) become unavailable due to complexation with Mg^2+^. Here, we present features from the genome of *Haloquadratum walsbyi *that might explain the worldwide success of this organism in saturated brines.

## Results and discussion

### *H. walsbyi *expresses a water enriched capsule

**Figure 1:**
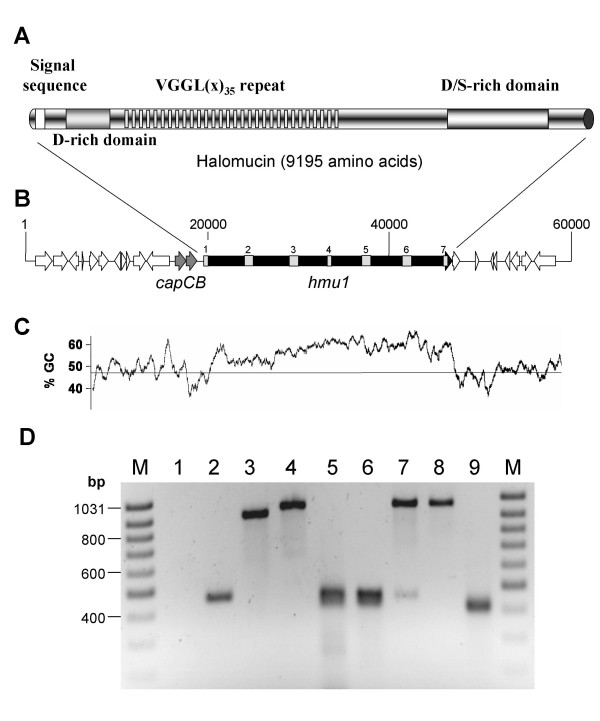
***H. walsbyi *encodes halomucin, an extremely large protein homologous to mammalian mucins**. GC content (C) and gene organization (B) of a 60.000 bp long region of the genome of *H. walsbyi*. This region contains the large, high GC gene encoding halomucin (*hmu1*) and the two capsular protein biosynthesis genes *capC *and *capB*. The domain organization of halomucin (A) reveals domains typically found in mammalian mucins. The letters D, S, V, G, and L indicate domains enriched for the corresponding amino acids. Halomucin expression is shown by detection of its mRNA via RT-PCR (D). cDNA was prepared by reverse transcription using primer sets numbered 1 to 7 the position of which correspond to the striped and numbered boxes in the *hmu1 *gene (B).

The secret to the success of *H. walsbyi *in low water activity environments might rely in a remarkable convergence with metazoans and more specifically with mammals. This is provided by the most eye-catching gene in its genome, an over 27.000 nucleotides long gene encoding a 9.159 amino acid long protein, the largest archaeal protein known to date (Fig. 1). This giant protein, termed halomucin (Hmu1) is similar in amino acid sequence and domain organization (Fig. 1A) to animal mucins, which play an important role in protecting various tissues against desiccation (e.g. in bronchial epithelium and eyes) or harsh chemical conditions (e.g. in epithelia along the digestive tract) [8]. In function, mediating a specific adaptation to desiccation stress, halomucin resembles the mucous cocoon of lungfish that can escape dehydration for several years outside the water. The presence of an N-terminal signal sequence suggests that this extremely large protein is translocated across the membrane, in accordance with a function outside the cell. Similar to animal mucins, halomucin contains domains that act as possible sites of glycosylation and sulfation which may further contribute to its overall negative charge, thereby creating an aqueous shield covering the cells. By means of Reverse Transcriptase PCR analysis, using primers specifically binding to various parts of the *hmu1 *mRNA, we could show that the gene is transcribed in full length (Fig. 1D). In addition, *H. walsbyi *encodes two 'smaller' proteins analogues to halomucin, termed Hmu2 (2885 aa) and Hmu3 (2079 aa), and at least 14 different S-layer glycoprotein homologs. Animal mucins may contain sialic acids which form rigid structures, capping the end of polysaccharide side chains of mucins. Although widespread in eukaryotes and some prokaryotes, sialic acids were not yet detected in archaea. *H. walsbyi *is probably able to synthesize sialic acid as it contains the two essential sialic acid biosynthesis genes *neuA *and *neuB. Methanococcus jannaschii *contains a gene encoding NeuB but lacks NeuA [9]. In addition to sialic acids, *H. walsbyi *can potentially also synthesize a poly-gamma-glutamate capsule by means of the bacterial-type poly-gamma-glutamate biosynthesis protein complex CapBCA [10]. Besides protection against desiccation, a cross-linked matrix of poly-gamma-glutamate may also contribute to the rigidity and maintenance of the unique square cell morphology of *H. walsbyi*.

### *H. walsbyi *encodes two bacteriorhodopsin proteins

A consequence of the extremely high salinity is the decreased solubility of oxygen (about 20% of the amount of oxygen dissolved in freshwater). Low diffusion rates, relatively high temperatures, high oxygen consumption rates, and limited oxygenic photosynthesis leave the NaCl-saturated brines virtually anoxic. Moreover, complexation of essential nutrients with the excessive amounts of cations imposes an additional problem in acquiring sufficient sources of energy, nutrients and trace elements. Oligotrophic microorganisms are well adapted to nutrient limitation, e.g. by increasing the surface to volume ratio thereby optimizing the nutrient uptake capacity relative to cell volume. Most oligotrophes achieve a high surface to volume ratio (s/v) by reducing their cell diameter; *H. walsbyi *does so by extremely flattening itself [11]. This strategy gives it what is probably the highest s/v ratio within the microbial world. Whereas spherical shaped microorganisms have to remain small in order to retain an optimal s/v ratio, the squares can become unlimitedly large since the s/v ratio solely depends on their thickness which in nature always appears to be very low (0.1 – 0.5 μm). In liquid cultures of *H. walsbyi *large cells of 40–40 μm and larger have been observed [3]. In analogy to the oligotrophes, the high s/v ratio hints to a lifestyle in which membrane processes are of major importance.

**Figure 2:**
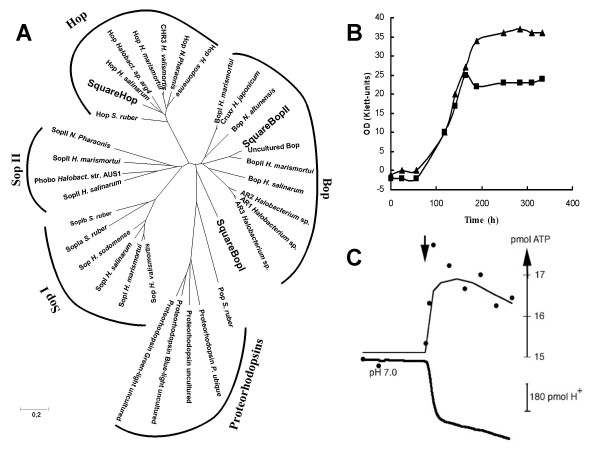
**Phototrophic growth in *H. walsbyi***. (A) Protein phylogenetic tree showing the position of the "opsin"-homologs encoded by *H. walsbyi*, indicated as SquareBopI & II and SquareHop for the proton pumping bacteriorhodopsins and chloride pumping halorhodopsin respectively. Trees were constructed with the neighbor-joining method using 1000 bootstrap replicates. Bop, bacteriorhodopsins; Hop, halorhodopsins; SopI & II; sensory-rhodopsins. (B) Phototrophic growth of aerobic (▲) and anaerobic (■) cultures of *H. walsbyi *incubated in the light. (C) Photo-phosphorylation in *H. walsbyi *measured through light induced ATP generation (upper curve) and proton (H^+^) extrusion (lower curve). Phylogenetic analysis was performed using the Microbial Genome Analysis System package MiGenAS [38,39].

**Figure 3:**
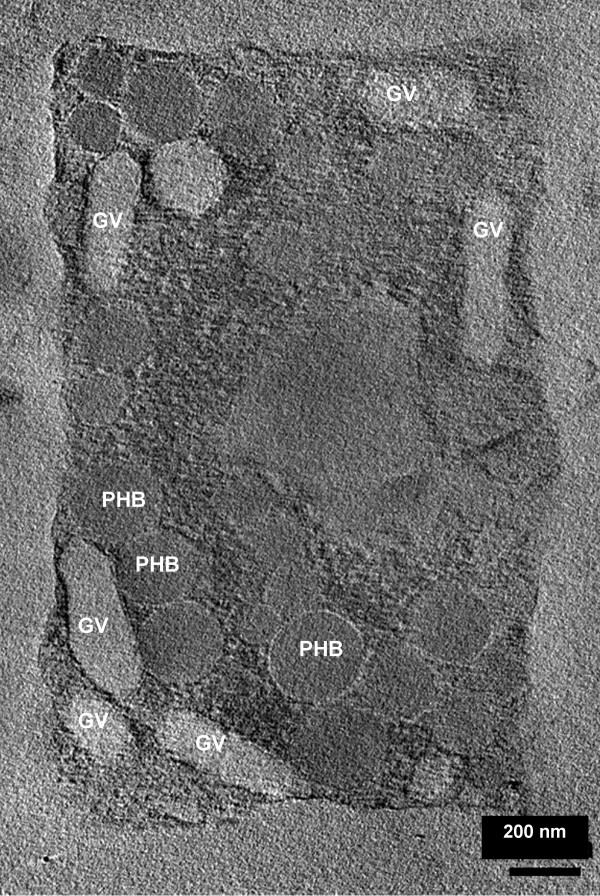
**Electron Tomographic image of a single square cell of *H, walsbyi***. In agreement with previous observations, gas vesicles (GV) recognized by their spindle shape are found at the borders of the cell. The large number of circular electron-dense bodies are most likely poly-3-hydroxy-butyric acid (PHB) polymers consistent with Nile-Blue staining patterns [4]. The genes encoding PHB biosynthesis proteins have been identified (image by H. Engelhardt).

Indeed, in addition to a set of normal haloarchaeal transporters (see gene-list in the supplementary material), *H. walsbyi *contains several unique ABC-type and major facilitator superfamily transporters that are not found in any of the other haloarchaea or in archaea in general. Amongst these are a TRAP-type C4-dicarboxylate transport system, 2 different ABC-type sulfonate transport systems and a phosphonate transport system (see below). An important process that is stimulated by the large membrane surface is the ability of *H. walsbyi *to grow phototrophically as shown in Figure 2. Only at a high s/v ratio the transmembrane protein bacteriorhodopsin, the responsible protein for phototrophic growth, can increase drastically in copy-number without taking away membrane space from other membrane components especially transport proteins and without affecting cell viability. Similar to solar panels, the ultra thin cells of *H. walsbyi *collect light as alternative energy source making optimally use of both sides of the membrane. The abundantly present gas vesicles, which are mainly located close to the cell periphery (Fig. 3), aid the cells to position themselves close and parallel to the surface. The well conserved haloarchaeal gas vesicle biosynthesis proteins are encoded by 12 genes located on two separate gene clusters *gvpACNO *and *gvpFGHIJKLM*. These clusters are separated by a 3.6 kb region containing several non-functional IS-element fragments and a ParA domain protein.

**Figure 4:**
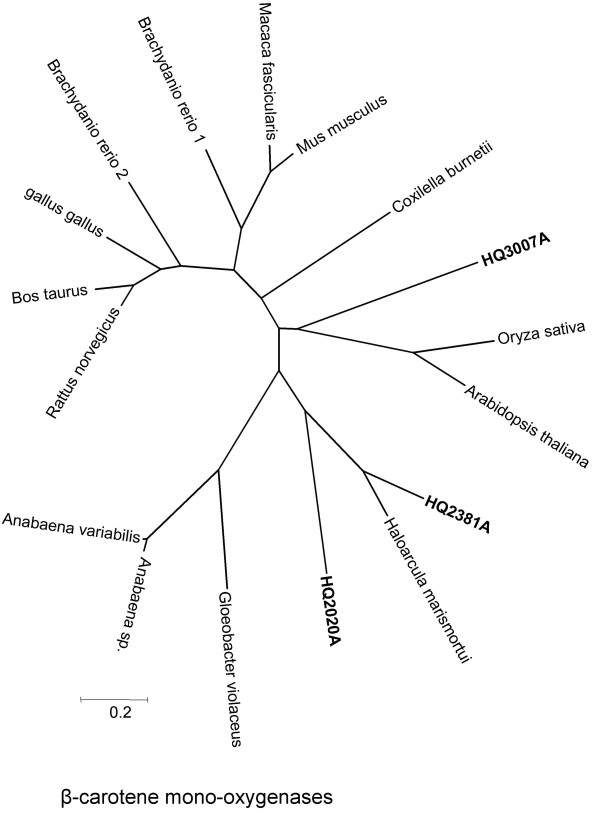
**Phylogenetic tree showing the position of the three β-carotene 15,15' mono-oxygenase homologs HQ2020A, HQ2381A, HQ3007A of *H. walsbyi***. Trees were constructed with the neighbor-joining method using 1000 bootstrap replicates.

Three photoactive proteins are present, two proton pumping bacteriorhodopsins and one chloride pumping halorhodopsin. As expected, this non-motile organism lacks phototactic sensory rhodopsins as well as any flagellar or chemotactic protein. Both proton pumping rhodopsins, BopI and BopII, and the chloride pumping halorhodopsin contain all conserved amino acids essential for retinal binding and ion translocation. Phylogenetic analysis shows that BopI is positioned close to the root of the phylogenetic tree and might form a missing link to the eubacterial proteorhodopsins (Fig. 2). The genes encoding BopI and BopII are located very near to each other and close to the origin of replication, a fact that strengthens their importance for the biology of this organism. Interestingly, preliminary analysis of membrane protein expression patterns shows that BopI is amongst the most abundantly expressed membrane proteins (Fusetti and Poolman; unpublished results). Essential in the formation of functional rhodopsins is the synthesis of its chromophore retinal. Surprisingly, *H. walsbyi *encodes not less than three β-carotene mono-oxygenase homologs involved in the cleavage of beta-carotene yielding retinal [12], two of which are closely related to cyanobacterial β-carotene mono-oxygenases whereas the third is more similar to homologs in plants (Fig. 4). The recently sequenced genome of *Haloarcula marismortui *encodes a single β-carotene mono-oxygenase homolog on one of its plasmids [13], whereas no homologs of the *H. walsbyi *enzymes were found in the other phototrophic haloarchaea for which the protein responsible for β-carotene cleavage still remains to be identified.

### Phosphate metabolism in *H. walsbyi*

**Figure 5:**
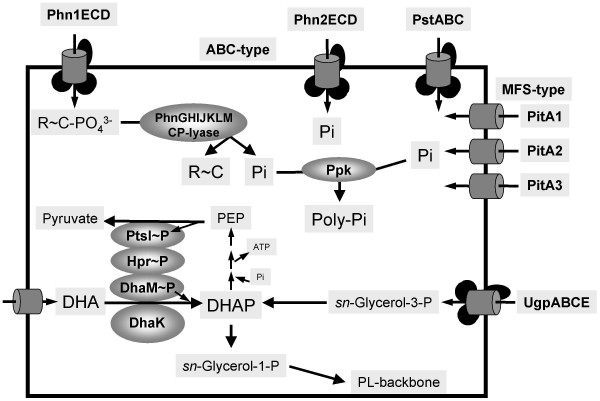
**Phosphate metabolism in *H. walsbyi***. Scheme revealing the proteins involved in phosphate metabolism. The transporters PitA1-3, Phn2ECD, PstABC and phosphate storage enzyme polyphosphate kinase (Ppk) are common in several haloarchaea. Unique to *H. walsbyi *are the bacterial-type proteins involved in phosphonate metabolism, an ABC-type phosphonate uptake system plus phosphonate lyase, and the PTS dependent dihydroxyacetone kinase (DHAK). DHA, dihydroxyacetone; DHAP, dihydroxyacetone phosphate; Pi, phosphate ion; PEP, phospho *enol*pyruvate; PL, phospholipid.

In the presence of a high Mg^2+ ^concentration especially phosphates tend to form insoluble complexes and may become limiting for growth. Indeed, growth under phosphate limitation is reflected in a large number of exclusive traits found in the genome of *H. walsbyi *(Fig. 5). Unique amongst the archaea is the presence of a gene cluster that allows uptake of phosphonates and subsequent cleavage of the stable carbon-phosphorus bond by a phosphonate lyase. In various bacteria, phosphonate metabolism is known to be induced under phosphate limitation [14,15]. Moreover, phosphonates are ubiquitous in nature in the form of phosphonolipids and as side groups of polysaccharides and glycoproteins [15]. *H. walsbyi *also contains an operon encoding UDP-sulfoquinovose synthase (SqdB) plus two UDP-glucose 4-epimerase (GalE) homologs, key-enzymes in sulfolipid biosynthesis. Sulfolipids have been found to replace phospholipids in response to phosphate limitation in a number of photosynthetic bacteria and plants [16-18].

### *H. walsbyi *utilizes dihydroxyacetone via a phosphoenolpyruvate dependent phosphotransferase system

Exceptional among archaea is the presence of a phospho*enol*pyruvate (PEP) dependent phosphotransferase (PTS) system involved in the phosphorylation of dihydroxyacetone (DHA). PEP-PTS systems were so far only found in bacteria in which phosphorylation of substrates is coupled to their translocation over the membrane [19]. In many bacteria DHA is phosphorylated by an ATP dependent dihydroxyacetone kinase (DhaK). However, some bacteria and *H. walsbyi *contain a unique cytosolic PEP-PTS dependent DhaK in which DHA is phosphorylated on the expense of PEP rather than ATP to give dihydroxyacetone-phosphate (DHAP) [20]. DHA is translocated over the membrane via facilitated diffusion, a process that is driven by its concentration gradient. Maintenance of an inwardly-directed DHA gradient is achieved by phosphorylation of DHA by the PTS system in the cytosol rather than by a membrane associated PTS system. DHAP can be used as substrate for gluconeogenesis or glycolysis. In the glycolytic reaction DHAP is converted back to PEP resulting in the net generation of one molecule of pyruvate and one molecule of ATP for each molecule of DHA taken up (Fig. 5). Recent experimentation showed that *H. walsbyi *can grow on DHA as carbon and energy source (data not shown). Alternatively, DHAP is also an important intermediate in the formation of the stereoisomer *sn*-glycerol-1-phosphate which is part of the archaea-specific backbone of membrane lipids [21]. Interestingly, dihydroxyacetone is a putative overflow product of glycerol metabolism in *Salinibacter ruber*, the dominant bacterium in crystallizer ponds [22]. Metabolism of dihydroxyacetone by *H. walsbyi *might explain the observed synergistic effect on *H. walsbyi *colony formation when grown in association with *S. ruber *[3]. In addition to DHA, *H. walsbyi *can grow on glycerol and pyruvate [3] but also on amino acids [4] for which all biosynthesis pathways are completely present. Glycerol and pyruvate are probably taken up by diffusion since specific uptake systems have not been identified. For the amino acids a large repertoire of amino acid uptake systems are present.

### General genome properties

**Figure 6:**
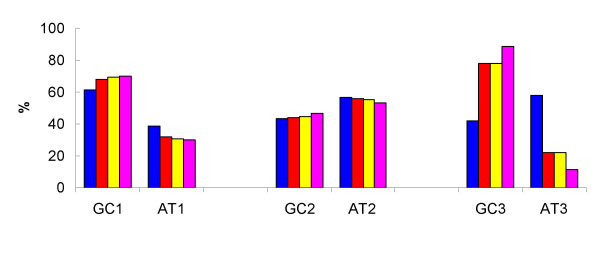
**Codon usage in halophilic archaea**. Indicated is the percentage of G plus C or A plus T at the different codon positions showing the large difference between *H. walsbyi *and the other haloarchaea especially at the third codon position. Bars are *H. walsbyi *(blue), *H. marismortui *(red), *N. pharaonis *(yellow) and *H. salinarum *(purple).

The Genome of *H. walsbyi *strain HBSQ001, DSM 16790, comprises a 3,132,494 bp chromosome and a 46,867 bp plasmid and contains 2738 and 39 open reading frames (ORFS) respectively (Table 1). See [additional file 1] for a full list of proteins encoded by *H. walsbyi*. The base pair numbering of the genome was started approximately 50 nucleotides before the well conserved haloarchaeal region containing the origin of replication and the cell division control protein CDC6_1 (HQ1001A) [23]. A striking feature of *H. walsbyi's *genome is a remarkably low GC content of 47.9% in contrast to the generally high GC content (60–70% GC) of other haloarchaea. The GC content is constant over the genome with the exception of some GC-rich islands that are highly enriched in transposases and phage related genes. The GC content of these transposases is similar to the GC content of transposases found in other haloarchaea (54 %) suggesting that they share a common pool of transposable elements and phages. The low GC content of the genome is intriguing since it is generally assumed that a high GC content is a specific adaptation of haloarchaea to the high levels of UV irradiation in the shallow coastal lagoons they inhabit. AT-rich genomes are more prone to UV induced thymidine dimer formation and possible accumulation of mutations than GC-rich genomes. In *H. walsbyi *this might partly be compensated by the large number of photolyases (4 copies). Despite the exceptional GC content, the majority of the typical haloarchaeal proteins encoded by *H. walsbyi *are highly conserved at the amino acid sequence level. Also the characteristic overall high acidity of haloarchaeal proteins is well conserved in *H. walsbyi *(average pI = 5.1). As expected, *H. walsbyi *differs from the other haloarchaea mainly at the third codon position (Fig. 6). Conservation of amino acid sequence and restriction of GC deviation to the third codon position favor the hypothesis that *H. walsbyi *evolved from a 'normal' GC rich haloarchaeal ancestor rather than by convergent evolution from a non-related AT rich organism. A drift to an AT rich genome has been observed especially in organisms adapted to highly specialized niches such as endosymbionts [24] and the free-living marine microorganisms *Prochlorococcus *sp. [25] and *Pelagibacter ubique *[26]. For two closely related species of *Prochlorococcus *that differ over 20% in overall GC content (30.8 % GC and 50.7% GC) but less than 3% at the 16S rDNA level it was argued that an enrichment in AT is a side effect of decreased selective pressure in a physically limited environment in which the low GC strains thrive. Mutational bias would increase the AT content by neutral drift. A lower GC content in the bacterial strains may be of advantage due to the slightly decreased demand for nitrogen, since guanine requires the incorporation of one additional nitrogen atom as compared to the other nucleotides [25,26].

Similar to these microorganisms, *H. walsbyi *occupies a relatively stable but narrow ecological niche. However, nitrogen does not appear to be limiting in its natural habitat, and so we hypothesize that another factor, namely adaptation to the extremely high MgCl_2 _concentration, is responsible for the drift to an AT rich genome in *H. walsbyi*. Despite the presence of energy demanding cation efflux systems, the high external magnesium concentration will lead to an increase in the internal magnesium concentration that is higher than in other microorganisms. Magnesium ions are known to have a stabilizing effect on the DNA duplex, the secondary structure of RNA (Carter and Holbrook) and DNA-RNA heteroduplexes. In case of an already stable high-GC genome the additional stabilizing effect of magnesium might result in DNA rigidity that interferes with essential processes like DNA replication and transcription. We propose that the drift to an AT-rich genome might be induced as a long term evolutionary adaptation to this over-stabilization by magnesium and can be balanced by lowering the GC content of the genome.

### The genome of *H. walsbyi *has a low coding density

A related peculiarity of the *H. walsbyi *genome is its remarkably low coding density (76%) as compared to other haloarchaea (86–91%) and prokaryotes in general [27]. This is due to a very large average intergenic spacing of 289 bp mainly because of a high number of very long (> 1000 bp) intergenic regions. These long intergenic regions consist of non-coding DNA fragments, novel DNA repeat elements and pseudogenes, in most cases remnants of IS transposases. The low coding density, high number of pseudogenes and IS elements, and the drift towards a more AT rich genome may be signs that *H. walsbyi *is in a stage where it is undergoing genome shrinkage possibly due to its specialization into a very restrictive and specific environment with subsequent lack of growth competition from other species. Although saturated brines are present around the world and already exist since ancient geologic periods, competition with other microbes will be very relaxed in these physically limited environments, in a way similar to what happens with intracellular parasites or endosymbionts. The regular desiccation of these evaporative systems might act as evolutionary bottlenecks also favoring genome degradation [3].

### Description of the plasmid

The 47 kb plasmid PL47 has a homogeneous GC distribution, is similar in GC content to the chromosome (Table 1) and contains thirty-nine open reading frames. Most genes are hypothetical or conserved hypothetical. Of the identified genes, the majority encode proteins involved in plasmid maintenance, replication and restriction modification with the majority being of bacterial or viral (phage) descent rather than of archaeal descent. Probably these proteins are dedicated to the replication and maintenance of the plasmid itself. However, the plasmid replication protein RepH is not encoded on the plasmid but is located on the main chromosome. In addition, the plasmid does not contain a homolog of the CDC6 cell division control protein that is commonly found on the smaller replicons of other haloarchaea. The gene coverage (69%) of PL47 is even lower than that of the chromosome with an average gene distance of 371 bp.

## Conclusion

In addition to its eye-catching shape, the square archaeon *H. walsbyi *is in many ways unique amongst haloarchaea. Its genome revealed a broad range of novel adaptive traits in both genome composition and protein sequences that may have contributed to this organism's domination in saturated brines. Further functional studies are required to test these assumptions. Finally, these findings provide clues about how life is possible in the 5 M MgCl_2 _containing Discovery basin in the Mediterranean deep sea that was recently shown to contain a unique microbial community [28] and possibly even in the proposed brines at the surface of Jupiter's moons Europa and Ganymede.

## Methods

### Cultivation, genome sequencing and assembly

The Spanish isolate of the square halophilic archaeon *Haloquadratum walsbyi *strain HBSQ001 (DSM 16790) was grown to end exponential phase as described before [3]. *H. walsbyi *was sequenced with 6.5-fold sequence coverage using a shotgun clone library (average insert size of 3 kb), and assembled with the PHRED-PHRAP-CONSED package [29]. The sequence is of high quality (0.01 Errors/10 kb).

### Gene prediction and annotation

For gene prediction, REGANOR [30] from the annotation package GENDB [31] was used, which integrates results from CRITICA [32] and GLIMMER [33]. The automatically predicted ORF set (3013 ORFs) was expert-curated resulting in a theoretical proteome of 2777 proteins. Curation involved sequence comparison to proteins from other halophiles (*Halobacterium salinarum *strain R1, [34]), *Natronomonas pharaonis *[35], *Haloarcula marismortui *[13] and public protein sequence databases. This permitted to identify additional small proteins and to improve the correctness of start codon assignments. tRNAs and other RNAs were predicted using tRNAscan [36] and BLAST [37] against other halophiles, respectively. Phylogenetic analysis of proteins was performed using the Microbial Genome Analysis System package MiGenAS [38,39] and the MEGA3 phylogenetic tool software package [40,41].

### General genome properties

The genome can be accessed via HaloLex [34]. General features and statistics on the genome of H. walsbyi are shown in Table S1. The main origin of replication is located in a highly conserved region and consists of a conserved stem-loop structure, and open reading frames encoding the conserved CDC6 cell division control protein, a signal sequence peptidase and DNA polymerase B [42]. The sequence has been submitted to EMBL under the accession numbers [EMBL:AM180088, EMBL:AM180089] for the genome and plasmid PL47 respectively.

### Extraction of total RNA and DNase I digestion

The RNA was extracted with peqGold RNAPure extraction solution (Peqlab Biotechnology) following the manufacturers instructions. After dissolving RNA in DEPC-H_2_O residual DNA was digested using the "DNA-free" kit (Ambion) following the manufacturers instructions. The quality of the RNA was checked using the 2100 Bioanalyzer (Agilent) and the RNA Nano LabChip (Agilent).

### cDNA synthesis

Total RNA was reverse transcribed into cDNA using SuperScript II (Invitrogen) following the manufacturer's instructions with 2 μg total RNA per reaction as template and the gene specific primers pcr4-rev and pcr7-rev, respectively.

### PCR reactions

The PCR reactions were performed using HotStarTaq (Qiagen) (50 μl per reaction) and 0.5 μl of the cDNA samples as template. The following temperature profile was applied on a Thermocycler T3 (Biometra): 95°C 15 min; 40× (95°C 30 sec, 60°C 30 sec, 72°C 50 sec (500 bp-PCRs) or 90 sec (1 kbp-PCRs). Subsequently the PCR reactions were analyzed by standard agarose gel electrophoresis.

### Primer sequences

pcr1-for: 5'-CAT TGG ATC GGT GTC TGC ACA GCA AC-3'

pcr1-rev: 5'-GCG CCG CTT GAA GGA GTT ATT TGC G-3'

pcr2-for: 5'-GAT CAC GCT CGA CGA CCT CG-3'

pcr2-rev: 5'-CGT TGA TGA CGC CAG CCT GC-3'

pcr3-for: 5'-CCA CTG GTC AGG TGA ATG CCT C-3'

pcr3-rev: 5'-CTT CCT GTC GCA TCC GAC TGG-3'

pcr4-for: 5'-GAC GCT ACT GCC ACC GGC GAT G-3'

pcr4-rev: 5'-GCA GAC CCG TGT TCG AAC CGT CC-3'

pcr5-for: 5'-GGA CTT GCT GGC ACG ATC GAC-3'

pcr5-rev: 5'-CTC CAG ATG TGC CAA CCT CGC-3'

pcr6-for: 5'-GCG GTT GAG TGG TAT CTT CAC C-3'

pcr6-rev: 5'-GCT ATC GGT GGC GGT GTC G-3'

pcr7-for: 5'-CTC CCC ATC CAG TAG TCG GTC ATT GG-3'

pcr7-rev: 5'-GAT TGT ATC CTC TCA AAT GCC CCG CTA AG-3'

## Authors' contributions

Shotgun library preparation and sequencing was performed by PP. MF and FP provided genome assembly, initial ORF prediction and bioinformatics tools integrated into the HaloLex system and performed the genome statistics. AW performed the mRNA detection study of Halomucin. MR programmed and supported the genome analysis system MIGENAS and supported genome annotation. HB supervised the study, performed the manual annotation and wrote the manuscript jointly with FRV, FP and DO.

## Supplementary Material

Additional file 1 complete list of *H. walsbyi *proteins. List of all *Haloquadratum *proteins ordered by function category and function class. Click here for file

## Figures and Tables

**Table 1:** General features of the *H. walsbyi *genome

Feature	Chromosome	plasmid PL47
Replicon length (bp)	3,132,494 bp	46,867 bp
G+C content (%)	47.9 %	47.7 %
G+C of transposases (%)	52 %	-
Number of protein-coding genes	2738	39
Average size of proteins	290 aa	276 aa
Percentage coding for proteins or RNAs	76.5 %	69.1 %
Average gene distance	289	371
Number of rRNA operons (16S, 23S, 5S)	2	-
Number of tRNAs	43	-
Number of other RNAs (7S, RNAseP)	2	-
Average pI of proteins	5.1	5.2
