# Supplementary material for: The genome of the square archaeon Haloquadratum walsbyi : life at the limits of water activity
Source: BMC Genomics. 2006 Jul 4;7:169. doi: 10.1186/1471-2164-7-169 (PMC1544339; doi:10.1186/1471-2164-7-169)
Supplement: Additional file 1 — complete list of H. walsbyi proteins. List of all Haloquadratum proteins ordered by function category and function class. [file 1471-2164-7-169-S1.doc]

**Table S1: List of all *Haloquadratum* proteins ordered by function category and function class**

For each protein, its systematic name, gene name, and protein name is given. The table is ordered by function category (dark grey) and by function class (light grey) within each category. An updated version of this table will be maintained at HaloLex (www.halolex.mpg.de).

| **MET** | |  | | **metabolism** |
| --- | --- | --- | --- | --- |
| **EM** | |  | | **energy metabolism** |
| *code* | | *gene* | | *protein name* |
| HQ1014A | | *bopI* | | bacteriorhodopsin precursor [Squarebop I] |
| HQ1016A | | *ferC* | | ferredoxin III |
| HQ1017A | | *bopII* | | bacteriorhodopsin precursor [Squarebop II] |
| HQ1076A | | *fer* | | ferredoxin (2Fe-2S) |
| HQ1128A | | *hoxF* | | NAD-reducing hydrogenase, alpha subunit (EC 1.12.1.2) |
| HQ1129A | | *fdhAI* | | NADH:ubiquinone oxidoreductase / formate dehydrogenase alpha subunit I (EC 1.2.1.2) |
| HQ1130A | | *fdhAII* | | NADH:ubiquinone oxidoreductase / formate dehydrogenase alpha subunit II (EC 1.2.1.2) |
| HQ1132A | | *fdhA* | | NADH:ubiquinone oxidoreductase / formate dehydrogenase, alpha subunit (EC 1.2.1.2) |
| HQ1299A | | *-* | | predicted F420-dependent NADP reductase (EC 1.-.-.-) |
| HQ1323A | | *trxA* | | thioredoxin |
| HQ1526A | | *ndh* | | NADH dehydrogenase (EC 1.6.99.3) |
| HQ1548A | | *hcpE* | | halocyanin hcpE |
| HQ1550A | | *petB* | | menaquinol--cytochrome-c reductase (cytochrome bc complex) cytochrome b subunit (EC 1.10.2.-) |
| HQ1551A | | *petD* | | menaquinol--cytochrome-c reductase (cytochrome bc complex) cytochrome b/c subunit (EC 1.10.2.-) |
| HQ1557A | | *hcpF* | | halocyanin hcpF |
| HQ1578A | | *coxB* | | cytochrome-c-like terminal oxidase, subunit II (EC 1.9.3.1) |
| HQ1611A | | *coxA* | | cytochrome-c-like terminal oxidase, subunit I (EC 1.9.3.1) |
| HQ1614A | | *trxA* | | thioredoxin |
| HQ1629A | | *trxA* | | thioredoxin |
| HQ1637A | | *nuoA* | | NADH dehydrogenase, subunit A (ubiquinone) (EC 1.6.5.3) |
| HQ1638A | | *nuoB* | | NADH dehydrogenase, subunit B (ubiquinone) (EC 1.6.5.3) |
| HQ1639A | | *nuoCD* | | NADH dehydrogenase, subunit CD (ubiquinone) (EC 1.6.5.3) |
| HQ1640A | | *nuoH* | | NADH dehydrogenase, subunit H (ubiquinone) (EC 1.6.5.3) |
| HQ1641A | | *nuoI* | | NADH dehydrogenase-like complex, subunit I |
| HQ1643A | | *nuoJ* | | NADH dehydrogenase, subunit J (ubiquinone) (EC 1.6.5.3) |
| HQ1644A | | *nuoJ2* | | NADH dehydrogenase-like complex, subunit J2 |
| HQ1645A | | *nuoK* | | NADH dehydrogenase, subunit K (ubiquinone) (EC 1.6.5.3) |
| HQ1646A | | *nuoL* | | NADH dehydrogenase, subunit L (ubiquinone) (EC 1.6.5.3) |
| HQ1647A | | *nuoM* | | NADH dehydrogenase, subunit M (ubiquinone) (EC 1.6.5.3) |
| HQ1648A | | *nuoN* | | NADH dehydrogenase, subunit N (ubiquinone) (EC 1.6.5.3) |
| HQ1942A | | *coxM* | | aerobic-type carbon monoxide dehydrogenase, medium subunit (EC 1.2.99.2) |
| HQ1943A | | *coxS* | | aerobic-type carbon monoxide dehydrogenase, small subunit (EC 1.2.99.2) |
| HQ1944A | | *coxL* | | aerobic-type carbon monoxide dehydrogenase, large subunit (EC 1.2.99.2) |
| HQ2001A | | *fdx* | | ferredoxin (2Fe-2S) |
| HQ2336A | | *ferA* | | probable ferredoxin I |
| HQ2344A | | *nqrF* | | FAD/NAD binding oxidoreductase; probable Na+-transporting NADH:ubiquinone oxidoreductase, subunit NqrF |
| HQ2352A | | *coxC* | | cytochrome-c-like terminal oxidase, subunit III (EC 1.9.3.1) |
| HQ2357A | | *hcpH* | | halocyanin hcpH |
| HQ2414A | | *fer* | | ferredoxin |
| HQ2511A | | *trxA* | | thioredoxin |
| HQ2537A | | *ferA_5* | | probable ferredoxin |
| HQ2557A | | *trxA* | | thioredoxin |
| HQ2621A | | *nuoL* | | homolog to NADH dehydrogenase, subunit L (ubiquinone) (EC 1.6.5.3) |
| HQ2648A | | *ndh* | | NADH dehydrogenase (EC 1.6.99.3) |
| HQ2910A | | *fixC* | | electron-transferring-flavoprotein dehydrogenase (EC 1.5.5.1) |
| HQ3024A | | *qor* | | NADPH:quinone reductase (EC 1.6.5.5) |
| HQ3153A | | *cofE* | | F420-0:gamma-glutamyl ligase |
| HQ3154A | | *mer* | | Coenzyme F420-dependent N(5),N(10)-methylenetetrahydromethanopterin reductase (EC 1.5.99.11) (Methylene-H(4)MPT reductase) |
| HQ3171A | | *fer1* | | dnaJ N-terminal domain / ferredoxin fusion protein |
| HQ3197A | | *pepC* | | Phosphoenolpyruvate carboxylase (EC 4.1.1.31) |
| HQ3198A | | *petA* | | probable menaquinol--cytochrome-c reductase (EC 1.10.2.-) (cytochrome bc complex) Rieske iron-sulfur protein (ubiquinol--cytochrome-c reductase (cytochrome bc1 complex) Rieske iron-sulfur protein homolog) |
| HQ3242A | | *atpD* | | H(+)-transporting two-sector ATPase, subunit D (A-type ATP synthase) (EC 3.6.3.14) |
| HQ3243A | | *atpB* | | H(+)-transporting two-sector ATPase, subunit B (A-type ATP synthase) (EC 3.6.3.14) |
| HQ3244A | | *atpA* | | H(+)-transporting two-sector ATPase, subunit A (A-type ATP synthase) (EC 3.6.3.14) |
| HQ3245A | | *atpF* | | H(+)-transporting two-sector ATPase, subunit F (A-type ATP synthase) (EC 3.6.3.14) |
| HQ3246A | | *atpC* | | H(+)-transporting two-sector ATPase, subunit C (A-type ATP synthase) (EC 3.6.3.14) |
| HQ3247A | | *atpE* | | H(+)-transporting two-sector ATPase, subunit E (A-type ATP synthase) (EC 3.6.3.14) |
| HQ3248A | | *atpK* | | H(+)-transporting two-sector ATPase, subunit K (A-type ATP synthase)(EC 3.6.3.14) |
| HQ3249A | | *atpI* | | H(+)-transporting two-sector ATPase, subunit I (A-type ATP synthase) (EC 3.6.3.14) |
| HQ3250A | | *atpH* | | H(+)-transporting two-sector ATPase, subunit H (A-type ATP synthase) (EC 3.6.3.14) |
| HQ3457A | | *fdx* | | ferredoxin (2Fe-2S) |
| HQ3667A | | *ferB* | | ferredoxin II |
| **CIM** | |  | | **central intermediary metabolism** |
| ***code*** | | ***gene*** | | ***protein name*** |
| HQ1075A | | *gnd* | | 6-phosphogluconate dehydrogenase (EC 1.1.1.44) |
| HQ1156A | | *fba* | | fructose-bisphosphate aldolase (EC 4.1.2.13) |
| HQ1276A | | *-* | | probable ribose-1,5-bisphosphate dehydrogenase |
| HQ1349A | | *citZ* | | citrate (si)-synthase (EC 2.3.3.1) |
| HQ1360A | | *gapB* | | glyceraldehyde-3-phosphate dehydrogenase (NAD(P)) (phosphorylating) (EC 1.2.1.59) |
| HQ1394A | | *gapB* | | glyceraldehyde-3-phosphate dehydrogenase (NAD(P)) (phosphorylating) (EC 1.2.1.59) |
| HQ1395A | | *pgk* | | phosphoglycerate kinase (EC 2.7.2.3) |
| HQ1455A | | *kdgK* | | 2-keto-3-deoxygluconate kinase (EC 2.7.1.45) |
| HQ1465A | | *suhB* | | probable inositol-1(or 4)-monophosphatase (EC 3.1.3.25)/ fructose-1,6-bisphosphatase (EC 3.1.3.11), archaeal type |
| HQ1474A | | *rbsK* | | Ribokinase (EC 2.7.1.15) |
| HQ1535A | | *korB* | | oxoglutarate--ferredoxin oxidoreductase (EC 1.2.7.3) beta chain |
| HQ1536A | | *korA* | | oxoglutarate--ferredoxin oxidoreductase, alpha subunit (EC 1.2.7.3) |
| HQ1560A | | *gldA* | | glycerol-1-phosphate dehydrogenase [NAD(P)] (EC 1.1.1.261) |
| HQ1570A | | *ppsA1* | | pyruvate, water dikinase (EC 2.7.9.2) (phosphoenolpyruvate synthase) |
| HQ1573A | | *pykA* | | pyruvate kinase (EC 2.7.1.40) |
| HQ1667A | | *gckA* | | probable hydroxypyruvate reductase (EC 1.1.1.81); probable glycerate kinase (EC 2.7.1.31) |
| HQ1729A | | *adh* | | probable alcohol dehydrogenase (NADP+) (EC 1.1.1.2)(EC 1.1.1.1) |
| HQ1733A | | *glpK* | | glycerol kinase (EC 2.7.1.30) |
| HQ1734A | | *gpdA* | | glycerol-3-phosphate dehydrogenase (EC 1.1.99.5) chain A |
| HQ1735A | | *gpdB* | | glycerol-3-phosphate dehydrogenase (EC 1.1.99.5) chain B |
| HQ1736A | | *gpdC* | | glycerol-3-phosphate dehydrogenase (EC 1.1.99.5) chain C |
| HQ1816A | | *-* | | probable glucose dehydrogenase, type B (EC 1.1.1.-); probable L-sorbosone dehydrogenase (EC 1.1.1.-) |
| HQ1881A | | *fbp* | | fructose-bisphosphatase (EC 3.1.3.11) |
| HQ2024A | | *ppsA2* | | pyruvate, water dikinase (EC 2.7.9.2) (phosphoenolpyruvate synthase) |
| HQ2025A | | *gapB* | | glyceraldehyde-3-phosphate dehydrogenase (NAD(P)) (phosphorylating) (EC 1.2.1.59) |
| HQ2366A | | *adh* | | alcohol dehydrogenase (EC 1.1.1.1) |
| HQ2441A | | *sdhA* | | succinate dehydrogenase (EC 1.3.99.1), subunit A (flavoprotein) |
| HQ2471A | | *fumC* | | fumarate hydratase (EC 4.2.1.2) |
| HQ2550A | | *glcD* | | probable oxidoreductase (EC 1.1.1.-) (probable glycolate oxidase iron-sulfur subunit) |
| HQ2675A | | *gpdA* | | glycerol-3-phosphate dehydrogenase, subunit A (EC 1.1.99.5) |
| HQ2736A | | *mdh* | | malate dehydrogenase (oxaloacetate decarboxylating) (EC 1.1.1.40)/ phosphate acetyltransferase (EC 2.3.1.8) |
| HQ2781A | | *gpmA* | | phosphoglycerate mutase, 2,3-biphosphateglycerate-independent type (EC 5.4.2.1) |
| HQ2795A | | *suhB* | | inositol-1(or 4)-monophosphatase (EC 3.1.3.25)/ fructose-1,6-bisphosphatase (EC 3.1.3.11), archaeal type |
| HQ2857A | | *sucD* | | succinate--CoA ligase, alpha subunit (ADP-forming) (EC 6.2.1.5) |
| HQ2858A | | *sucC* | | succinate--CoA ligase beta subunit (ADP-forming) (EC 6.2.1.5) |
| HQ2935A | | *eno* | | phosphopyruvate hydratase (EC 4.2.1.11) (enolase) |
| HQ2994A | | *sdhA* | | succinate dehydrogenase, subunit A (EC 1.3.99.1) (flavoprotein) |
| HQ2995A | | *sdhB* | | succinate dehydrogenase, subunit B (iron-sulfur protein) (EC 1.3.99.1) |
| HQ2996A | | *sdhD* | | succinate dehydrogenase, subunit D (membrane anchor protein) (EC 1.3.99.1) |
| HQ2997A | | *sdhC* | | succinate dehydrogenase, subunit C (cytochrome b-556) (EC 1.3.99.1) |
| HQ3101A | | *pgi* | | glucose-6-phosphate isomerase (EC 5.3.1.9) |
| HQ3108A | | *glcK* | | glucokinase (EC 2.7.1.2) |
| HQ3117A | | *citB* | | aconitate hydratase (EC 4.2.1.3) |
| HQ3205A | | *deoC* | | deoxyribose-phosphate aldolase (EC 4.1.2.4) |
| HQ3212A | | *icd* | | isocitrate dehydrogenase (NADP+) (EC 1.1.1.42) |
| HQ3355A | | *porB* | | pyruvate--ferredoxin oxidoreductase (EC 1.2.7.1), beta subunit |
| HQ3356A | | *porA* | | pyruvate--ferredoxin oxidoreductase (EC 1.2.7.1), alpha subunit |
| HQ3360A | | *tpiA* | | triosephosphate isomerase (EC 5.3.1.1) |
| HQ3459A | | *mdhA* | | malate dehydrogenase (EC 1.1.1.37) |
| HQ3517A | | *gpmB* | | probable fructose-2,6-bisphosphatase (EC 3.1.3.46); probable phosphoglyceromutase (EC 5.4.2.1), type 2 |
| HQ3679A | | *rpiA* | | ribose-5-phosphate epimerase (EC 5.3.1.6) |
| **AA** | |  | | **amino acid metabolism** |
| ***code*** | | ***gene*** | | ***protein name*** |
| HQ1005A | | *lysC* | | aspartate kinase (EC 2.7.2.4) |
| HQ1022A | | *hisI* | | phosphoribosyl-AMP cyclohydrolase (EC 3.5.4.19) |
| HQ1025A | | *hisA* | | 1-(5-phosphoribosyl)-5-[(5-phosphoribosylamino)methylideneamino] imidazole-4-carboxamide isomerase (EC 5.3.1.16) |
| HQ1026A | | *hisB* | | imidazoleglycerol-phosphate dehydratase (EC 4.2.1.19) |
| HQ1042A | | *thrC* | | threonine synthase (EC 4.2.3.1) |
| HQ1043A | | *serA* | | phosphoglycerate dehydrogenase (EC 1.1.1.95) |
| HQ1044A | | *serB* | | phosphoserine phosphatase (EC 3.1.3.3) |
| HQ1154A | | *aroD* | | 3-dehydroquinate dehydratase (EC 4.2.1.10) |
| HQ1155A | | *aroB* | | 3-dehydroquinate synthase (EC 4.2.3.4) |
| HQ1157A | | *trpA* | | tryptophan synthase (EC 4.2.1.20), alpha subunit |
| HQ1158A | | *trpB* | | tryptophan synthase (EC 4.2.1.20), beta subunit |
| HQ1159A | | *trpC* | | indole-3-glycerol-phosphate synthase (EC 4.1.1.48) |
| HQ1237A | | *aspC* | | PLP-dependent aminotransferase (probable aspartate aminotransferase (EC 2.6.1.1)) |
| HQ1245A | | *aroE* | | shikimate 5-dehydrogenase (EC 1.1.1.25) |
| HQ1247A | | *ilvE* | | branched-chain amino acid aminotransferase (EC 2.6.1.42) |
| HQ1292A | | *leuA* | | 2-isopropylmalate synthase (EC 2.3.3.13) |
| HQ1300A | | *hisE* | | phosphoribosyl-ATP pyrophosphatase (EC 3.6.1.31) |
| HQ1301A | | *hisH* | | imidazoleglycerol-phosphate synthase (EC 2.4.2.-) |
| HQ1348A | | *ilvA* | | threonine ammonia-lyase (EC 4.3.1.19) |
| HQ1355A | | *thrC* | | threonine synthase (EC 4.2.3.1) |
| HQ1368A | | *argE* | | acetylornithine deacetylase (EC 3.5.1.16) |
| HQ1384A | | *hisH* | | imidazole glycerol phosphate synthase (EC 2.4.2.-), glutamine amidotransferase subunit HisH |
| HQ1385A | | *pheA* | | prephenate dehydratase (EC 4.2.1.51) |
| HQ1386A | | *ocd* | | ornithine cyclodeaminase (EC 4.3.1.12) |
| HQ1488A | | *ggt* | | gamma-glutamyltransferase (EC 2.3.2.2) |
| HQ1507A | | *dapA* | | dihydrodipicolinate synthase (EC 4.2.1.52) |
| HQ1508A | | *dapB* | | dihydrodipicolinate reductase (EC 1.3.1.26) |
| HQ1509A | | *dapD* | | 2,3,4,5-tetrahydropyridine-2,6-dicarboxylate N-succinyltransferase (EC 2.3.1.117) |
| HQ1510A | | *lysA* | | diaminopimelate decarboxylase (EC 4.1.1.20) |
| HQ1511A | | *dapF* | | diaminopimelate epimerase (EC 5.1.1.7) (DAP epimerase) |
| HQ1512A | | *dapE* | | probable succinyl-diaminopimelate desuccinylase, DapE (EC 3.5.1.18) |
| HQ1564A | | *thrC* | | threonine synthase (EC 4.2.3.1) |
| HQ1632A | | *aspC* | | PLP-dependent aminotransferase (probable aspartate aminotransferase (EC 2.6.1.1) ) |
| HQ1668A | | *cysD* | | sulfate adenylyltransferase, small subunit (EC 2.7.7.4) |
| HQ1691A | | *asnB* | | asparagine synthase (glutamine-hydrolyzing) (EC 6.3.5.4) |
| HQ1692A | | *hisK* | | probable histidinol phosphatase |
| HQ1695A | | *hisF* | | imidazoleglycerol-phosphate synthase (EC 2.4.2.-) chain hisF |
| HQ1714A | | *gltB* | | glutamate synthase large subunit (EC 1.4.1.13) |
| HQ1760A | | *gcvT* | | aminomethyltransferase, glycin cleavage system T protein (EC 2.1.2.10) |
| HQ1761A | | *gcvT* | | aminomethyltransferase, glycin cleavage system T protein (EC 2.1.2.10) |
| HQ1762A | | *ilvA* | | threonine ammonia-lyase (EC 4.3.1.19) |
| HQ1783A | | *trpG* | | anthranilate synthase component II (EC 4.1.3.27) |
| HQ1784A | | *trpE* | | anthranilate synthase, component I (EC 4.1.3.27) |
| HQ1785A | | *cysE* | | serine O-acetyltransferase (EC 2.3.1.30) |
| HQ1803A | | *ocd* | | probable ornithine cyclodeaminase (EC 4.3.1.12) |
| HQ1805A | | *cre* | | creatinine amidohydrolase (EC 3.5.2.10) |
| HQ1812A | | *aspC* | | PLP-dependent aminotransferase (probable aspartate aminotransferase (EC 2.6.1.1) ) |
| HQ1844A | | *proA* | | glutamate-5-semialdehyde dehydrogenase (EC 1.2.1.41) |
| HQ1845A | | *proB* | | glutamate 5-kinase (EC 2.7.2.11) |
| HQ1846A | | *proC* | | pyrroline-5-carboxylate reductase (EC 1.5.1.2) |
| HQ1856A | | *gcvT* | | aminomethyltransferase, glycin cleavage system T protein (EC 2.1.2.10) |
| HQ1862A | | *nifS* | | probable cysteine desulfurase, class V aminotransferase (EC 2.8.1.7) |
| HQ1880A | | *gdhA* | | glutamate dehydrogenase (NADP+) (EC 1.4.1.4) |
| HQ2066A | | *serA* | | phosphoglycerate dehydrogenase (EC 1.1.1.95) |
| HQ2236A | | *metE* | | 5-methyltetrahydropteroyltriglutamate--homocysteine S-methyltransferase, methionine synthase II (EC 2.1.1.14) |
| HQ2237A | | *ocd* | | ornithine cyclodeaminase (EC 4.3.1.12) |
| HQ2241A | | *hyuB* | | N-methylhydantoinase B (ATP-hydrolyzing) (EC 3.5.2.14) |
| HQ2242A | | *hyuA* | | N-methylhydantoinase A (ATP-hydrolyzing) (EC 3.5.2.14) |
| HQ2276A | | *dap2* | | Dipeptidyl aminopeptidases/acylaminoacyl-peptidases |
| HQ2363A | | *panD* | | Aspartate 1-decarboxylase precursor (EC 4.1.1.11) (Aspartate alpha-decarboxylase) |
| HQ2365A | | *dapA* | | dihydrodipicolinate synthase (EC 4.2.1.52) |
| HQ2493A | | *dadA* | | glycine/D-amino acid oxidases (deaminating) |
| HQ2556A | | *cysK* | | cysteine synthase (EC 2.5.1.47) |
| HQ2584A | | *metK* | | methionine adenosyltransferase (EC 2.5.1.6) |
| HQ2629A | | *aspC* | | PLP-dependent aminotransferase (probable aspartate aminotransferase (EC 2.6.1.1) ) |
| HQ2700A | | *leuA* | | 2-isopropylmalate synthase (EC 2.3.3.13) |
| HQ2702A | | *ilvB* | | acetolactate synthase large subunit (EC 2.2.1.6) |
| HQ2703A | | *ilvN* | | acetolactate synthase, small subunit (EC 2.2.1.6) |
| HQ2704A | | *ilvC* | | ketol-acid reductoisomerase (EC 1.1.1.86) |
| HQ2705A | | *leuC* | | 3-isopropylmalate dehydratase large subunit (EC 4.2.1.33) |
| HQ2706A | | *leuD* | | 3-isopropylmalate dehydratase small subunit (EC 4.2.1.33) |
| HQ2707A | | *leuB* | | 3-isopropylmalate dehydrogenase (EC 1.1.1.85) |
| HQ2720A | | *hisD* | | histidinol dehydrogenase (EC 1.1.1.23) |
| HQ2762A | | *aroK* | | shikimate kinase, archaeal type (EC 2.7.1.71) |
| HQ2763A | | *pheA* | | chorismate mutase (EC 5.4.99.5) |
| HQ2774A | | *carA* | | carbamoyl-phosphate synthase, small subunit (glutamine-hydrolyzing) (EC 6.3.5.5) |
| HQ2791A | | *glyA* | | glycine hydroxymethyltransferase (EC 2.1.2.1) |
| HQ2880A | | *metE* | | 5-methyltetrahydropteroyltriglutamate--homocysteine S-methyltransferase, methionine synthase II (EC 2.1.1.14) |
| HQ2881A | | *metE* | | 5-methyltetrahydropteroyltriglutamate--homocysteine S-methyltransferase, methionine synthase II (EC 2.1.1.14) |
| HQ2908A | | *asd* | | aspartate-semialdehyde dehydrogenase (EC 1.2.1.11) |
| HQ2909A | | *serA* | | phosphoglycerate dehydrogenase (EC 1.1.1.95) |
| HQ2919A | | *agxT* | | aminotransferase class V (Serine--pyruvate aminotransferase (EC 2.6.1.51) 2; Alanine--glyoxylate aminotransferase (EC 2.6.1.44) 2) |
| HQ2926A | | *-* | | amino acid kinase |
| HQ3021A | | *ilvD* | | dihydroxy-acid dehydratase (EC 4.2.1.9) |
| HQ3036A | | *asnA* | | asparaginase (EC 3.5.1.1) |
| HQ3086A | | *gloA* | | lactoylglutathione lyase (EC 4.4.1.5) |
| HQ3112A | | *gcvT* | | aminomethyltransferase, glycin cleavage system T protein (EC 2.1.2.10) |
| HQ3166A | | *trpG* | | anthranilate synthase, component II (EC 4.1.3.27) |
| HQ3167A | | *trpE* | | anthranilate synthase (EC 4.1.3.27), component I |
| HQ3168A | | *trpF* | | phosphoribosylanthranilate isomerase (EC 5.3.1.24) |
| HQ3169A | | *trpD* | | anthranilate phosphoribosyltransferase (EC 2.4.2.18) |
| HQ3234A | | *ilvE* | | branched-chain-amino-acid transaminase (EC 2.6.1.42) |
| HQ3311A | | *carB* | | carbamoyl-phosphate synthase, large subunit (glutamine-hydrolyzing) (EC 6.3.5.5) |
| HQ3322A | | *thrB* | | homoserine kinase (EC 2.7.1.39) |
| HQ3349A | | *speB* | | agmatinase (EC 3.5.3.11) |
| HQ3352A | | *tyrA* | | prephenate dehydrogenase (EC 1.3.1.12) |
| HQ3357A | | *aroC* | | chorismate synthase (EC 4.2.3.5) |
| HQ3358A | | *aroA* | | 3-phosphoshikimate 1-carboxyvinyltransferase (EC 2.5.1.19) |
| HQ3364A | | *hisC* | | histidinol-phosphate aminotransferase (EC 2.6.1.9) |
| HQ3386A | | *hom* | | homoserine dehydrogenase (EC 1.1.1.3) |
| HQ3412A | | *hisG* | | ATP phosphoribosyltransferase (EC 2.4.2.17) |
| HQ3414A | | *achY* | | adenosylhomocysteinase (EC 3.3.1.1) |
| HQ3427A | | *nifS* | | cysteine desulfurase, class V aminotransferase (EC 2.8.1.7) |
| HQ3445A | | *glnK* | | nitrogen regulatory protein P-II |
| HQ3454A | | *metY* | | O-acetylhomoserine aminocarboxypropyltransferase (EC 2.5.1.49); methionine synthase |
| HQ3625A | | *ureB* | | urease, beta subunit (EC 3.5.1.5) |
| HQ3626A | | *ureA* | | urease, alpha subunit (EC 3.5.1.5) |
| HQ3627A | | *ureC* | | urease, gamma subunit (EC 3.5.1.5) |
| HQ3628A | | *ureG* | | urease accessory protein |
| HQ3629A | | *ureD* | | urease accessory protein |
| HQ3630A | | *ureE* | | urease accessory protein |
| HQ3631A | | *ureF* | | urease accessory protein |
| HQ3643A | | *glnA* | | glutamate--ammonia ligase (EC 6.3.1.2) |
| HQ3699A | | *metY* | | O-acetylhomoserine aminocarboxypropyltransferase (methionine synthase) (EC 2.5.1.49) |
| HQ3700A | | *metA* | | homoserine O-acetyltransferase (EC 2.3.1.31) |
| HQ3711A | | *argG* | | argininosuccinate synthase (EC 6.3.4.5) |
| HQ3712A | | *argH* | | argininosuccinate lyase (EC 4.3.2.1) |
| HQ3715A | | *argC* | | N-acetyl-gamma-glutamyl-phosphate reductase (EC 1.2.1.38) |
| HQ3716A | | *argB* | | acetylglutamate kinase (EC 2.7.2.8) |
| HQ3717A | | *argD* | | acetylornithine aminotransferase (EC 2.6.1.11) |
| HQ3718A | | *argE* | | acetylornithine deacetylase (EC 3.5.1.16) |
| HQ3719A | | *argF* | | ornithine carbamoyltransferase (EC 2.1.3.3) |
| **COM** | |  | | **coenzyme metabolism** |
| ***code*** | | ***gene*** | | ***protein name*** |
| HQ1033A | | *cobW* | | cobalamin synthesis protein/P47K |
| HQ1058A | | *pncB* | | nicotinate phosphoribosyltransferase (EC 2.4.2.11) |
| HQ1060A | | *entB* | | probable isochorismatase (EC 3.3.2.1); probable nicotinamidase (EC 3.5.1.19); probable N-carbamoylsarcosine amidase (EC 3.5.1.59) |
| HQ1091A | | *nadE* | | NAD(+) synthase (glutamine-hydrolyzing) (EC 6.3.5.1) |
| HQ1099A | | *moaE* | | molybdopterin converting factor, large subunit |
| HQ1102A | | *thiL* | | thiamin-phosphate kinase (EC 2.7.4.16) |
| HQ1164A | | *nadR* | | nicotinamide-nucleotide adenylyltransferase (EC 2.7.7.1) |
| HQ1396A | | *ribC* | | riboflavin synthase alpha chain (EC 2.5.1.9) |
| HQ1404A | | *cbiP* | | cobyric acid synthase (EC 6.3.1.-) |
| HQ1405A | | *cobO* | | cob(I)alamin adenosyltransferase (EC 2.5.1.17) |
| HQ1406A | | *cbiZ* | | Adenosylcobinamide amidohydrolase (EC 3.5.1.-) (AdoCbi hydrolase) |
| HQ1407A | | *cobD* | | L-threonine-O-3-phosphate decarboxylase (EC 4.1.1.81) |
| HQ1408A | | *cobT* | | nicotinate-nucleotide-dimethylbenzimidazole phosphoribosyltransferase (EC 2.4.2.21) |
| HQ1409A | | *cobY* | | GTP:adenosylcobinamide-phosphate guanylyltransferase |
| HQ1410A | | *cobS* | | cobalamin (5'-phosphate) synthase (EC 2.7.8.-) |
| HQ1411A | | *cbiB* | | cobalamine biosynthesis protein (adenosylcobinamide biosynthesis protein) |
| HQ1425A | | *moeB* | | molybdenum cofactor biosynthesis protein MoeB |
| HQ1426A | | *menA* | | 1,4-dihydroxy-2-naphthoate octaprenyltransferase (EC 2.5.1.-) |
| HQ1577A | | *ctaB* | | protoheme IX farnesyltransferase (EC 2.5.1.-) |
| HQ1633A | | *ribE* | | riboflavin synthase beta chain (EC 2.5.1.9) (6,7-dimethyl-8-ribityllumazine synthase) |
| HQ1683A | | *dfp* | | phosphopantothenoylcysteine decarboxylase (EC 4.1.1.36)/ phosphopantothenate--cysteine ligase (EC 6.3.2.5) |
| HQ1767A | | *folCP* | | folylpolyglutamate synthase (EC 6.3.2.17) / dihydropteroate synthase (EC 2.5.1.15) |
| HQ1829A | | *cbiA* | | cobyrinic acid a,c-diamide synthase |
| HQ1831A | | *cbiG* | | cobalamin biosynthesis protein CbiG |
| HQ1832A | | *cbiF* | | precorrin-4 C11-methyltransferase (EC 2.1.1.133) |
| HQ1833A | | *cbiL* | | precorrin-2 C20-methyltransferase (EC 2.1.1.130) |
| HQ1834A | | *cbiT* | | precorrin-8W decarboxylase (EC 1.-.-.-) |
| HQ1842A | | *folA* | | dihydrofolate reductase (EC 1.5.1.3) |
| HQ1872A | | *menF* | | isochorismate synthase (EC 5.4.4.2) |
| HQ1873A | | *menD* | | 2-oxoglutarate decarboxylase (EC 4.1.1.71); 2-succinyl-6-hydroxy-2,4-cyclohexadiene-1-carboxylate synthase (EC 4.1.3.-) |
| HQ1874A | | *menB* | | naphthoate synthase (EC 4.1.3.36) |
| HQ1875A | | *menA* | | 1,4-dihydroxy-2-naphthoate octaprenyltransferase (EC 2.5.1.-) |
| HQ1876A | | *menC* | | O-succinylbenzoate-CoA synthase (OSB synthase; 4-(2'-carboxyphenyl)- 4-oxybutyric acid synthase) |
| HQ1884A | | *ubiA* | | prenyltransferase (probable 4-hydroxybenzoate octaprenyltransferase (EC 2.5.1.-), probable protoheme IX farnesyltransferase (EC 2.5.1.-)) |
| HQ1945A | | *mobA* | | molybdopterin-guanine dinucleotide biosynthesis protein A |
| HQ1979A | | *pncB* | | nicotinate phosphoribosyltransferase (EC 2.4.2.11) |
| HQ2007A | | *moaA* | | molybdopterin-based tungsten cofactor biosynthesis protein |
| HQ2280A | | *entB* | | Isochorismatase (EC 3.3.2.1) |
| HQ2295A | | *chlID* | | magnesium chelatase (EC 6.6.1.1) (protoporphyrin IX magnesium-chelatase) |
| HQ2296A | | *cobN* | | cobalt chelatase (EC 4.99.1.-) |
| HQ2297A | | *cbiC* | | precorrin-8X methylmutase (EC 5.4.1.2) |
| HQ2298A | | *cbiE* | | precorrin-6Y C5,15-methyltransferase (decarboxylating) (EC 2.1.1.132) |
| HQ2299A | | *-* | | predicted atp- binding cobalamin adenosyltransferase |
| HQ2340A | | *moaC* | | molybdenum cofactor biosynthesis protein C |
| HQ2376A | | *mobA* | | molybdopterin-guanine dinucleotide biosynthesis protein A |
| HQ2402A | | *menE* | | O-succinylbenzoic acid--CoA ligase (EC 6.2.1.26) |
| HQ2416A | | *ubiE* | | Menaquinone biosynthesis methyltransferase UbiE (EC 2.1.1.-) |
| HQ2423A | | *ptpS* | | 6-pyruvoyltetrahydropterin synthase (4.2.3.12) |
| HQ2455A | | *folA* | | dihydrofolate reductase (EC 1.5.1.3) |
| HQ2465A | | *folP* | | dihydropteroate synthase (EC 2.5.1.15) |
| HQ2510A | | *ribG* | | 5-amino-6-(5-phosphoribosylamino)uracil reductase (EC 1.1.1.193) |
| HQ2565A | | *ppaT* | | phosphopantetheine adenylyl transferase (EC 2.7.7.3) |
| HQ2580A | | *nadE* | | NAD(+) synthase (glutamine-hydrolyzing) (EC 6.3.5.1) |
| HQ2655A | | *thiE* | | thiamine-phosphate pyrophosphorylase (EC 2.5.1.3) |
| HQ2656A | | *thiM* | | hydroxyethylthiazole kinase (EC 2.7.1.50) |
| HQ2657A | | *thiD* | | phosphomethylpyrimidine kinase (EC 2.7.4.7) |
| HQ2790A | | *folD* | | methylenetetrahydrofolate dehydrogenase (EC 1.5.1.5); methenyltetrahydrofolate cyclohydrolase (EC 3.5.4.9) |
| HQ2798A | | *mch* | | methenyltetrahydromethanopterin cyclohydrolase (EC 3.5.4.27) |
| HQ2862A | | *ubiA* | | prenyltransferase (probable 4-hydroxybenzoate octaprenyltransferase (EC 2.5.1.-), probable protoheme IX farnesyltransferase (EC 2.5.1.-)) |
| HQ2905A | | *birA* | | biotin--[acetyl-CoA-carboxylase] ligase (EC 6.3.4.15) |
| HQ2948A | | *moaA* | | molybdenum cofactor biosynthesis protein A |
| HQ3014A | | *citG* | | triphosphoribosyl-dephospho-CoA synthase (EC 2.7.8.25) |
| HQ3022A | | *moaB* | | molybdenum cofactor biosynthesis protein B |
| HQ3032A | | *cbiX* | | conserved cobalamin cluster protein CbiX (probable ferredoxin-like iron-sulfur protein) |
| HQ3033A | | *cbiH2* | | precorrin-3B C17-methyltransferase 2 (EC 2.1.1.131) |
| HQ3034A | | *cbiH1* | | precorrin-3B C17-methyltransferase 1 (EC 2.1.1.131) |
| HQ3035A | | *cbiG* | | cobalamin biosynthesis protein G, CbiG |
| HQ3042A | | *mobB* | | molybdopterin-guanine dinucleotide biosynthesis protein B |
| HQ3047A | | *coaE* | | dephospho-CoA kinase (EC 2.7.1.24) |
| HQ3121A | | *nirD* | | heme biosynthesis protein nirD/L (nirH/nirG homolog) |
| HQ3125A | | *thiH* | | thiamine biosynthesis enzyme ThiH; biotin biosynthesis protein BioB? |
| HQ3127A | | *thiH* | | thiamine biosynthesis enzyme ThiH; biotin biosynthesis protein BioB? |
| HQ3189A | | *cobW* | | cobalamin synthesis protein/P47K |
| HQ3199A | | *moaD* | | molybdopterin converting factor, small subunit |
| HQ3216A | | *nadA* | | quinolinate synthase A (EC 4.1.99.-) |
| HQ3217A | | *nadB* | | L-aspartate oxidase (EC 1.4.3.16), quinolinate synthetase subunit B |
| HQ3218A | | *nadC* | | nicotinate-nucleotide pyrophosphorylase (carboxylating) (EC 2.4.2.19) |
| HQ3235A | | *ribB* | | 3,4-dihydroxy-2-butanone 4-phosphate synthase (EC 5.4.99.-) |
| HQ3321A | | *hemY* | | protoporphyrinogen oxidase (EC 1.3.3.4) |
| HQ3323A | | *pyroA* | | pyridoxine biosynthesis enzyme |
| HQ3334A | | *nirH* | | heme biosynthesis protein nirH/G (nirL/nirD homolog) |
| HQ3335A | | *cysG* | | siroheme synthase (precorrin-2 oxidase (EC 1.3.1.76) and ferrochelatase (EC 4.99.1.4)) (N-terminal domain homology to multifunctional siroheme synthase CysG of Escherichia coli) |
| HQ3336A | | *hemA* | | glutamyl-tRNA reductase (EC 1.2.1.-) |
| HQ3342A | | *cad* | | 4a-hydroxytetrahydrobiopterin dehydratase (EC 4.2.1.96) |
| HQ3344A | | *moeA* | | molybdenum cofactor biosynthesis protein MoeA; periplasmic molybdate-binding domain |
| HQ3345A | | *moeA* | | molybdenum cofactor biosynthesis protein MoeA |
| HQ3347A | | *ubiB* | | probable ubiquinone biosynthesis transmembrane protein |
| HQ3443A | | *hemB* | | porphobilinogen synthase (EC 4.2.1.24) |
| HQ3447A | | *hemL* | | glutamate-1-semialdehyde 2,1-aminomutase (EC 5.4.3.8) |
| HQ3450A | | *hemC* | | hydroxymethylbilane synthase (EC 2.5.1.61) (porphobilinogen deaminase) |
| HQ3451A | | *hemX* | | uroporphyrin-III C-methyltransferase (EC 2.1.1.107) |
| HQ3452A | | *hemD* | | uroporphyrinogen-III synthase (EC 4.2.1.75) |
| **NUM** | |  | | **nucleotide metabolism** |
| ***code*** | | ***gene*** | | ***protein name*** |
| HQ1006A | | *purH* | | IMP cyclohydrolase (EC 3.5.4.10), archaeal type |
| HQ1029A | | *upp* | | uracil phosphoribosyltransferase (EC 2.4.2.9) |
| HQ1052A | | *pyrD* | | dihydroorotate oxidase (EC 1.3.3.1) |
| HQ1061A | | *pyrC* | | dihydroorotase (EC 3.5.2.3) |
| HQ1098A | | *pyrH* | | uridylate kinase (EC 2.7.4.-) |
| HQ1262A | | *mtaP* | | 5'-methylthioadenosine phosphorylase (EC 2.4.2.28) MtaP |
| HQ1278A | | *dcd* | | dCTP deaminase (EC 3.5.4.13) |
| HQ1289A | | *guaAa* | | GMP synthase (glutamine-hydrolyzing) (EC 6.3.5.2), subunit A |
| HQ1343A | | *ham1* | | Nucleoside-triphosphatase (EC 3.6.1.15) |
| HQ1449A | | *adkA* | | adenylate kinase (ATP-AMP transphosphorylase) (EC 2.7.4.3) archaeal type |
| HQ1464A | | *purT* | | phosphoribosylglycinamide formyltransferase (EC 2.1.2.-) 2; formate-dependent GAR transformylase |
| HQ1635A | | *purK* | | phosphoribosylaminoimidazole carboxylase (EC 4.1.1.21) carbon dioxide-fixation chain |
| HQ1636A | | *purE* | | phosphoribosylaminoimidazole carboxylase (EC 4.1.1.21) catalytic chain |
| HQ1660A | | *purA* | | adenylosuccinate synthase (EC 6.3.4.4) |
| HQ1661A | | *purNH* | | phosphoribosylglycinamide formyltransferase (EC 2.1.2.2)/ phosphoribosylaminoimidazolecarboxamide formyltransferase (EC 2.1.2.3) |
| HQ1662A | | *purB* | | adenylosuccinate lyase (EC 4.3.2.2) |
| HQ1664A | | *pyrE* | | orotate phosphoribosyltransferase (EC 2.4.2.10) |
| HQ1672A | | *apt* | | purine phosphoribosyltransferase (adenine phosphoribosyltransferase (EC 2.4.2.7), xanthine-guanine phosphoribosyltransferase (EC 2.4.2.22)) |
| HQ1693A | | *purL* | | phosphoribosylformylglycinamidine synthase (EC 6.3.5.3), component II |
| HQ1768A | | *fhs* | | formyltetrahydrofolate synthetase |
| HQ1795A | | *tdk* | | thymidine kinase (EC 2.7.1.21) |
| HQ1878A | | *pyrI* | | aspartate carbamoyltransferase, regulatory subunit (EC 2.1.3.2) |
| HQ1879A | | *pyrB* | | aspartate carbamoyltransferase, catalytic subunit (EC 2.1.3.2) |
| HQ1892A | | *udp* | | uridine phosphorylase (EC 2.4.2.3) |
| HQ1936A | | *pyrC* | | dihydroorotase (EC 3.5.2.3) |
| HQ1998A | | *dcd* | | deoxycytidine triphosphate deaminase |
| HQ2049A | | *guaD* | | probable nucleoside deaminase; cytosine deaminase (EC 3.5.4.1); guanine deaminase (EC 3.5.4.3) |
| HQ2050A | | *apt* | | purine phosphoribosyltransferase (adenine phosphoribosyltransferase (EC 2.4.2.7), xanthine-guanine phosphoribosyltransferase (EC 2.4.2.22)) |
| HQ2382A | | *guaB* | | IMP dehydrogenase (EC 1.1.1.205)/ CBS domain protein |
| HQ2427A | | *gch3* | | GTP cyclohydrolase III (EC 3.5.4.-) |
| HQ2456A | | *thyA* | | thymidylate synthase (EC 2.1.1.45) |
| HQ2498A | | *iunH* | | Inosine-uridine preferring nucleoside hydrolase (EC 3.2.2.1) |
| HQ2560A | | *purD* | | phosphoribosylamine--glycine ligase (EC 6.3.4.13) |
| HQ2620A | | *guaD* | | probable nucleoside deaminase (cytosine deaminase (EC 3.5.4.1), guanine deaminase (EC 3.5.4.3)) |
| HQ2646A | | *cdd* | | cytidine deaminase (EC 3.5.4.5) |
| HQ2647A | | *udp* | | uridine phosphorylase (EC 2.4.2.3) |
| HQ2665A | | *purM* | | phosphoribosylformylglycinamidine cyclo-ligase (EC 6.3.3.1) |
| HQ2723A | | *ushA* | | 5'-nucleotidase (EC 3.1.3.5); 2',3'-cyclic-nucleotide 2'-phosphodiesterase (EC 3.1.4.16); UDP-sugar hydrolase (EC 3.6.1.45) |
| HQ2740A | | *cmk* | | cytidylate kinase (EC 2.7.4.14) |
| HQ2765A | | *cmk* | | cytidylate kinase (EC 2.7.4.14) |
| HQ2767A | | *adk* | | adenylate kinase (EC 2.7.4.3) |
| HQ2859A | | *nrdA* | | ribonucleoside-diphosphate reductase (EC 1.17.4.1) alpha subunit (intein-containing) |
| HQ2882A | | *ndk* | | nucleoside-diphosphate kinase (EC 2.7.4.6) |
| HQ2893A | | *purF* | | amidophosphoribosyltransferase (EC 2.4.2.14) |
| HQ3010A | | *guaD* | | probable nucleoside deaminase (cytosine deaminase (EC 3.5.4.1), guanine deaminase (EC 3.5.4.3)) |
| HQ3116A | | *dcd* | | probable dCTP deaminase (EC 3.5.4.13) |
| HQ3128A | | *purC* | | phosphoribosylaminoimidazole-succinocarboxamide synthase (EC 6.3.2.6) |
| HQ3129A | | *purU* | | formyltetrahydrofolate deformylase (EC 3.5.1.10) |
| HQ3130A | | *purS* | | phosphoribosylformylglycinamidine synthase, PurS component (EC 6.3.5.3) |
| HQ3131A | | *purQ* | | phosphoribosylformylglycinamidine synthase component I (EC 6.3.5.3) |
| HQ3145A | | *tmk* | | thymidylate kinase (EC 2.7.4.9) |
| HQ3163A | | *nudF* | | ADP-ribose pyrophosphatase (EC 3.6.1.13); mut/nudix family protein |
| HQ3194A | | *guaAb* | | GMP synthase, subunit B (glutamine-hydrolyzing) (EC 6.3.5.2) |
| HQ3195A | | *pyrG* | | CTP synthase (EC 6.3.4.2) |
| HQ3201A | | *udp* | | uridine phosphorylase (EC 2.4.2.3) |
| HQ3219A | | *pyrF* | | orotidine-5'-phosphate decarboxylase (EC 4.1.1.23) |
| HQ3228A | | *apt* | | purine phosphoribosyltransferase 2 (adenine phosphoribosyltransferase (EC 2.4.2.7), xanthine-guanine phosphoribosyltransferase (EC 2.4.2.22)) |
| HQ3229A | | *pyrE* | | orotate phosphoribosyltransferase (EC 2.4.2.10) |
| HQ3363A | | *-* | | predicted nucleotide kinase (EC 2.7.4.-) |
| HQ3374A | | *cumB* | | cytosine/adenosine deaminases |
| HQ3508A | | *nudF* | | ADP-ribose pyrophosphatase (EC 3.6.1.13) |
| HQ3650A | | *prsA* | | ribose-phosphate pyrophosphokinase (EC 2.7.6.1) |
| **LIP** | |  | | **lipid metabolism** |
| ***code*** | | ***gene*** | | ***protein name*** |
| HQ1018A | | *crtY* | | lycopene cyclase (EC 1.14.-.-) |
| HQ1120A | | *-* | | probable ferredoxin-NAD+ reductase (EC 1.18.1.3) |
| HQ1269A | | *pcrB* | | putative (S)-3-O-geranylgeranylglyceryl phosphate (GGGP) synthase |
| HQ1525A | | *mvaD* | | diphosphomevalonate decarboxylase (EC 4.1.1.33) |
| HQ1538A | | *acs* | | acyl-CoA synthetase I (EC 6.2.1.1) |
| HQ1565A | | *crtI* | | phytoene dehydrogenase (phytoene desaturase) (EC 1.14.99.-) |
| HQ1583A | | *-* | | phospholipase D |
| HQ1590A | | *acaB* | | acetyl-CoA C-acyltransferase (EC 2.3.1.16) |
| HQ1699A | | *alkK* | | acyl-CoA synthetase (EC 6.2.1.-) II; probable long-chain-fatty-acid--CoA ligase (EC 6.2.1.3) |
| HQ1730A | | *pgsA* | | CDP-diacylglycerol--glycerol-3-phosphate 3-phosphatidyl-transferase (Phosphatidylglycerophosphate synthase) (PGP synthase) (EC 2.7.8.5) |
| HQ1794A | | *crtI* | | phytoene dehydrogenase (EC 1.14.99.-) (phytoene desaturase) |
| HQ1985A | | *acaB* | | acetyl-CoA C-acyltransferase (EC 2.3.1.16) |
| HQ2020A | | *-* | | beta,beta-carotene 9',10'-dioxygenase (EC 1.14.99.-) 2 |
| HQ2118A | | *ugpQ* | | glycerophosphodiester phosphodiesterase (EC 3.1.4.46) |
| HQ2301A | | *mcmB* | | methylmalonyl-CoA mutase, subunit B (cobalamin-binding subunit) (EC 5.4.99.2) |
| HQ2302A | | *hbd* | | 3-hydroxyacyl-CoA dehydrogenase (EC 1.1.1.35) |
| HQ2307A | | *acaB* | | acetyl-CoA C-acyltransferase (EC 2.3.1.16) |
| HQ2315A | | *maoC* | | maoC protein homolog; probable enoyl-CoA hydratase |
| HQ2353A | | *fdfT* | | farnesyl-diphosphate farnesyltransferase (EC 2.5.1.21) (squalene synthase) |
| HQ2356A | | *pssA* | | CDP-diacylglycerol--serine O-phosphatidyltransferase (EC 2.7.8.8) (Phosphatidylserine synthase) |
| HQ2379A | | *ugpQ* | | glycerophosphodiester phosphodiesterase (EC 3.1.4.46) |
| HQ2381A | | *-* | | beta,beta-carotene 15,15'-monooxygenase (EC 1.14.99.36); beta-carotene dioxygenase 1 |
| HQ2389A | | *acd* | | acyl-CoA dehydrogenase (EC 1.3.99.3) |
| HQ2394A | | *fadA* | | enoyl-CoA hydratase I (EC 4.2.1.17) |
| HQ2400A | | *mcmA* | | methylmalonyl-CoA mutase, subunit A (EC 5.4.99.2) |
| HQ2523A | | *pssA* | | CDP-diacylglycerol--serine O-phosphatidyltransferase (EC 2.7.8.8) (Phosphatidylserine synthase) |
| HQ2772A | | *idiA* | | isopentenyl-diphosphate delta-isomerase (EC 5.3.3.2) |
| HQ2847A | | *idiB* | | isopentenyl-diphosphate delta-isomerase (EC 5.3.3.2) |
| HQ2860A | | *crtB* | | geranylgeranyl-diphosphate geranylgeranyltransferase (EC 2.5.1.32) (phytoene synthase) |
| HQ2863A | | *crtI* | | phytoene dehydrogenase (EC 1.14.99.-) (phytoene desaturase) |
| HQ2868A | | *mvaB* | | hydroxymethylglutaryl-CoA synthase (EC 2.3.3.10) |
| HQ2889A | | *idsA* | | probable trifunctional short-chain (E)-prenyl diphosphate synthase (probable dimethylallyltransferase (EC 2.5.1.1) / probable geranyltranstransferase (EC 2.5.1.10) / probable farnesytranstransferase (EC 2.5.1.29)) |
| HQ2906A | | *accA2* | | biotin carboxylase (EC 6.3.4.14) 1, acyl-CoA carboxylase, alpha subunit 2 |
| HQ2925A | | *mvk* | | mevalonate kinase (EC 2.7.1.36) |
| HQ3007A | | *-* | | beta,beta-carotene 9',10'-dioxygenase (EC 1.14.99.-) 2 |
| HQ3018A | | *accA* | | acetyl-CoA carboxylase alpha subunit |
| HQ3215A | | *hmgR* | | hydroxymethylglutaryl-CoA reductase (NADPH) (EC 1.1.1.34) |
| HQ3230A | | *cdsA* | | phosphatidate cytidylyltransferase (EC 2.7.7.41) |
| HQ3255A | | *idsA* | | probable multifunctional long-chain (E)-prenyl diphosphate synthase (EC 2.5.1.-) |
| HQ3319A | | *hcaD* | | ferredoxin:NAD+ oxidoreductase (EC 1.18.1.3) |
| HQ3331A | | *uppS* | | di-trans,poly-cis-decaprenylcistransferase (EC 2.5.1.31) |
| HQ3332A | | *uppS* | | di-trans,poly-cis-decaprenylcistransferase (EC 2.5.1.31) |
| HQ3362A | | *pgsA* | | probable CDP-diacylglycerol--glycerol-3-phosphate 3-phosphatidyltransferase (Phosphatidylglycerophosphate synthase) (PGP synthase) (EC 2.7.8.5) |
| HQ3406A | | *psd* | | phosphatidylserine decarboxylase (EC 4.1.1.65) |
| **CHM** | |  | | **carbohydrate metabolism** |
| ***code*** | | ***gene*** | | ***protein name*** |
| HQ1024A | | *pmm* | | phosphohexomutase (phosphoglucomutase (EC 5.4.2.2); phosphomannomutase (EC 5.4.2.8)) |
| HQ1495A | | *eda* | | probable KHG/KDPG aldolase (4-hydroxy-2-oxoglutarate aldolase (EC 4.1.3.16); 2-dehydro-3-deoxy-phosphogluconate aldolase (EC 4.1.2.14)) |
| HQ1521A | | *pmm3* | | phosphohexomutase 3 (phosphoglucomutase (EC 5.4.2.2)); phosphomannomutase (EC 5.4.2.8) |
| HQ1522A | | *pmm3* | | phosphohexomutase 3 (phosphoglucomutase (EC 5.4.2.2)); phosphomannomutase (EC 5.4.2.8)) |
| HQ1527A | | *mgsA* | | methylglyoxal synthase (EC 4.2.3.3) |
| HQ1663A | | *gdh* | | Glucose 1-dehydrogenase (EC 1.1.1.47) |
| HQ1670A | | *graD* | | sugar nucleotidyltransferase (probable glucose-1-phosphate thymidylyltransferase (EC 2.7.7.24)) |
| HQ1720A | | *citE* | | citryl-CoA lyase (EC 4.1.3.34) |
| HQ1911A | | *malA* | | alpha-glucosidases, family 31 of glycosyl hydrolases |
| HQ2270A | | *nagD* | | Putative N-acetyl-glucosamine catabolism protein |
| HQ2341A | | *-* | | predicted sugar kinase (EC 2.7.1.-) |
| HQ2494A | | *galE* | | nucleoside-diphosphate-sugar epimerase (probable UDP-glucose 4-epimerase (EC 5.1.3.2)) |
| HQ2499A | | *-* | | hexosyltransferase |
| HQ2500A | | *-* | | hexosyltransferase 1 |
| HQ2539A | | *sga1* | | glucoamylase |
| HQ2540A | | *amyA* | | alpha-amylase (EC 3.2.1.-) (Glucan 1,4-alpha-maltohydrolase) |
| HQ2578A | | *malQ* | | 4-alpha-glucanotransferase |
| HQ2650A | | *galE* | | nucleoside-diphosphate-sugar epimerase (probable UDP-glucose 4-epimerase (EC 5.1.3.2)) |
| HQ2672A | | *dhaK1* | | dihydroxyacetone kinase (EC 2.7.1.29) |
| HQ2673A | | *dhaK2* | | dihydroxyacetone kinase (EC 2.7.1.29) |
| HQ2674A | | *dhaM* | | probable phosphoenolpyruvate-protein phosphoryltransferase |
| HQ2680A | | *ugd* | | UDP-glucose 6-dehydrogenase (EC 1.1.1.22) |
| HQ2682A | | *graD* | | sugar nucleotidyltransferase II (probable glucose-1-phosphate thymidylyltransferase (EC 2.7.7.24)) |
| HQ2683A | | *galE* | | nucleoside-diphosphate-sugar epimerase 1 (probable UDP-glucose 4-epimerase (EC 5.1.3.2)) |
| HQ2694A | | *gtl* | | glycosyltransferase (probable dolichyl-phosphate beta-D-mannosyltransferase (EC 2.4.1.83)) |
| HQ2710A | | *gatY* | | fructose/tagatose- 1,6-bisphosphate aldolase |
| HQ2923A | | *celM* | | probable cellulase (EC 3.2.1.4); probable endo-1,3(4)-beta-glucanase (EC 3.2.1.6) |
| HQ2985A | | *galE* | | nucleoside-diphosphate-sugar epimerase (probable UDP-glucose 4-epimerase (EC 5.1.3.2)) |
| HQ2986A | | *galE* | | nucleoside-diphosphate-sugar epimerase (probable UDP-glucose 4-epimerase (EC 5.1.3.2)) |
| HQ2989A | | *graD* | | glucose-1-phosphate thymidylyltransferase (EC 2.7.7.24) |
| HQ2990A | | *glmS* | | glutamine--fructose-6-phosphate transaminase (isomerizing) (EC 2.6.1.16) |
| HQ2992A | | *graD* | | glucose-1-phosphate thymidylyltransferase (EC 2.7.7.24) |
| HQ3094A | | *aceA* | | isocitrate lyase (EC 4.1.3.1) |
| HQ3114A | | *pdaD* | | pyruvoyl-dependent arginine decarboxylase (EC 4.1.1.19) |
| HQ3202A | | *gfoR* | | glucose--fructose oxidoreductase (EC 1.1.99.28) |
| HQ3261A | | *manC* | | mannose-1-phosphate guanylyltransferase (EC 2.7.7.13) |
| HQ3371A | | *pho* | | probable sugar phosphatase (EC 3.1.3.-) |
| HQ3507A | | *graD* | | sugar nucleotidyltransferase II (probable glucose-1-phosphate thymidylyltransferase (EC 2.7.7.24)) |
| HQ3509A | | *galE* | | nucleoside-diphosphate-sugar epimerase (probable UDP-glucose 4-epimerase (EC 5.1.3.2)) |
| HQ3510A | | *galE* | | nucleoside-diphosphate-sugar epimerase (probable UDP-glucose 4-epimerase (EC 5.1.3.2)) |
| HQ3519A | | *neuB* | | sialic acid synthase (N-acetylneuraminate synthase) (EC 2.5.1.56) (N-acetylneuraminic acid synthase) |
| HQ3655A | | *fbp* | | probable fructose-2,6-bisphosphatase |
| HQ3673A | | *pmm* | | phosphohexomutase (phosphoglucomutase (EC 5.4.2.2); phosphomannomutase (EC 5.4.2.8)) |
| **TP_CP** | |  | | **transport and cellular processes** |
| **SEC** | |  | | **protein secretion** |
| ***code*** | | ***gene*** | | ***protein name*** |
| HQ1002A | | *sec11* | | signal sequence peptidase (EC 3.4.-.-) |
| HQ1190A | | *virB11* | | type II/IV secretion system proteins VirB11/TadA (predicted ATPase) |
| HQ1242A | | *secE* | | preprotein translocase subunit secE |
| HQ1302A | | *secG* | | preprotein translocase subunit secG |
| HQ1328A | | *tadA* | | type II/IV secretion system protein, ATP binding protein |
| HQ1329A | | *tadC* | | type II secretion system, transmembrane protein TadC |
| HQ1503A | | *srp19* | | signal recognition particle 19K protein |
| HQ1595A | | *lepW* | | probable signal peptidase I (EC 3.4.21.89) (SPase I) (Leader peptidase I) |
| HQ2505A | | *tatA* | | sec-independent protein translocase component TatA |
| HQ2819A | | *secY* | | preprotein translocase subunit secY |
| HQ3097A | | *secD* | | preprotein translocase subunit secD |
| HQ3098A | | *secF* | | preprotein translocase subunit secF |
| HQ3289A | | *trsE* | | transfer complex protein homolog |
| HQ3418A | | *srp54* | | signal recognition particle 54K protein |
| HQ3419A | | *ftsY* | | signal recognition particle receptor SRalpha |
| HQ3683A | | *tatC1* | | sec-independent protein translocase component TatC1 |
| HQ3684A | | *tatC2* | | sec-independent protein translocase component TatC2 |
| **TP** | |  | | **small molecule transport** |
| ***code*** | | ***gene*** | | ***protein name*** |
| HQ1035A | | *arsA* | | transport ATPase (EC 3.6.3.16) (probable substrate arsenite) |
| HQ1083A | | *capC* | | probable capsule polysaccharide biosynthesis protein, integral membrane protein |
| HQ1119A | | *copA* | | transport ATPase (EC 3.6.3.-) 1 (probable substrates copper/metal cation) |
| HQ1125A | | *cat2* | | cationic amino acid transport protein |
| HQ1126A | | *trkA* | | K+ transport systems, NAD-binding protein |
| HQ1127A | | *oxlT* | | major facilitator superfamily oxalate/formate antiporter |
| HQ1138A | | *-* | | ABC-type multidrug/lipid transport system, permease and ATP-binding protein |
| HQ1143A | | *nosY* | | ABC-type copper transport system involved in multi-copper enzyme maturation, permease protein |
| HQ1144A | | *nosY* | | ABC-type copper transport system involved in multi-copper enzyme maturation, permease protein |
| HQ1145A | | *nosF* | | ABC-type copper transport system involved in multi-copper enzyme maturation, ATP-binding protein |
| HQ1147A | | *pch* | | voltage-gated potassium channel (MVP) |
| HQ1152A | | *-* | | probable ABC-type transport system periplasmic substrate-binding protein (probable substrate iron(III)) |
| HQ1175A | | *-* | | transporter of the drug/metabolite transporter (DMT) superfamily |
| HQ1215A | | *-* | | RND superfamily exporter |
| HQ1225A | | *-* | | sodium/arsenite symporter family protein |
| HQ1236A | | *arsA* | | transport ATPase (EC 3.6.3.16) (probable substrate arsenite) |
| HQ1261A | | *-* | | predicted transporter |
| HQ1295A | | *-* | | ABC-type transport system, ATP-binding protein |
| HQ1296A | | *-* | | ABC-type transport system, permease protein |
| HQ1306A | | *dppF* | | ABC-type dipeptide/oligopeptide/nickel transport system, ATPase protein II |
| HQ1307A | | *dppD* | | ABC-type dipeptide/oligopeptide/nickel transport system, ATPase protein I |
| HQ1308A | | *dppC* | | ABC-type dipeptide/oligopeptide/nickel transport system, permease protein II |
| HQ1309A | | *dppB* | | ABC-type dipeptide/oligopeptide/nickel transport system, permease protein I |
| HQ1310A | | *dppA* | | ABC-type dipeptide/oligopeptide/nickel transport system, substrate binding protein |
| HQ1313A | | *cat2* | | probable cationic amino acid transport protein |
| HQ1314A | | *trkA* | | Trk-type potassium transport system, NAD-binding subunit |
| HQ1345A | | *trkA* | | potassium transport system, NAD-binding protein |
| HQ1381A | | *phnE* | | ABC-type phosphate/phosphonate/phosphite transport system, permease protein |
| HQ1382A | | *phnC* | | ABC-type phosphate/phosphonate/phosphite transport system, ATP-binding protein |
| HQ1383A | | *phnD* | | ABC-type phosphate/phosphonate/phosphite transport system, substrate-binding protein |
| HQ1419A | | *kef* | | Kef-type K+ transport systems, predicted NAD-binding component |
| HQ1421A | | *cysA* | | ABC-type transport system, ATP-binding domain (probable substrates sulfate (EC 3.6.3.25), thiosulfate or molybdate) |
| HQ1422A | | *modB* | | ABC-type transport system, permease domain (probable substrates sulfate, thiosulfate or molybdate) |
| HQ1423A | | *modA* | | ABC-type transport system, substrate binding domain (probable substrates sulfate, thiosulfate or molybdate) |
| HQ1431A | | *potA* | | ABC-type spermidine/putrescine transport systems, ATPase domain |
| HQ1432A | | *potB* | | ABC-type spermidine/putrescine transport system, permease domain I |
| HQ1433A | | *potC* | | ABC-type spermidine/putrescine transport system, permease domain II |
| HQ1434A | | *potD* | | ABC-type spermidine/putrescine transport system, substrate-binding domain |
| HQ1442A | | *Imp* | | predicted TRAP-type transport system, periplasmic protein |
| HQ1443A | | *-* | | predicted TRAP-type transport system protein |
| HQ1444A | | *-* | | predicted TRAP-type transport system, permease protein |
| HQ1451A | | *focA* | | probable formate/nitrite transport protein |
| HQ1467A | | *narK* | | nitrate/nitrite transporter |
| HQ1468A | | *pstB* | | ABC-type phosphate transport system, ATP-binding protein (EC 3.6.3.27) |
| HQ1469A | | *pstA* | | ABC-type phosphate transport system, permease protein I |
| HQ1470A | | *pstC* | | ABC-type phosphate transport system, permease protein II |
| HQ1471A | | *pstS* | | ABC-type phosphate transport system, substrate binding protein |
| HQ1477A | | *dppF* | | ABC-type dipeptide/oligopeptide/nickel transport system, ATPase protein II |
| HQ1478A | | *dppD* | | ABC-type dipeptide/oligopeptide/nickel transport system, ATPase protein I |
| HQ1479A | | *dppC* | | ABC-type dipeptide/oligopeptide/nickel transport system, permease components II |
| HQ1480A | | *dppB* | | ABC-type dipeptide/oligopeptide/nickel transport system, permease protein I |
| HQ1481A | | *dppA* | | ABC-type dipeptide/oligopeptide/nickel transport systems, substrate-binding protein |
| HQ1482A | | *dppF* | | ABC-type dipeptide/oligopeptide/nickel transport system, ATPase protein II |
| HQ1483A | | *dppD* | | ABC-type dipeptide/oligopeptide/nickel transport system, ATPase protein I |
| HQ1484A | | *dppC* | | ABC-type dipeptide/oligopeptide/nickel transport system, permease protein II |
| HQ1485A | | *dppB* | | ABC-type dipeptide/oligopeptide/nickel transport system, permease protein I |
| HQ1486A | | *dppA* | | ABC-type dipeptide/oligopeptide/nickel transport system, substrate binding protein |
| HQ1487A | | *oxlT* | | probable oxalate/formate antiporter |
| HQ1493A | | *abcA* | | ABC-type transport system ATP-binding protein |
| HQ1494A | | *abcP* | | ABC-type transport system permease protein |
| HQ1539A | | *-* | | probable substrate efflux/proton antiporter |
| HQ1542A | | *-* | | probable substrate efflux/proton antiporter |
| HQ1561A | | *lysE* | | LysE family L-lysine efflux protein/ threonine/homoserine/homoserine lactone efflux protein |
| HQ1585A | | *fhuD* | | ABC-type Fe3+-hydroxamate transport system, substrate-binding protein |
| HQ1615A | | *livF* | | ABC-type branched-chain amino acid transport systems, ATP-binding protein II |
| HQ1616A | | *livG* | | ABC-type branched-chain amino acid transport systems, ATP-binding protein I |
| HQ1617A | | *livM* | | ABC-type branched-chain amino acid transport system, permease protein II |
| HQ1618A | | *livH* | | ABC-type branched-chain amino acid transport system, permease protein I |
| HQ1619A | | *livJ* | | ABC-type branched-chain amino acid transport systems, substrate-binding protein |
| HQ1626A | | *hemV* | | ABC-type cobalamin/Iron(III)-siderophore transport systems, ATP-binding protein |
| HQ1627A | | *hemU* | | ABC-type cobalamin/Iron(III)-siderophore transport systems, permease protein |
| HQ1628A | | *hemT* | | ABC-type cobalamin/Iron(III)-siderophore transport systems, substrate-binding protein |
| HQ1674A | | *phaE* | | pH adaptation potassium efflux system protein E; sodium-potassium/hydrogen antiporter subunit E |
| HQ1675A | | *phaF* | | pH adaptation potassium efflux system protein F; sodium-potassium/hydrogen antiporter subunit F |
| HQ1676A | | *phaG* | | pH adaptation potassium efflux system protein G; sodium-potassium/hydrogen antiporter subunit G |
| HQ1677A | | *phaB1* | | pH adaptation potassium efflux system protein B1; sodium-potassium/hydrogen antiporter subunit B1 |
| HQ1678A | | *phaB2* | | pH adaptation potassium efflux system protein B2; sodium-potassium/hydrogen antiporter subunit B2 |
| HQ1679A | | *phaC* | | pH adaptation potassium efflux system protein C; sodium-potassium/hydrogen antiporter subunit C |
| HQ1680A | | *phaD* | | pH adaptation potassium efflux system protein D; sodium-potassium/hydrogen antiporter subunit D |
| HQ1681A | | *phaA* | | pH adaptation potassium efflux system protein A; sodium-potassium/hydrogen antiporter subunit A |
| HQ1682A | | *-* | | NADH-ubiquinone/plastoquinone |
| HQ1685A | | *trkA* | | Trk-type potassium transport system, NAD-binding subunit |
| HQ1686A | | *trkH* | | Trk-type potassium transport system, permease protein TrkH |
| HQ1706A | | *sufC* | | ABC-type transport system involved in Fe-S cluster assembly, ATP-binding protein |
| HQ1707A | | *sufB* | | ABC-type transport system involved in Fe-S cluster assembly, permease protein I |
| HQ1708A | | *sufB* | | ABC-type transport system involved in Fe-S cluster assembly, permease protein II |
| HQ1765A | | *opuD* | | probable glycine betaine transport protein |
| HQ1786A | | *-* | | ABC-type transport system ATP-binding protein |
| HQ1814A | | *salX* | | ABC-type antimicrobial peptide transport system, ATP-binding protein |
| HQ1815A | | *salY* | | ABC-type antimicrobial peptide transport system, permease protein |
| HQ1835A | | *cbiM* | | CbiM protein (probable ABC-type cobalt transport system permease protein 2) |
| HQ1836A | | *cbiN* | | CbiN protein (probable ABC-type cobalt transport system, periplasmic component) |
| HQ1837A | | *cbiQ* | | CbiQ protein (probable ABC-type cobalt transport system permease protein 1) |
| HQ1838A | | *cbiO* | | CbiO protein ( probable ABC-type cobalt transport system ATP-binding protein) |
| HQ1841A | | *-* | | major facilitator superfamily transporter |
| HQ1850A | | *norM* | | sodium-driven multidrug efflux pump |
| HQ1888A | | *kch* | | Kef-type K+ transport systems, predicted NAD-binding component |
| HQ1934A | | *xup* | | xanthine/uracil permease family protein. probable substrate ascorbate |
| HQ1950A | | *-* | | amino acid transporter |
| HQ1951A | | *trkA* | | Trk-type potassium transport system, NAD-binding subunit |
| HQ1952A | | *kch* | | Kef-type potassium transport systems, predicted NAD-binding protein |
| HQ1954A | | *mntH* | | NRAMP-family transporter (probable substrate manganese) |
| HQ1955A | | *-* | | sodium/anion symporter (probable sulfate or citrate) |
| HQ1956A | | *trkA* | | Trk-type potassium transport system, NAD-binding subunit |
| HQ1969A | | *-* | | ABC-type multidrug transport system, ATP-binding and permease protein |
| HQ1974A | | *tauA* | | ABC-type nitrate/sulfonate/bicarbonate transport system, substrate binding protein |
| HQ1975A | | *tauC* | | ABC-type nitrate/sulfonate/bicarbonate transport system, permease protein |
| HQ1976A | | *tauB* | | ABC-type nitrate/sulfonate/bicarbonate transport system, ATP-binding protein |
| HQ1977A | | *tauC* | | ABC-type nitrate/sulfonate/bicarbonate transport system, permease protein |
| HQ1986A | | *-* | | major facilitator superfamily transporter |
| HQ1989A | | *ugpC* | | ABC-type sugar/sn-glycerol-3-phosphate transport system, ATP-binding protein |
| HQ1990A | | *ugpE* | | ABC-type sugar/sn-glycerol-3-phosphate transport system, periplasmic protein |
| HQ1991A | | *ugpA* | | ABC-type sugar/sn-glycerol-3-phosphate transport system, periplasmic protein |
| HQ1992A | | *ugpB* | | ABC-type sugar/sn-glycerol-3-phosphate transport system, periplasmic protein |
| HQ2018A | | *salX* | | ABC-type antimicrobial peptide transport system, ATP-binding protein |
| HQ2019A | | *salY* | | ABC-type antimicrobial peptide transport system, permease protein |
| HQ2023A | | *mntH* | | NRAMP family Mn2+/Fe2+ transporters |
| HQ2052A | | *pitA* | | phosphate permease homolog |
| HQ2064A | | *rhtB* | | threonine/homoserine/homoserine lactone efflux protein |
| HQ2096A | | *uraA* | | xanthine/uracil permeases |
| HQ2127A | | *chrA* | | chromate transport protein |
| HQ2184A | | *dctM* | | TRAP-type C4-dicarboxylate transport system, large permease component DctM (YiaN) |
| HQ2185A | | *dctQ* | | TRAP-type C4-dicarboxylate transport system, small permease component |
| HQ2186A | | *dctP* | | TRAP-type C4-dicarboxylate transport system, periplasmic component DctP (YiaO) |
| HQ2189A | | *sdt* | | sodium-dependent transporter (probable antiporter) |
| HQ2192A | | *livJ* | | ABC-type branched-chain amino acid transport systems, substrate-binding protein |
| HQ2193A | | *livF* | | ABC-type branched-chain amino acid transport systems, ATP-binding protein II |
| HQ2194A | | *livF* | | ABC-type branched-chain amino acid transport systems, ATP-binding protein II |
| HQ2195A | | *livG* | | ABC-type branched-chain amino acid transport systems, ATP-binding protein I |
| HQ2196A | | *livM* | | ABC-type branched-chain amino acid transport system, permease protein II |
| HQ2197A | | *livH* | | ABC-type branched-chain amino acid transport system, permease protein I |
| HQ2210A | | *narK* | | nitrate/nitrite transporter |
| HQ2218A | | *gufA* | | predicted divalent heavy-metal cations transporter |
| HQ2230A | | *sugE* | | small multidrug export protein |
| HQ2231A | | *-* | | major facilitator superfamily transporter |
| HQ2233A | | *cat* | | probable cationic amino acid transport protein |
| HQ2238A | | *gufA* | | predicted divalent heavy-metal cations transporter |
| HQ2244A | | *cysA* | | ABC-type sulfate/molybdate transport systems, ATP-binding protein |
| HQ2245A | | *cysT* | | ABC-type sulfate/molybdate transport system, permease protein |
| HQ2246A | | *cysU* | | ABC-type sulfate/molybdate transport systems, permease protein |
| HQ2247A | | *-* | | ABC-type transport system periplasmic substrate-binding protein (probable substrate sulfate/molybdate) |
| HQ2252A | | *cadA* | | cadmium-transporting ATPase (EC 3.6.1.-) |
| HQ2285A | | *-* | | major facilitator superfamily transporter |
| HQ2291A | | *oxlT* | | oxalate/formate antiporter |
| HQ2300A | | *argK* | | transport ATPase, component for probable LAO/AO transport systems |
| HQ2303A | | *cbiO* | | ABC-type cobalt transport system, ATP-binding protein |
| HQ2304A | | *cbiQ* | | ABC-type cobalt transport system, permease protein II |
| HQ2305A | | *-* | | cobalamin transport operon protein |
| HQ2306A | | *cbiM* | | ABC-type cobalt transport system, permease protein I |
| HQ2334A | | *nce* | | Na+/Ca2+-antiporter |
| HQ2342A | | *-* | | major facilitator superfamily transporter |
| HQ2351A | | *kchA* | | Kef-type potassium channel protein |
| HQ2370A | | *putP* | | sodium/proline symporter |
| HQ2375A | | *ccdA* | | cytochrome C biogenesis protein/thiol-disulfide transporter |
| HQ2390A | | *-* | | transport protein (probable polysaccharide biosynthesis transport protein) |
| HQ2430A | | *arsA* | | transport ATPase (EC 3.6.3.16) (probable substrate arsenite) |
| HQ2439A | | *-* | | major facilitator superfamily transporter |
| HQ2440A | | *-* | | transporter of the drug/metabolite transporter (DMT) superfamily |
| HQ2450A | | *rbsB* | | ABC-type transport system, substrate-binding protein |
| HQ2451A | | *rbsA* | | ABC-type ribose transport system, ATP-binding protein |
| HQ2452A | | *rbsC1* | | ABC-type ribose transport system, permease protein |
| HQ2453A | | *rbsC2* | | ABC-type ribose transport system, permease protein |
| HQ2475A | | *dppC* | | ABC-type dipeptide/oligopeptide/nickel transport systems, permease protein |
| HQ2476A | | *dppB* | | ABC-type dipeptide/oligopeptide/nickel transport systems, permease protein |
| HQ2477A | | *dppA* | | ABC-type dipeptide/oligopeptide/nickel transport system, substrate-binding protein |
| HQ2478A | | *dppDF* | | ABC-type dipeptide/oligopeptide/nickel transport systems, ATP-binding protein I & II |
| HQ2503A | | *mscS* | | probable mechanosensitive channel |
| HQ2506A | | *norM* | | sodium-driven multidrug efflux pump |
| HQ2521A | | *-* | | major facilitator superfamily transporter (probable quinolone resistance protein) |
| HQ2533A | | *lysE* | | LysE family transporter |
| HQ2542A | | *-* | | probable membrane protein (11 TMHs) |
| HQ2543A | | *sfuC* | | ABC-type spermidine/putrescine/iron transport system, ATP-binding protein |
| HQ2544A | | *sfuB* | | ABC-type iron(III) transport system, permease protein |
| HQ2545A | | *sfuA* | | ABC-type iron(III) transport system, substrate-binding protein |
| HQ2551A | | *lctP* | | L-lactate permease |
| HQ2562A | | *msc* | | probable mechanosensitive ion channel |
| HQ2596A | | *phnE* | | ABC-type phosphate/phosphonate transport system, permease protein |
| HQ2597A | | *phnC* | | ABC-type phosphate/phosphonate transport system, ATPase protein |
| HQ2598A | | *phnD* | | ABC-type phosphate/phosphonate transport system, substrate-binding protein |
| HQ2603A | | *phnK* | | Phosphonates transport ATP-binding protein PhnK |
| HQ2604A | | *phnL* | | Phosphonates transport ATP-binding protein PhnL |
| HQ2609A | | *arsA* | | probable arsenite efflux ATPase (EC 3.6.1.-) |
| HQ2630A | | *ssuA* | | ABC-type nitrate/sulfonate/bicarbonate transport systems, periplasmic component; putative aliphatic sulfonate binding protein |
| HQ2631A | | *ssuC* | | ABC-type nitrate/sulfonate/bicarbonate transport system, permease component; Sulfonate ABC transporter permease protein |
| HQ2632A | | *ssuB* | | ABC-type nitrate/sulfonate/bicarbonate transport system ATPase component; sulfonate ABC transporter ATP binding protein |
| HQ2687A | | *-* | | probable polysaccharide biosynthesis transport protein |
| HQ2725A | | *msc* | | probable mechanosensitive ion channel |
| HQ2730A | | *glnQ* | | ABC-type glutamine/glutamate/polar amino acids transport system, ATP-binding protein |
| HQ2731A | | *glnP* | | ABC-type glutamine/glutamate/polar amino acids transport system, permease protein |
| HQ2732A | | *glnH* | | ABC-type glutamine/glutamate/polar amino acids transport system, substrate-binding protein |
| HQ2738A | | *rhaT* | | DMT superfamily drug/metabolite transporter |
| HQ2754A | | *livJ* | | ABC-type branched-chain amino acid transport system, substrate-binding protein |
| HQ2755A | | *livH* | | ABC-type branched-chain amino acid transport system, permease protein I |
| HQ2756A | | *livM* | | ABC-type branched-chain amino acid transport systems, permease protein II |
| HQ2757A | | *livG* | | ABC-type branched-chain amino acid transport systems, ATP-binding protein I |
| HQ2758A | | *livF* | | ABC-type branched-chain amino acid transport systems, ATP-binding protein II |
| HQ2805A | | *livF* | | ABC-type branched-chain amino acid transport systems, ATP-binding protein I |
| HQ2806A | | *livG* | | ABC-type branched-chain amino acid transport systems, ATP-binding protein II |
| HQ2807A | | *livM* | | ABC-type branched-chain amino acid transport system, permease protein II |
| HQ2808A | | *livH* | | ABC-type branched-chain amino acid transport system, permease protein I |
| HQ2809A | | *livJ* | | ABC-type branched-chain amino acid transport systems, substrate-binding protein |
| HQ2962A | | *livH* | | ABC-type branched-chain amino acid transport system, permease protein I |
| HQ2963A | | *livM* | | ABC-type branched-chain amino acid transport system, permease protein II |
| HQ2964A | | *livG* | | ABC-type branched-chain amino acid transport systems, ATP-binding protein I |
| HQ2965A | | *livF* | | ABC-type branched-chain amino acid transport systems, ATP-binding protein II |
| HQ2969A | | *livJ* | | ABC-type branched-chain amino acid transport systems, substrate-binding protein |
| HQ3044A | | *-* | | transporter of the drug/metabolite transporter (DMT) superfamily |
| HQ3064A | | *hop* | | halorhodopsin |
| HQ3080A | | *azlC* | | ABC-type transport system permease protein (probable substrates branched-chain amino acids), azaleucine resistance protein |
| HQ3092A | | *-* | | ABC-type transport system, permease protein |
| HQ3093A | | *-* | | ABC-type transport system, ATP-binding protein |
| HQ3102A | | *kefC* | | probable potassium transport protein kefC |
| HQ3143A | | *trkA* | | TrkA-domain protein (NAD-binding protein) |
| HQ3182A | | *livJ* | | ABC-type branched-chain amino acid transport systems, substrate-binding protein |
| HQ3183A | | *livH* | | ABC-type branched-chain amino acid transport system, permease protein I |
| HQ3184A | | *livM* | | ABC-type branched-chain amino acid transport system, permease protein II |
| HQ3185A | | *livG* | | ABC-type branched-chain amino acid transport systems, ATP-binding protein I |
| HQ3186A | | *livF* | | ABC-type branched-chain amino acid transport systems, ATP_binding protein II |
| HQ3206A | | *-* | | cation transporter (probable substrates zinc/cadmium) |
| HQ3227A | | *-* | | predicted transporter |
| HQ3299A | | *livF* | | ABC-type branched-chain amino acid transport systems, ATP-binding protein II |
| HQ3300A | | *livG* | | ABC-type branched-chain amino acid transport systems, AT-binding protein I |
| HQ3301A | | *livH* | | ABC-type branched-chain amino acid transport system, permease protein I |
| HQ3302A | | *livM* | | ABC-type branched-chain amino acid transport system, permease protein II |
| HQ3303A | | *livJ* | | ABC-type branched-chain amino acid transport systems, substrate-binding protein |
| HQ3326A | | *panF* | | sodium/pantothenate symporter |
| HQ3380A | | *potC* | | ABC-type spermidine/putrescine transport system, permease protein II |
| HQ3381A | | *potB* | | ABC-type spermidine/putrescine transport system, permease protein I |
| HQ3382A | | *potA* | | ABC-type spermidine/putrescine transport system, ATP-binding protein |
| HQ3383A | | *potD* | | ABC-type spermidine/putrescine transport system, substrate-binding protein |
| HQ3416A | | *-* | | divalent cation transporter, subunit II, MgtE family protein |
| HQ3417A | | *-* | | divalent cation transporter, subunit I, MgtE family protein |
| HQ3444A | | *amtB* | | ammonium transporter |
| HQ3455A | | *pitA* | | probable phosphate permease |
| HQ3476A | | *lolE* | | ABC-type transport system, permease protein II similar to LolDCE lipoprotein release factor |
| HQ3477A | | *lolC* | | ABC-type transport system, permease protein I similar to LolDCE lipoprotein release factor |
| HQ3478A | | *lolD* | | ABC-type transport system, ATP-binding protein similar to LolDE lipoprotein release factor |
| HQ3533A | | *-* | | ABC-type multidrug/lipid transport system, permease and ATP-binding protein |
| HQ3615A | | *urtE* | | ABC-type urea transport systems, ATP-binding protein II |
| HQ3616A | | *urtD* | | ABC-type urea transport system, ATP-binding protein I |
| HQ3617A | | *urtC* | | ABC-type urea transport system, permease protein II |
| HQ3618A | | *urtB* | | ABC-type urea transport system, permease protein I |
| HQ3619A | | *urtA* | | ABC-type urea transport systems, substrate-binding protein |
| HQ3621A | | *cbiM1* | | ABC-type cobalt transport system, permease protein |
| HQ3622A | | *cbiM2* | | ABC-type cobalt transport system, permease protein |
| HQ3623A | | *cbiQ* | | ABC-type cobalt transport system, permease protein |
| HQ3624A | | *cbiO* | | ABC-type cobalt transport system, ATP binding protein |
| HQ3634A | | *-* | | probable cationic amino acids transporter/stress response protein |
| HQ3635A | | *trkA* | | TrkA-potassium uptake domain protein (NAD-binding protein) |
| HQ3664A | | *bioY* | | biotin synthesis protein |
| HQ3665A | | *cbiO* | | ABC-type transport system ATP-binding protein (probable substrate cobalt) |
| HQ3666A | | *cbiQ* | | ABC-type transport system permease protein (probable substrate cobalt) |
| HQ3689A | | *-* | | probable transporter |
| HQ3696A | | *nosF* | | ABC-type copper transport system, ATP-binding protein |
| HQ3697A | | *nosY* | | ABC-type copper transport system, permease protein |
| HQ3701A | | *-* | | major facilitator superfamily transporter |
| HQ3704A | | *-* | | probable phosphate/sulfate permease |
| **CE** | |  | | **cell envelope** |
| ***code*** | | ***gene*** | | ***protein name*** |
| HQ1081A | | *hmu* | | halomucin |
| HQ1082A | | *capB* | | capsular biosynthesis protein CapB / polyglutamate synthase |
| HQ1167A | | *-* | | glycosyltransferase |
| HQ1193A | | *-* | | probable cell surface glycoprotein |
| HQ1195A | | *gp2* | | glycoprotein gp2 |
| HQ1196A | | *-* | | probable cell surface glycoprotein |
| HQ1197A | | *hmu2* | | cell surface glycoprotein |
| HQ1205A | | *-* | | probable cell surface adhesin |
| HQ1206A | | *csg* | | cell surface glycoprotein precursor |
| HQ1207A | | *csg* | | cell surface glycoprotein precursor |
| HQ1249A | | *-* | | GtrA-like family protein, probable cell surface polysaccharide biosynthesis protein |
| HQ1250A | | *-* | | glycosyl tranferase; dolichyl-phosphate beta-glucosyltransferase (EC 2.4.1.117) |
| HQ1346A | | *csg* | | cell surface glycoprotein precursor |
| HQ1558A | | *-* | | hexosyltransferase; glycosyltransferase |
| HQ1596A | | *-* | | probable hemagglutinin like cellsurface protein |
| HQ2508A | | *-* | | LPS glycosyltransferase |
| HQ2519A | | *-* | | probable TP rich cell surface protein similar to viral TPX |
| HQ2625A | | *-* | | glycosyltransferase homolog |
| HQ2691A | | *-* | | hexosyltransferase; glycosyltransferase |
| HQ2692A | | *-* | | glycosyltransferase |
| HQ2714A | | *-* | | probable peptidoglycan binding protein |
| HQ2849A | | *capA* | | poly-gamma-glutamate biosynthesis/capsule biosynthesis protein |
| HQ2915A | | *-* | | cell surface glycoprotein |
| HQ2984A | | *sqdB* | | UDP-sulfoquinovose synthase |
| HQ3135A | | *-* | | predicted cell surface protein/ lipoprotein - putative microfilarial sheath protein |
| HQ3467A | | *hmu3* | | probable cell surface glycoprotein |
| HQ3468A | | *-* | | probable cell surface glycoprotein |
| HQ3469A | | *-* | | probable cell surface glycoprotein |
| HQ3479A | | *-* | | probable extracellular mucin like protein |
| HQ3503A | | *-* | | glycosyltransferase (probable dolichyl-P-glucose synthetase) |
| HQ3518A | | *neuA* | | acylneuraminate cytidylyltransferase (EC 2.7.7.43) |
| HQ3526A | | *bcbD* | | capsular protein |
| HQ3532A | | *-* | | hexosyltransferase; glycosyltransferase |
| HQ3539A | | *-* | | hexosyltransferase; glycosyltransferase |
| HQ3544A | | *-* | | hexosyltransferase; glycosyltransferase |
| HQ3547A | | *-* | | hexosyltransferase; glycosyltransferase |
| HQ3549A | | *-* | | hexosyltransferase; glycosyltransferase |
| HQ3601A | | *cbp* | | cell surface calcium-binding acidic-repeat protein |
| HQ3661A | | *csg* | | probable cell surface glycoprotein precursor |
| HQ4007A | | *csg* | | cell surface glycoprotein precursor |
| **CP** | |  | | **cellular processes** |
| ***code*** | | ***gene*** | | ***protein name*** |
| HQ1001A | | *cdc6_1* | | cell division control protein cdc6 homolog |
| HQ1074A | | *pepB* | | aminopeptidase (similar to leucyl aminopeptidase (EC 3.4.11.1), aminopeptidase T) |
| HQ1110A | | *pepF* | | oligoendopeptidase (EC 3.4.24.-) |
| HQ1163A | | *lon* | | endopeptidase La (EC 3.4.21.53) |
| HQ1243A | | *ftsZ* | | cell division protein ftsZ |
| HQ1268A | | *csp* | | probable cold shock protein |
| HQ1341A | | *gcp* | | O-sialoglycoprotein endopeptidase (EC 3.4.24.57) (glycoprotease)/ serine/threonine protein kinase (EC 2.7.1.-) |
| HQ1359A | | *pepB* | | aminopeptidase (EC 3.4.11.-) (similar to leucyl aminopeptidase, aminopeptidase T) |
| HQ1363A | | *pepQ* | | aminopeptidase; probable Xaa-Pro aminopeptidase (EC 3.4.11.9) |
| HQ1367A | | *cxp* | | predicted carboxypeptidase (EC 3.4.17.-) |
| HQ1388A | | *csp* | | probable cold shock protein |
| HQ1415A | | *ftsZ* | | cell division protein ftsZ |
| HQ1436A | | *hstA* | | archaeal histone |
| HQ1437A | | *aup* | | histone deacetylase |
| HQ1489A | | *dpg* | | probable dolichyl-phosphate beta-glucosyltransferase (EC 2.4.1.117) (only N-terminal homology) |
| HQ1516A | | *psmA1* | | proteasome alpha-1subunit |
| HQ1544A | | *pan2* | | proteasome-activating nucleotidase 2 |
| HQ1650A | | *-* | | CBS domain protein / probable chromosome partitioning protein |
| HQ1731A | | *cdc6_* | | cell division control protein cdc6 homolog |
| HQ1781A | | *gvpC* | | gas-vesicle protein gvpC |
| HQ1782A | | *gvpA* | | gas-vesicle protein gvpA |
| HQ1866A | | *csp* | | probable cold shock protein |
| HQ2454A | | *psmA2* | | proteasome alpha-2 subunit |
| HQ2661A | | *psmB* | | proteasome beta subunit |
| HQ2952A | | *cdc6_* | | cell division control protein cdc6 homolog (fragment probably interupted by IS element) |
| HQ2959A | | *cdc6_* | | cell division control protein cdc6 homolog |
| HQ3115A | | *pan1* | | proteasome-activating nucleotidase 1 |
| HQ3147A | | *ftsZ* | | cell division protein ftsZ |
| HQ3268A | | *cdc6_* | | cell division control protein cdc6 homolog |
| HQ3269A | | *cdc6_* | | cell division control protein cdc6 homolog |
| HQ3353A | | *pepQ* | | Xaa-Pro aminopeptidase (EC 3.4.11.9) |
| HQ3641A | | *pepB* | | aminopeptidase, probable leucyl aminopeptidase (EC 3.4.11.1) |
| HQ3680A | | *cdc6_* | | cell division control protein cdc6 homolog |
| **ENV** | |  | | **environmental information processing** |
| **SIG** | |  | | **signal transduction** |
| ***code*** | | ***gene*** | | ***protein name*** |
| HQ1036A | | *cstA* | | carbon starvation protein A |
| HQ1063A | | *usp* | | homolog to stress response protein |
| HQ1065A | | *usp* | | homolog to stress response protein |
| HQ1066A | | *usp* | | probable stress response protein |
| HQ1073A | | *-* | | signal-transducing histidine kinase; response regulator; PAS/PAC domain protein |
| HQ1216A | | *-* | | probable stress response protein |
| HQ1280A | | *-* | | signal-transducing histidine kinase; response regulator; PAS/PAC domain protein |
| HQ1373A | | *-* | | probable stress response protein |
| HQ1420A | | *pkn* | | RIO-like serine/threonine protein kinase (EC 2.7.1.37) |
| HQ1472A | | *-* | | halobacterial transducer protein homolog |
| HQ1613A | | *usp* | | homolog to stress response protein |
| HQ1739A | | *boa* | | bacterio-opsin activator-like transcription regulator |
| HQ1763A | | *usp* | | probable stress response protein |
| HQ1910A | | *-* | | probable signal-transducing histidine kinase; PAS/PAC domain protein |
| HQ1914A | | *ski* | | probable two-component system sensor kinase |
| HQ1946A | | *-* | | probable response regulator / probable transcription regulator |
| HQ1957A | | *usp* | | probable stress response protein |
| HQ1960A | | *usp* | | probable stress response protein |
| HQ2022A | | *uspA* | | universal stress protein |
| HQ2053A | | *-* | | signal-transducing histidine kinase; response regulator; PAS/PAC domain protein |
| HQ2054A | | *-* | | signal-transducing histidine kinase |
| HQ2141A | | *-* | | signal-transducing histidine kinase; response regulator; PAS/PAC domain protein |
| HQ2346A | | *usp* | | probable stress response protein |
| HQ2372A | | *uspA* | | probable stress response protein |
| HQ2594A | | *-* | | phosphate transport system regulator related protein |
| HQ2709A | | *ptsI* | | Phosphoenolpyruvate-protein phosphotransferase (EC 2.7.3.9) (Phosphotransferase system, enzyme I) |
| HQ2749A | | *-* | | signal-transducing histidine kinase; PAS/PAC domain protein |
| HQ2750A | | *bat* | | Bacterio-opsin like activator / GAF, PAS/PAC domain protein |
| HQ2751A | | *-* | | probable response regulator / probable transcription regulator |
| HQ2796A | | *uspA* | | probable stress response protein |
| HQ2814A | | *-* | | signal-transducing histidine kinase |
| HQ2864A | | *-* | | probable stress response protein |
| HQ3039A | | *-* | | probable stress response protein |
| HQ3723A | | *uspA* | | probable stress response protein |
| HQ4020A | | *-* | | signal-transducing histidine kinase; response regulator; PAS/PAC domain protein |
| **REG** | |  | | **gene regulation** |
| ***code*** | | ***gene*** | | ***protein name*** |
| HQ1041A | | *-* | | transcription regulator |
| HQ1084A | | *bat* | | bacterioopsin activator |
| HQ1105A | | *-* | | probable DNA binding protein |
| HQ1118A | | *lrp* | | transcription regulator |
| HQ1169A | | *-* | | probable transcription regulator, ArsR like |
| HQ1213A | | *-* | | probable transcription regulator |
| HQ1230A | | *padR* | | transcription regulator |
| HQ1315A | | *asnC* | | transcriptional regulator containig TrkA-domain protein (NAD-binding protein) |
| HQ1370A | | *-* | | probable transcription regulator, arsR family |
| HQ1414A | | *-* | | probable transcription regulator |
| HQ1517A | | *-* | | transcription regulator / transcriptional repressor CinR |
| HQ1541A | | *-* | | probable transcription regulator, ArsR family |
| HQ1563A | | *sirR* | | transcription regulator SirR |
| HQ1568A | | *-* | | transcription regulator |
| HQ1586A | | *dpsA* | | DNA-binding protein, ferretin-like domain protein |
| HQ1710A | | *sirR* | | transcription regulator SirR |
| HQ1724A | | *-* | | transcription regulator |
| HQ1737A | | *phoU* | | phosphate transport operon regulator |
| HQ1740A | | *-* | | probable transcription regulator |
| HQ1742A | | *-* | | probable transcription regulator |
| HQ1787A | | *bat* | | bacterioopsin activator/sensor histidine kinase |
| HQ1811A | | *lrp* | | transcription regulator |
| HQ1861A | | *-* | | predicted DNA binding protein, probable transcriptional regulator |
| HQ1863A | | *-* | | DNA binding protein, bacterio-opsin activator-like |
| HQ1864A | | *brp* | | bop gene expression regulator |
| HQ1932A | | *-* | | probable light- and/or oxygen-sensing histidine kinase |
| HQ1937A | | *-* | | transcription regulator |
| HQ1949A | | *-* | | probable transcription regulator,ArsR family |
| HQ1993A | | *-* | | transcription regulator |
| HQ2155A | | *-* | | transcriptional regulator |
| HQ2190A | | *-* | | probable transcriptional regulator |
| HQ2221A | | *nikR* | | Putative nickel responsive regulator |
| HQ2225A | | *-* | | probable transcription regulator |
| HQ2369A | | *tenA* | | transcription regulator |
| HQ2373A | | *-* | | regulatory protein PrrC |
| HQ2407A | | *padR* | | transcription regulator |
| HQ2434A | | *-* | | transcription regulator |
| HQ2593A | | *phoU* | | phosphate uptake regulator / ABC transport system regulatory protein |
| HQ2607A | | *arsR* | | arsenical resistance operon repressor (transcription regulator) |
| HQ2608A | | *arsD* | | arsenical resistance operon trans-acting repressor ArsD (transcriptional regulator) |
| HQ2612A | | *arsR* | | transcriptional regulator, ArsR family |
| HQ2619A | | *lrp* | | transcription regulator |
| HQ2649A | | *-* | | probable transcriptional regulator |
| HQ2746A | | *phoU* | | probable transport system regulatory protein |
| HQ2773A | | *lrp* | | transcription regulator |
| HQ2869A | | *-* | | probable transcriptional regulator |
| HQ2874A | | *-* | | probable transcriptional regulator |
| HQ2987A | | *-* | | transcriptional regulator (probable sugar-specific) |
| HQ3029A | | *pspA* | | probable stress response protein |
| HQ3058A | | *-* | | probable transcriptional regulator ( HTH-type) |
| HQ3063A | | *lrp* | | transcription regulator |
| HQ3236A | | *-* | | conserved riboflavin biosynthetic operon protein; probable transtriptional regulator |
| HQ3307A | | *phoU* | | ABC transport system regulatory protein |
| HQ3308A | | *phoU* | | ABC transport system regulatory protein |
| HQ3354A | | *lrp* | | transcription regulator |
| HQ3361A | | *-* | | transcriptional regulator |
| HQ3491A | | *-* | | transcription regulator |
| HQ3496A | | *-* | | transcriptional regulator |
| HQ3588A | | *abrB* | | transcription regulator |
| HQ3620A | | *nikR* | | transcription regulator; probably nickel responsive regulator |
| HQ3642A | | *lrp* | | transcription regulator AsnC family |
| HQ3714A | | *argX* | | probable arginine biosynthesis regulator (lysine biosynthesis regulator lysX homolog) |
| GIP | |  | | genetic information processing |
| TL | |  | | translation |
| code | | gene | | protein name |
| HQ1048A | | trpS | | tryptophan--tRNA ligase (EC 6.1.1.2) |
| HQ1049A | | pheS | | phenylalanine--tRNA ligase (EC 6.1.1.20) alpha chain |
| HQ1050A | | pheY | | phenylalanine--tRNA ligase (EC 6.1.1.20) beta chain |
| HQ1051A | | valS | | valine--tRNA ligase (EC 6.1.1.9) |
| HQ1070A | | rps6R | | ribosomal protein S6.eR |
| HQ1097A | | lysS | | lysine--tRNA ligase (EC 6.1.1.6) |
| HQ1104A | | rps19R | | ribosomal protein S19.eR |
| HQ1107A | | hisS | | histidine--tRNA ligase (EC 6.1.1.21) |
| HQ1253A | | rpl36aR | | ribosomal protein L36a.eR (HLA) |
| HQ1254A | | rps27R | | ribosomal protein S27.eR |
| HQ1255A | | tif2a | | translation initiation factor aIF-2 alpha subunit |
| HQ1266A | | aatB | | aspartyl-tRNA(Asn) amidotransferase (EC 6.3.5.-) subunit B |
| HQ1270A | | aspS | | aspartate--tRNA ligase (EC 6.1.1.12) |
| HQ1283A | | rpl37aR | | ribosomal protein L37a.eR |
| HQ1324A | | rpl40R | | ribosomal protein L40.eR |
| HQ1335A | | tif2g | | translation initiation factor aIF-2 gamma subunit |
| HQ1340A | | rps24R | | ribosomal protein S24.eR |
| HQ1391A | | rpl10R | | ribosomal protein L10.eR |
| HQ1424A | | rpl15R | | ribosomal protein L15.eR |
| HQ1466A | | psp | | ribonuclease, PSP-type; translation intitiation inhibition protein |
| HQ1504A | | gar1 | | probable RNA-binding protein involved in rRNA processing |
| HQ1572A | | metS | | methionine--tRNA ligase (EC 6.1.1.10) |
| HQ1654A | | cysS | | cysteine--tRNA ligase (EC 6.1.1.16) |
| HQ1687A | | aatA | | aspartyl-tRNA(Asn) amidotransferase (EC 6.3.5.-) subunit A |
| HQ1688A | | aatC | | aspartyl-tRNA(Asn) amidotransferase (EC 6.3.5.-) subunit C |
| HQ1715A | | proS | | proline--tRNA ligase (EC 6.1.1.15) |
| HQ1727A | | tif1A2 | | translation initiation factor aIF-1A II (bacterial-type IF1) |
| HQ1868A | | - | | probable RNA methylase |
| HQ2317A | | leuS | | leucine--tRNA ligase (EC 6.1.1.4) |
| HQ2358A | | rps3aR | | ribosomal protein S3a.eR |
| HQ2361A | | rps15 | | ribosomal protein S15 |
| HQ2468A | | gatE | | glutamyl-tRNA (Gln) amidotransferase (EC 6.3.5.-) subunit E |
| HQ2573A | | tif2b | | translation initiation factor aIF-2 beta subunit |
| HQ2579A | | thrS | | threonine--tRNA ligase (EC 6.1.1.3) |
| HQ2590A | | dph2 | | probable diphthamide synthase subunit DPH2 |
| HQ2637A | | sun | | tRNA and rRNA cytosine-C5-methylases |
| HQ2671A | | ileS | | isoleucine--tRNA ligase (EC 6.1.1.5) |
| HQ2713A | | tif2Ba | | translation initiation factor 2B alpha chain |
| HQ2776A | | gatD | | glutamyl-tRNA(Gln) amidotransferase (EC 6.3.5.-) subunit D |
| HQ2800A | | - | | translation elongation factor aEF-1 alpha subunit homolog |
| **GIP** |  | | **genetic information processing** | |
| **TL** |  | | **translation** | |
| ***Code*** | ***gene*** | | ***protein name*** | |
| HQ1048A | *trpS* | | tryptophan--tRNA ligase (EC 6.1.1.2) | |
| HQ1049A | *pheS* | | phenylalanine--tRNA ligase (EC 6.1.1.20) alpha chain | |
| HQ1050A | *pheY* | | phenylalanine--tRNA ligase (EC 6.1.1.20) beta chain | |
| HQ1051A | *valS* | | valine--tRNA ligase (EC 6.1.1.9) | |
| HQ1070A | *rps6R* | | ribosomal protein S6.eR | |
| HQ1097A | *lysS* | | lysine--tRNA ligase (EC 6.1.1.6) | |
| HQ1104A | *rps19R* | | ribosomal protein S19.eR | |
| HQ1107A | *hisS* | | histidine--tRNA ligase (EC 6.1.1.21) | |
| HQ1253A | *rpl36aR* | | ribosomal protein L36a.eR (HLA) | |
| HQ1254A | *rps27R* | | ribosomal protein S27.eR | |
| HQ1255A | *tif2a* | | translation initiation factor aIF-2 alpha subunit | |
| HQ1266A | *aatB* | | aspartyl-tRNA(Asn) amidotransferase (EC 6.3.5.-) subunit B | |
| HQ1270A | *aspS* | | aspartate--tRNA ligase (EC 6.1.1.12) | |
| HQ1283A | *rpl37aR* | | ribosomal protein L37a.eR | |
| HQ1324A | *rpl40R* | | ribosomal protein L40.eR | |
| HQ1335A | *tif2g* | | translation initiation factor aIF-2 gamma subunit | |
| HQ1340A | *rps24R* | | ribosomal protein S24.eR | |
| HQ1391A | *rpl10R* | | ribosomal protein L10.eR | |
| HQ1424A | *rpl15R* | | ribosomal protein L15.eR | |
| HQ1466A | *psp* | | ribonuclease, PSP-type; translation intitiation inhibition protein | |
| HQ1504A | *gar1* | | probable RNA-binding protein involved in rRNA processing | |
| HQ1572A | *metS* | | methionine--tRNA ligase (EC 6.1.1.10) | |
| HQ1654A | *cysS* | | cysteine--tRNA ligase (EC 6.1.1.16) | |
| HQ1687A | *aatA* | | aspartyl-tRNA(Asn) amidotransferase (EC 6.3.5.-) subunit A | |
| HQ1688A | *aatC* | | aspartyl-tRNA(Asn) amidotransferase (EC 6.3.5.-) subunit C | |
| HQ1715A | *proS* | | proline--tRNA ligase (EC 6.1.1.15) | |
| HQ1727A | *tif1A2* | | translation initiation factor aIF-1A II (bacterial-type IF1) | |
| HQ1868A | *-* | | probable RNA methylase | |
| HQ2317A | *leuS* | | leucine--tRNA ligase (EC 6.1.1.4) | |
| HQ2358A | *rps3aR* | | ribosomal protein S3a.eR | |
| HQ2361A | *rps15* | | ribosomal protein S15 | |
| HQ2468A | *gatE* | | glutamyl-tRNA (Gln) amidotransferase (EC 6.3.5.-) subunit E | |
| HQ2573A | *tif2b* | | translation initiation factor aIF-2 beta subunit | |
| HQ2579A | *thrS* | | threonine--tRNA ligase (EC 6.1.1.3) | |
| HQ2590A | *dph2* | | probable diphthamide synthase subunit DPH2 | |
| HQ2637A | *sun* | | tRNA and rRNA cytosine-C5-methylases | |
| HQ2671A | *ileS* | | isoleucine--tRNA ligase (EC 6.1.1.5) | |
| HQ2713A | *tif2Ba* | | translation initiation factor 2B alpha chain | |
| HQ2776A | *gatD* | | glutamyl-tRNA(Gln) amidotransferase (EC 6.3.5.-) subunit D | |
| HQ2800A | *-* | | translation elongation factor aEF-1 alpha subunit homolog | |
| HQ2820A | *rpl15* | | ribosomal protein L15 | |
| HQ2821A | *rpl30* | | ribosomal protein L30 | |
| HQ2822A | *rps5* | | ribosomal protein S5 | |
| HQ2823A | *rpl18* | | ribosomal protein L18 | |
| HQ2824A | *rpl19R* | | ribosomal protein L19.eR | |
| HQ2825A | *rpl32R* | | ribosomal protein L32.eR | |
| HQ2826A | *rpl6* | | ribosomal protein L6 | |
| HQ2827A | *rps8* | | ribosomal protein S8 | |
| HQ2828A | *rps14* | | ribosomal protein S14 | |
| HQ2829A | *rpl5* | | ribosomal protein L5 | |
| HQ2830A | *rps4R* | | ribosomal protein S4.eR | |
| HQ2831A | *rpl24* | | ribosomal protein L24 | |
| HQ2832A | *rpl14* | | ribosomal protein L14 | |
| HQ2833A | *rps17* | | ribosomal protein S17 | |
| HQ2834A | *rnpA* | | Ribonuclease P protein component 1 (RNase P component 1) (EC 3.1.26.5) | |
| HQ2835A | *rpl29* | | ribosomal protein L29 | |
| HQ2836A | *rps3* | | ribosomal protein S3 | |
| HQ2837A | *rpl22* | | ribosomal protein L22 | |
| HQ2838A | *rps19* | | ribosomal protein S19 | |
| HQ2839A | *rpl2* | | ribosomal protein L2 | |
| HQ2840A | *rpl23* | | ribosomal protein L23 | |
| HQ2841A | *rpl4R* | | ribosomal protein L4.eR | |
| HQ2842A | *rpl3* | | ribosomal protein L3 | |
| HQ2850A | *-* | | translation initiation factor aIF-2 beta subunit homolog; predicted RNA-binding protein | |
| HQ2873A | *alaS* | | alanine--tRNA ligase (EC 6.1.1.7) | |
| HQ2883A | *rpl24R* | | ribosomal protein L24.eR | |
| HQ2884A | *rps28R* | | ribosomal protein S28.eR | |
| HQ2885A | *rpl7aR* | | ribosomal protein L7a.eR/HS6 | |
| HQ2888A | *gltS* | | glutamate--tRNA ligase (EC 6.1.1.17) | |
| HQ2892A | *rpl37R* | | ribosomal protein L37.eR | |
| HQ2896A | *rpl21R* | | ribosomal protein L21.eR | |
| HQ2898A | *tef1b* | | translation elongation factor aEF-1 beta subunit | |
| HQ2901A | *rpl12* | | ribosomal protein L12 | |
| HQ2902A | *rpl10* | | ribosomal protein L10 | |
| HQ2903A | *rpl1* | | ribosomal protein L1 | |
| HQ2922A | *rpl11* | | ribosomal protein L11 | |
| HQ2934A | *rps2* | | ribosomal protein S2 | |
| HQ2938A | *rps9* | | ribosomal protein S9 | |
| HQ2939A | *rpl13* | | ribosomal protein L13 | |
| HQ2940A | *rpl18R* | | ribosomal protein L18.eR | |
| HQ2942A | *rps11* | | ribosomal protein S11 | |
| HQ2943A | *rps4* | | ribosomal protein S4 | |
| HQ2944A | *rps13* | | ribosomal protein S13 | |
| HQ3013A | *-* | | tRNA-dihydrouridine synthase | |
| HQ3016A | *rps17R* | | ribosomal protein S17.eR | |
| HQ3051A | *-* | | probable ribosomal RNA assembly protein | |
| HQ3074A | *tif5B* | | translation initiation factor aIF-5B (bacterial-type IF2) | |
| HQ3138A | *serS* | | serine--tRNA ligase (EC 6.1.1.11) | |
| HQ3162A | *tif1* | | translation initiation factor aIF-1 I (SUI1 protein, bacterial-type IF3) | |
| HQ3178A | *-* | | probable PUA domain RNA-binding protein | |
| HQ3239A | *argS* | | arginine--tRNA ligase (EC 6.1.1.19) | |
| HQ3241A | *erf1* | | peptide chain release factor eRF-1 | |
| HQ3309A | *rps8R* | | ribosomal protein S8.eR | |
| HQ3348A | *tif5A* | | translation initiation factor aIF-5A | |
| HQ3384A | *rps10* | | ribosomal protein S10 | |
| HQ3385A | *tef1a* | | translation elongation factor aEF-1 alpha subunit | |
| HQ3388A | *tef2* | | translation elongation factor aEF-2 | |
| HQ3390A | *rps7* | | ribosomal protein S7 | |
| HQ3391A | *rps12* | | ribosomal protein S12 | |
| HQ3399A | *tif1A1* | | translation initiation factor aIF-1A I (bacterial-type IF1) | |
| HQ3400A | *tyrS* | | tyrosine--tRNA ligase (EC 6.1.1.1) | |
| HQ3407A | *-* | | probable RNA methylase | |
| HQ3421A | *rplX* | | ribosomal protein HL32 (LX) | |
| HQ3422A | *tif6* | | ribosome anti-association protein (initiation factor aIF-6) | |
| HQ3423A | *rpl31R* | | ribosomal protein L31.eR | |
| HQ3424A | *rpl39R* | | ribosomal protein L39.eR | |
| HQ3435A | *-* | | posible NMD protein affecting ribosome stability and mRNA decay | |
| HQ3670A | *alaS* | | alanine--tRNA ligase (EC 6.1.1.7) | |
| HQ3682A | *trmU* | | tRNA methyl transferase (EC 2.1.1.61) | |
| HQ3709A | *glyS* | | glycine--tRNA ligase (EC 6.1.1.14) | |
| **TC** |  | | **transcription** | |
| ***code*** | ***gene*** | | ***protein name*** | |
| HQ1153A | *tfbA* | | transcription initiation factor TFB | |
| HQ1227A | *tfbA* | | transcription initiation factor TFB | |
| HQ1281A | *truD* | | probable tRNA pseudouridine synthase D (Pseudouridylate synthase) (Uracil hydrolyase) (EC 4.2.1.70) | |
| HQ1284A | *rpoP* | | DNA-directed RNA polymerase (EC 2.7.7.6) subunit P | |
| HQ1337A | *rpoE1* | | DNA-directed RNA polymerase (EC 2.7.7.6) subunit E' | |
| HQ1338A | *rpoE2* | | DNA-directed RNA polymerase (EC 2.7.7.6) subunit E'' | |
| HQ1625A | *tfbA* | | transcription initiation factor TFB | |
| HQ1689A | *tfbA* | | transcription initiation factor TFB | |
| HQ1696A | *rpoL* | | DNA-directed RNA polymerase (EC 2.7.7.6) subunit L | |
| HQ1867A | *tfbA* | | transcription initiation factor TFB | |
| HQ2396A | *rpoM1* | | DNA-directed RNA polymerase (EC 2.7.7.6) subunit M1 | |
| HQ2491A | *tfeA* | | transcription initiation factor IIE alpha subunit homolog | |
| HQ2527A | *tbpE* | | TATA-binding transcription initiation factor | |
| HQ2564A | *tfbA* | | transcription initiation factor TFB | |
| HQ2571A | *tfbA* | | transcription initiation factor TFB | |
| HQ2726A | *trmB* | | probable sugar-specific transcriptional regulator TrmB | |
| HQ2779A | *-* | | transcription regulator | |
| HQ2895A | *rpoF* | | DNA-directed RNA polymerase (EC 2.7.7.6) subunit F | |
| HQ2936A | *rpoK* | | DNA-directed RNA polymerase (EC 2.7.7.6) subunit K | |
| HQ2937A | *rpoN* | | DNA-directed RNA polymerase (EC 2.7.7.6) subunit N | |
| HQ2941A | *rpoD* | | DNA-directed RNA polymerase (EC 2.7.7.6) subunit D | |
| HQ3190A | *rpoM2* | | DNA-directed RNA polymerase (EC 2.7.7.6) subunit M2 | |
| HQ3392A | *nusA* | | transcription elongation factor nusA | |
| HQ3393A | *rpoA2* | | DNA-directed RNA polymerase (EC 2.7.7.6) subunit A'' (subunit C) | |
| HQ3394A | *rpoA* | | DNA-directed RNA polymerase (EC 2.7.7.6) subunit A' (subunit A) | |
| HQ3395A | *rpoB1* | | DNA-directed RNA polymerase (EC 2.7.7.6) subunit B' | |
| HQ3396A | *rpoB2* | | DNA-directed RNA polymerase (EC 2.7.7.6) subunit B'' | |
| HQ3397A | *rpoH* | | DNA-directed RNA polymerase (EC 2.7.7.6) subunit H | |
| HQ3408A | *tfbA* | | transcription initiation factor TFB | |
| HQ3410A | *tbpE* | | TATA-binding transcription initiation factor | |
| HQ3654A | *tfbA* | | transcription initiation factor TFB | |
| **RRR** |  | | **replication, repair, recombination** | |
| ***code*** | ***gene*** | | ***protein name*** | |
| HQ1003A | *polA1* | | DNA-directed DNA polymerase (EC 2.7.7.7) small subunit (family D) (archaeal DNA polymerase II) | |
| HQ1008A | *mer3* | | ATP-dependent DNA helicase (EC 3.6.1.-) | |
| HQ1045A | *topA* | | DNA topoisomerase I (EC 5.99.1.2) | |
| HQ1046A | *mer3* | | ATP-dependent DNA helicase (EC 3.6.1.-) | |
| HQ1151A | *nthA* | | endonuclease III; DNA-(apurinic or apyrimidinic site) lyase (EC 4.2.99.18) | |
| HQ1160A | *ogt* | | methylated-DNA--protein-cysteine methyltransferase (EC 2.1.1.63) | |
| HQ1220A | *polIVx* | | DNA-directed DNA polymerase (EC 2.7.7.7) (family X) / ycdX protein homolog | |
| HQ1228A | *rnhA* | | ribonuclease H (EC 3.1.26.4) I | |
| HQ1267A | *topA* | | DNA topoisomerase I (EC 5.99.1.2) | |
| HQ1298A | *phr* | | deoxyribodipyrimidine photolyase (EC 4.1.99.3) | |
| HQ1364A | *uvrD* | | DNA helicase (EC 3.6.1.-) II | |
| HQ1380A | *-* | | uracil-DNA glycosylase homolog | |
| HQ1435A | *rpa* | | replication factor A | |
| HQ1445A | *phr* | | deoxyribodipyrimidine photolyase (EC 4.1.99.3) | |
| HQ1456A | *mutL* | | DNA mismatch repair protein MutL | |
| HQ1460A | *mutS1* | | DNA mismatch repair protein MutS1 | |
| HQ1545A | *nthA* | | endonuclease III; DNA-(apurinic or apyrimidinic site) lyase (EC 4.2.99.18) | |
| HQ1581A | *-* | | RecJ like exonuclease | |
| HQ1631A | *mer3* | | ATP-dependent DNA helicase (EC 3.6.1.-) | |
| HQ1700A | *mre11* | | DNA double-strand break repair protein mre11 | |
| HQ1701A | *rad50* | | DNA double-strand break repair rad50 ATPase | |
| HQ1705A | *polB1* | | DNA-directed DNA polymerase (family B) | |
| HQ1797A | *-* | | RecJ-like exonuclease | |
| HQ1852A | *-* | | probable DNA repair and recombination protein radA | |
| HQ1909A | *nuc* | | endonuclease Staphylococcal nuclease-like | |
| HQ1981A | *rad3* | | DNA repair helicase Rad3 (EC 3.6.1.-) | |
| HQ2011A | *uvdE* | | UV DNA damage endonuclease | |
| HQ2032A | *xseAs* | | exonuclease VII small subunit (EC 3.1.11.6) | |
| HQ2033A | *xseAl* | | exodeoxyribonuclease VII large subunit (EC 3.1.11.6) | |
| HQ2075A | *rad* | | predicted ATPase invovled in DNA repair | |
| HQ2077A | *-* | | site-specific DNA-methyltransferase (EC 2.1.1.-) | |
| HQ2078A | *-* | | site-specific DNA-methyltransferase (EC 2.1.1.-) | |
| HQ2079A | *-* | | DNA repair helicase | |
| HQ2080A | *smc* | | chromosome segregation protein | |
| HQ2087A | *-* | | probable helicase family protein | |
| HQ2149A | *uvrD* | | DNA helicase II | |
| HQ2327A | *ligB* | | DNA ligase (ATP) | |
| HQ2405A | *rpa* | | replication factor A | |
| HQ2418A | *rad2* | | flap structure-specific endonuclease / DNA repair protein Rad2 | |
| HQ2432A | *rnhA* | | ribonuclease H I | |
| HQ2463A | *phr* | | deoxyribodipyrimidine photolyase (EC 4.1.99.3) | |
| HQ2481A | *mutY* | | A/G-specific adenine glycosylase | |
| HQ2577A | *ogg* | | DNA N-glycosylase / DNA lyase | |
| HQ2614A | *xerC* | | tyrosine recombinase xerC | |
| HQ2651A | *gyrA* | | DNA gyrase / DNA topoisomerase II subunit A (EC 5.99.1.3) | |
| HQ2652A | *gyrB* | | DNA gyrase / DNA topoisomerase II subunit B (EC 5.99.1.3) | |
| HQ2653A | *top6B* | | DNA topoisomerase VI subunit B (type II DNA topoisomerase) | |
| HQ2654A | *top6A* | | DNA topoisomerase VI subunit A (type II DNA topoisomerase) | |
| HQ2659A | *ligB* | | DNA ligase (ATP) | |
| HQ2719A | *priA* | | DNA primase small subunit | |
| HQ2729A | *rfcB* | | replication factor C large subunit | |
| HQ2752A | *lhr* | | Lhr-like helicase (EC 3.6.1.-) | |
| HQ2912A | *-* | | probable integrase/recombinase | |
| HQ2928A | *lhr* | | Lhr-like helicase (EC 3.6.1.-) | |
| HQ2930A | *dna2* | | DNA2-like DNA replication helicase | |
| HQ3072A | *uvrA* | | excinuclease ABC chain A | |
| HQ3096A | *rnhB* | | ribonuclease HII (EC 3.1.26.4) | |
| HQ3157A | *mutL* | | DNA mismatch repair protein MutL | |
| HQ3158A | *mutS2* | | DNA mismatch repair protein MutS2 | |
| HQ3173A | *radB* | | DNA repair and recombination protein RadB | |
| HQ3267A | *-* | | phage integrase homolog; halovirus HF1/2 protein | |
| HQ3275A | *-* | | probable type II restriction/modification enzyme | |
| HQ3276A | *hepA* | | ATP-dependent helicase HepA | |
| HQ3277A | *-* | | adenine-specific DNA methyltransferase | |
| HQ3328A | *dnaG* | | DNA primase DnaG | |
| HQ3359A | *polIVy* | | DNA-directed DNA polymerase (EC 2.7.7.7) (family Y) (archaeal DNA polymerase IV) | |
| HQ3375A | *nuc* | | endonuclease Staphylococcal nuclease-like | |
| HQ3405A | *rfcC* | | replication factor C small subunit II | |
| HQ3429A | *radA* | | DNA repair and recombination protein RadA | |
| HQ3461A | *polA2* | | DNA-directed DNA polymerase (EC 2.7.7.7) large subunit (family D) (archaeal DNA polymerase II) | |
| HQ3590A | *repH* | | plasmid replication protein repH | |
| HQ3652A | *minD* | | probable cell division inhibitor | |
| HQ3662A | *mcm* | | ATP-dependent DNA helicase (intein-containing) | |
| HQ3671A | *rfcA* | | replication factor C small subunit I | |
| HQ3675A | *-* | | probable nuclease | |
| HQ3681A | *mutS3* | | DNA mismatch repair protein MutS homolog, MutS3 | |
| HQ3686A | *pcn* | | DNA-directed DNA polymerase sliding clamp | |
| HQ3688A | *priB* | | probable DNA primase large subunit | |
| HQ3690A | *hjc* | | Holliday junction resolvase | |
| HQ3694A | *uvrC* | | excinuclease ABC chain C | |
| HQ3698A | *ligA* | | DNA ligase (NAD) | |
| HQ3702A | *recJ* | | single-stranded-DNA-specific exonuclease | |
| HQ3720A | *rad3b* | | DNA repair helicase homolog | |
| HQ3725A | *uvrB* | | excinuclease ABC chain B | |
| HQ4001A | *xerD* | | phage integrase/recombinase xerD | |
| HQ4015A | *hepA* | | superfamily II DNA/RNA helicases, SNF2 family | |
| HQ4019A | *rnhA* | | ribonuclease H I | |
| HQ4022A | *-* | | Putative P4-specific DNA primase (EC 2.7.7.-) | |
| HQ4023A | *-* | | probable phage-like primase | |
| HQ4033A | *-* | | probable membrane associated restriction endonuclease (mrr-tye) | |
| HQ4037A | *tnpR* | | resolvase | |
| **RMT** |  | | **RNA maturation** | |
| ***code*** | ***gene*** | | ***protein name*** | |
| HQ1047A | *endA* | | tRNA-intron endonuclease (EC 3.1.27.9) | |
| HQ1108A | *truA* | | tRNA-pseudouridine synthase (EC 5.4.99.12) | |
| HQ1121A | *epf* | | mRNA cleavage and polyadenylation specificity factor homolog | |
| HQ1438A | *cca* | | tRNA adenylyltransferase (EC 2.7.7.25), CCA-adding | |
| HQ1513A | *rnp3* | | ribonuclease P protein component 3 | |
| HQ1515A | *rnp2* | | ribonuclease P (EC 3.1.26.5) protein component 2 | |
| HQ1716A | *epf2* | | mRNA 3'-end processing factor homolog | |
| HQ1996A | *-* | | Predicted metal-dependent RNase, consists of a metallo-beta-lactamase domain and an RNA-binding KH domain | |
| HQ2015A | *-* | | probable tRNA splicing protein, cyclic phosphodiesterase (EC 3.1.4.-) | |
| HQ2036A | *elaC* | | Ribonuclease Z (RNase Z) (tRNA 3 endonuclease)(EC 3.1.26.11) | |
| HQ2326A | *epf* | | mRNA cleavage and polyadenylation specificity factor homolog | |
| HQ2467A | *-* | | probable tRNA/rRNA methyltransferase | |
| HQ2501A | *rnp4* | | ribonuclease P protein component 4 | |
| HQ2567A | *flpA* | | fibrillarin-like pre-rRNA processing protein | |
| HQ2568A | *nop56* | | probable pre-rRNA-processing protein nop56 | |
| HQ2764A | *truB* | | tRNA-pseudouridine synthase (EC 5.4.99.12) | |
| HQ2878A | *ksgA* | | dimethyladenosine transferase (rRNA methylation) | |
| HQ3646A | *trm1* | | tRNA (guanine-N2-)-methyltransferase (EC 2.1.1.32) | |
| HQ3685A | *ftsJ* | | ribosomal RNA large subunit methyltransferase J (EC 2.1.1.-) | |
| **CHP** |  | | **chaperones** | |
| ***code*** | ***gene*** | | ***protein name*** | |
| HQ1069A | *hsp20* | | hsp20-type chaperone | |
| HQ1166A | *ths* | | thermosome subunit | |
| HQ1177A | *hsp20* | | hsp20 type chaperone | |
| HQ1387A | *thsB* | | thermosome, beta subunit | |
| HQ1393A | *hsp20* | | hsp20 type chaperone | |
| HQ1569A | *dnaJ* | | Chaperone protein DnaJ | |
| HQ1793A | *thiJ* | | probable chaperone | |
| HQ1810A | *hsp20* | | hsp20-type chaperone | |
| HQ2412A | *rspA* | | molecular chaperone P45 | |
| HQ2639A | *grpE* | | dnaJ/dnaK ATPase stimulator grpE | |
| HQ2640A | *dnaK* | | dnaK-type molecular chaperone hsp70 | |
| HQ2641A | *dnaJ* | | chaperone protein DnaJ | |
| HQ2801A | *dnaJ* | | chaperone protein dnaJ | |
| HQ3049A | *thsA* | | thermosome, alpha subunit | |
| HQ3346A | *hsp20* | | hsp20 type chaperone | |
| **MIS** |  | | **miscellaneous** | |
| **ISH** |  | | **ISH-encoded transposases** | |
| ***code*** | ***gene*** | | ***protein name*** | |
| HQ1012A | *-* | | IS1341-type transposase | |
| HQ1316A | *-* | | IS200-type transposase | |
| HQ1353A | *-* | | IS1341-type transposase | |
| HQ1356A | *-* | | IS1341-type transposase | |
| HQ1713A | *-* | | IS1341-type transposase | |
| HQ1744A | *-* | | IS200-type transposase | |
| HQ1859A | *-* | | IS1341-type transposase | |
| HQ1924A | *-* | | IS1341-type transposase | |
| HQ1982A | *-* | | transposase homolog (ISHnew96) | |
| HQ1983A | *-* | | transposase | |
| HQ2030A | *-* | | ISH9-type transposase | |
| HQ2051A | *-* | | IS1341-type transposase | |
| HQ2072A | *-* | | IS1341-type transposase | |
| HQ2154A | *-* | | IS1341-type transposase | |
| HQ2200A | *-* | | probable transposase (ISH8/ISH26) | |
| HQ2319A | *-* | | transposase homolog (ISHnew96) | |
| HQ2419A | *-* | | IS1341-type transposase | |
| HQ2420A | *-* | | IS1341-type transposase | |
| HQ2460A | *-* | | Tn5-like transposase | |
| HQ2482A | *-* | | IS200-type transposase | |
| HQ2485A | *-* | | IS1341-type transposase | |
| HQ2515A | *-* | | IS1341-type transposase homolog | |
| HQ2536A | *-* | | IS1341-type transposase | |
| HQ2541A | *-* | | probable transposase (fragment) | |
| HQ2549A | *-* | | IS1341-type transposase | |
| HQ2613A | *-* | | probable transposase (ISHnew91) | |
| HQ2728A | *-* | | IS1341-type transposase | |
| HQ2954A | *-* | | IS1341-type transposase | |
| HQ3062A | *-* | | IS1341-type transposase | |
| HQ3077A | *-* | | IS1341-type transposase | |
| HQ3233A | *-* | | IS1341-type transposase | |
| HQ3237A | *-* | | IS1341-type transposase | |
| HQ3304A | *-* | | IS1341-type transposase | |
| HQ3337A | *-* | | IS1341-type transposase | |
| HQ3338A | *-* | | IS1341-type transposase | |
| HQ3369A | *-* | | transposase fragment | |
| HQ3370A | *-* | | transposase homolog (ISHnew96) | |
| HQ3378A | *-* | | IS1341-type transposase | |
| HQ3448A | *-* | | transposase | |
| HQ3449A | *-* | | transposase homolog (ISHnew96) | |
| HQ3472A | *-* | | transposase | |
| HQ3484A | *-* | | IS1341-type transposase | |
| HQ3516A | *-* | | ISH3-type transposase (fragment) | |
| HQ3520A | *-* | | probable transposase (ISH5) | |
| HQ3521A | *-* | | probable transposase (ISH5) (fragment) | |
| HQ3528A | *-* | | probable transposase (ISH8/ISH26) | |
| HQ3551A | *-* | | transposase homolog (ISHnew96) | |
| HQ3552A | *-* | | ISH4-type transposase | |
| HQ3553A | *-* | | probable transposase (ISH8/ISH26) | |
| HQ3554A | *-* | | probable transposase (ISH8/ISH26) | |
| HQ3555A | *-* | | probable transposase (ISH8/ISH26) | |
| HQ3557A | *-* | | ISRSO17-transposase | |
| HQ3558A | *-* | | probable transposase (ISH11) | |
| HQ3574A | *-* | | IS1341-type transposase | |
| HQ3632A | *-* | | probable insertion element protein (ISH2) | |
| **MIS** |  | | **miscellaneous** | |
| ***code*** | ***gene*** | | ***protein name*** | |
| HQ1021A | *-* | | probable prepilin signal peptidase | |
| HQ1054A | *-* | | nonhistone chromosomal protein | |
| HQ1080A | *kch* | | kef-type K+ transport systems, predicted NAD-binding component | |
| HQ1085A | *lpdA* | | dihydrolipoamide dehydrogenase (EC 1.8.1.4) | |
| HQ1116A | *nirA* | | ferredoxin--nitrite reductase (EC 1.7.7.1) | |
| HQ1124A | *adh* | | alcohol dehydrogenase (EC 1.1.1.1) | |
| HQ1200A | *sub* | | subtilisin-like serine protease | |
| HQ1208A | *sub* | | subtilisin-like serine protease | |
| HQ1231A | *ipp* | | inorganic pyrophosphatase (EC 3.6.1.1) | |
| HQ1234A | *nfi* | | endonuclease V (EC 3.1.21.7) (deoxyinosine 3'endonuclease) | |
| HQ1264A | *smc* | | chromosome partition protein | |
| HQ1286A | *pfdB* | | prefoldin beta subunit | |
| HQ1319A | *act* | | acyl-CoA thioester hydrolase (EC 3.1.2.-) | |
| HQ1357A | *pimT* | | protein-L-isoaspartate O-methyltransferase (EC 2.1.1.77) | |
| HQ1372A | *-* | | predicted RNase L inhibitor; APTase | |
| HQ1398A | *-* | | probable ribonuclease (G/E family) | |
| HQ1399A | *act* | | acyl-CoA thioester hydrolase (EC 3.1.2.-) | |
| HQ1417A | *nfo* | | endonuclease IV (EC 3.1.21.2) | |
| HQ1418A | *aldH* | | aldehyde dehydrogenase (EC 1.2.1.-) (succinate-semialdehyde dehydrogenase homolog) | |
| HQ1439A | *-* | | probable membrane-bound metal-dependent hydrolase | |
| HQ1462A | *-* | | succinylglutamate desuccinylase / aspartoacylase family protein | |
| HQ1520A | *-* | | predicted electron-transferring-flavoprotein dehydrogenase (EC 1.5.5.1) | |
| HQ1523A | *acyP* | | acylphosphatase (EC 3.6.1.7) | |
| HQ1534A | *-* | | predicted electron-transferring-flavoprotein dehydrogenase (EC 1.5.5.1) | |
| HQ1582A | *-* | | nitroreductase family protein | |
| HQ1592A | *trxB* | | thioredoxin reductase (NADPH) (EC 1.8.1.9) | |
| HQ1601A | *-* | | probable S-adenosylmethionine-dependent methyltransferase (EC 2.1.1.-) | |
| HQ1603A | *dph5* | | diphthine synthase (EC 2.1.1.98) | |
| HQ1605A | *glo* | | lyase/ dioxygenase (probable lactoylglutathione lyase (EC 4.4.1.5), aromatic compounds dioxygenase (EC 1.13.11.-)) | |
| HQ1622A | *dsbG* | | protein-disulfide isomerase | |
| HQ1624A | *-* | | predicted methyltransferase | |
| HQ1652A | *trxB* | | thioredoxin reductase (NADPH) (EC 1.8.1.9) | |
| HQ1722A | *hcpE* | | 2-hydroxyhepta-2,4-diene-1,7-dioate isomerase (EC 5.3.3.-); probable 5-oxopent-3-ene-1,2,5-tricarboxylate decarboxylase (EC 4.1.1.68) | |
| HQ1743A | *ppk* | | polyphosphate kinase | |
| HQ1750A | *moxR* | | MoxR-like ATPase | |
| HQ1752A | *smc* | | chromosome partition protein | |
| HQ1756A | *metF* | | 5,10-methylenetetrahydrofolate reductase (EC 1.5.1.20) | |
| HQ1769A | *psp* | | ribonuclease, PSP-type | |
| HQ1770A | *gvpM* | | gas-vesicle operon protein gvpM | |
| HQ1771A | *gvpL* | | gas-vesicle operon protein gvpL | |
| HQ1772A | *gvpK* | | gas-vesicle operon protein gvpK | |
| HQ1773A | *gvpJ* | | gas-vesicle operon protein gvpJ | |
| HQ1774A | *gvpI* | | gas-vesicle operon protein gvpI | |
| HQ1775A | *gvpH* | | gas-vesicle operon protein gvpH | |
| HQ1776A | *gvpG* | | gas-vesicle operon protein gvpG | |
| HQ1777A | *gvpF* | | gas-vesicle operon protein gvpF | |
| HQ1779A | *gvpO* | | gas-vesicle operon protein gvpO | |
| HQ1780A | *gvpN* | | gas-vesicle operon protein gvpN | |
| HQ1792A | *-* | | probable S-adenosylmethionine-dependent methyltransferase (EC 2.1.1.-) | |
| HQ1798A | *hat* | | histone acetyltransferase homolog | |
| HQ1802A | *pelA* | | cell division protein pelota | |
| HQ1806A | *dsbG* | | protein-disulfide isomerase | |
| HQ1843A | *msrA* | | peptide methionine sulfoxide reductase (S-form specific) | |
| HQ1855A | *degP* | | serine proteinase | |
| HQ1886A | *mtfK1* | | FKBP-type peptidylprolyl isomerase 1 | |
| HQ1929A | *msrA* | | peptide methionine sulfoxide reductase (S-form specific) | |
| HQ1930A | *msrB* | | peptide methionine sulfoxide reductase (R-form specific) | |
| HQ1931A | *cutA* | | probable divalent divalent cation tolerance protein | |
| HQ1939A | *qor* | | NADPH:quinone reductase (EC 1.6.5.5) | |
| HQ1940A | *-* | | predicted crotonobetainyl-CoA:carnitine CoA-transferase (EC 2.8.3.-)/alpha-methylacyl-CoA racemase (EC 5.1.99.4) | |
| HQ1970A | *-* | | site-specific DNA-methyltransferase (EC 2.1.1.-) | |
| HQ1972A | *aldH* | | aldehyde dehydrogenase (EC 1.2.1.-) | |
| HQ1973A | *sfsA* | | DNA-binding protein, stimulates sugar fermentation | |
| HQ1987A | *qor* | | NADPH:quinone reductase (EC 1.6.5.5) | |
| HQ1988A | *-* | | predicted crotonobetainyl-CoA:carnitine CoA-transferase (EC 2.8.3.-)/alpha-methylacyl-CoA racemase (EC 5.1.99.4) | |
| HQ2006A | *-* | | probable S-adenosylmethionine-dependent methyltransferase (EC 2.1.1.-) | |
| HQ2014A | *ywaD* | | probable peptidase (EC 3.4.11.-) | |
| HQ2031A | *amyA* | | alpha amylase (EC 3.2.1.-) | |
| HQ2038A | *cysA* | | thiosulfate sulfurtransferase (EC 2.8.1.1) | |
| HQ2040A | *sppA* | | probable signal peptide peptidase | |
| HQ2045A | *-* | | probable phosphoesterase | |
| HQ2046A | *cynT* | | carbonic anhydrase | |
| HQ2047A | *trzA* | | Atrazine chlorohydrolase (EC 3.8.1.8) | |
| HQ2048A | *trzA* | | probable N-ethylammeline chlorohydrolase (S-triazine hydrolase ) | |
| HQ2098A | *-* | | probable integrase (PhiCh1 Int1-like) | |
| HQ2191A | *-* | | acetamidase/formamidase family protein | |
| HQ2211A | *narB* | | nitrate reductase, catalytic subunit (EC 1.7.99.4) | |
| HQ2212A | *nirA* | | ferredoxin--nitrite reductase (EC 1.7.7.1) | |
| HQ2213A | *narB* | | nitrate reductase, catalytic subunit (EC 1.7.99.4) | |
| HQ2259A | *moxR* | | MoxR-like ATPase | |
| HQ2265A | *-* | | possible pyridoxamine 5'-phosphate oxidase | |
| HQ2266A | *-* | | probable periplasmic serine proteinase (fragment) | |
| HQ2267A | *-* | | probable periplasmic serine proteinase (fragment) | |
| HQ2324A | *msrA* | | peptide methionine sulfoxide reductase (S-form specific) | |
| HQ2332A | *-* | | probable S-adenosylmethionine-dependent methyltransferase (EC 2.1.1.-) | |
| HQ2374A | *trxA* | | thioredoxin | |
| HQ2428A | *aslA* | | arylsulfatase (EC 3.1.6.1) | |
| HQ2448A | *oxr* | | probable oxidoreductase (EC 1.1.1.-) | |
| HQ2461A | *sod* | | superoxide dismutase (EC 1.15.1.1) | |
| HQ2473A | *bolA* | | probable BolA-like protein | |
| HQ2497A | *-* | | glycine-rich protein | |
| HQ2548A | *-* | | probable endonuclease associated protein | |
| HQ2581A | *-* | | predicted NAD-binding protein (probable Kef-type transporter subunit) | |
| HQ2585A | *cyaB* | | adenylate cyclase (EC 4.6.1.1) | |
| HQ2586A | *mtfK2* | | FKBP-type peptidylprolyl isomerase 2 | |
| HQ2599A | *phnG* | | Phosphonate metabolism protein PhnG | |
| HQ2600A | *phnH* | | Phosphonate metabolism protein PhnH | |
| HQ2601A | *phnI* | | Phosphonate metabolism protein PhnI | |
| HQ2602A | *phnJ* | | Phosphonate metabolism protein PhnJ | |
| HQ2605A | *phnM* | | Phosphonate metabolism protein PhnM | |
| HQ2667A | *-* | | membrane metallopeptidase | |
| HQ2668A | *traB* | | probable plasmid transfer protein | |
| HQ2681A | *stt3* | | transmembrane oligosaccharyl transferase | |
| HQ2690A | *aslA* | | arylsulfatase (EC 3.1.6.1) | |
| HQ2708A | *hpr* | | phosphocarrier protein Hpr | |
| HQ2735A | *-* | | probable metallopeptidase | |
| HQ2759A | *-* | | probable C2H2 zinc finger DNA-binding protein | |
| HQ2787A | *sppA* | | Protease IV; endopeptidase IV; probable signal peptide peptidase (EC 3.4.21.-) | |
| HQ2788A | *-* | | probable phosphoglycolate phosphatase (EC 3.1.3.18) | |
| HQ2852A | *dhs* | | deoxyhypusine synthase (EC 2.5.1.46) | |
| HQ2879A | *hemK* | | protein methyltransferase | |
| HQ2921A | *msrB* | | peptide methionine sulfoxide reductase (R-form specific) | |
| HQ3002A | *prkA* | | predicted serine protein kinase (EC 2.7.1.37) | |
| HQ3003A | *prkA* | | predicted serine protein kinase (EC 2.7.1.37) | |
| HQ3052A | *crcB* | | protein crcB homolog | |
| HQ3053A | *crcB* | | protein crcB homolog | |
| HQ3059A | *grx* | | probable glutaredoxin | |
| HQ3066A | *-* | | probable metalloprotease (EC 3.4.24.-)/ CBS domain protein | |
| HQ3091A | *sppA* | | Protease IV; endopeptidase IV; probable signal peptide peptidase (EC 3.4.21.-) | |
| HQ3113A | *-* | | predicted electron-transferring-flavoprotein dehydrogenase (EC 1.5.5.1) / geranylgeranyl hydrogenase homolog | |
| HQ3123A | *ppiA* | | peptidylprolyl isomerase (EC 5.2.1.8) | |
| HQ3159A | *moxR* | | MoxR-like ATPase | |
| HQ3170A | *arsC* | | probable arsenate reductase (glutaredoxin) (EC 1.20.4.1); probable protein-tyrosine-phosphatase (EC 3.1.3.48) | |
| HQ3187A | *fdh* | | formaldehyde dehydrogenase (EC 1.2.1.46) | |
| HQ3203A | *aslA* | | arylsulfatase (EC 3.1.6.1) | |
| HQ3207A | *hit* | | probable histidine triad (HIT) hydrolase (probable bis(5'-nucleosyl)-tetraphosphatase (asymmetrical) (EC 3.6.1.17)) | |
| HQ3208A | *map* | | methionyl aminopeptidase (EC 3.4.11.18) | |
| HQ3211A | *dsbA* | | thioredoxin; DsbA oxidoreductase | |
| HQ3221A | *grx* | | probable glutaredoxin | |
| HQ3251A | *menG* | | menaquinone biosynthesis methyltransferase homolog | |
| HQ3260A | *tfx* | | DNA-binding protein | |
| HQ3343A | *pgp* | | probable phosphatase (EC 3.1.3.-) (phosphoglycolate phosphatase homolog) | |
| HQ3350A | *surE* | | acid phosphatase (EC 3.1.3.2) | |
| HQ3367A | *hcpE* | | 2-hydroxyhepta-2,4-diene-1,7-dioate isomerase (EC 5.3.3.-); probable 5-oxopent-3-ene-1,2,5-tricarboxylate decarboxylase (EC 4.1.1.68) | |
| HQ3398A | *-* | | predicted RIO-like serine/threonine protein kinase II | |
| HQ3413A | *trzA* | | probable chlorohydrolase | |
| HQ3415A | *-* | | sulfatase (EC 3.1.6.-) | |
| HQ3420A | *pfdA* | | prefoldin alpha subunit | |
| HQ3464A | *aslA* | | probable arylsulfatase (EC 3.1.6.1); probable choline-sulfatase (EC 3.1.6.6) | |
| HQ3474A | *moxR* | | MoxR-like ATPase (methanol dehydrogenase regulatory protein) | |
| HQ3524A | *-* | | protein transglucosylase | |
| HQ3529A | *-* | | probable sulfatase | |
| HQ3540A | *aslA* | | probable arylsulfatase (EC 3.1.6.1) | |
| HQ3543A | *-* | | arylsulfatase (EC 3.1.6.1) | |
| HQ3545A | *-* | | probable arylsulfatase A-like | |
| HQ3556A | *-* | | probable sulfatase | |
| HQ3559A | *-* | | probable sulfatase (fragment) | |
| HQ3560A | *-* | | probable sulfatase (fragment) | |
| HQ3586A | *-* | | probable integrase (PhiCh1 Int1-like) | |
| HQ3636A | *arsC* | | probable arsenate reductase (glutaredoxin) (EC 1.20.4.1); probable protein-tyrosine-phosphatase (EC 3.1.3.48) | |
| HQ3648A | *adh* | | alcohol dehydrogenase, NAD dependent (EC 1.1.1.1) | |
| HQ3651A | *hit* | | probable histidine triad (HIT) hydrolase | |
| HQ3669A | *est* | | 3-oxoadipate enol-lactonase | |
| HQ3676A | *surE* | | acid phosphatase (EC 3.1.3.2) | |
| HQ3692A | *mtfK1* | | FKBP-type peptidylprolyl isomerase 1 | |
| HQ3706A | *grx* | | probable glutaredoxin | |
| HQ3708A | *-* | | probable DNA binding protein; probable translation factor | |
| HQ3721A | *cyc* | | unspecific monooxygenase (EC 1.14.14.1) (cytochrome P450) | |
| HQ3729A | *cysA* | | thiosulfate sulfurtransferase (EC 2.8.1.1) | |
| HQ3730A | *cysA* | | thiosulfate sulfurtransferase (EC 2.8.1.1) | |
| HQ4014A | *-* | | type II restriction enzyme, methylase subunits | |
| HQ4038A | *-* | | SCP-like extracellular protein | |
| **GEN** |  | | **general function** | |
| ***code*** | ***gene*** | | ***protein name*** | |
| HQ1011A | *aaa3* | | AAA-type ATPase (transitional ATPase homolog) | |
| HQ1019A | *brp* | | brp-like protein | |
| HQ1020A | *-* | | probable nuclease domain protein/ probable ATP-dependent RNA helicase | |
| HQ1064A | *-* | | protein N-acetyltransferase homolog | |
| HQ1093A | *-* | | predicted oxidoreductase, aldo/keto reductase family | |
| HQ1095A | *-* | | probable metalloprotease/metallo peptidase | |
| HQ1101A | *-* | | probable membrane associated metalloprotease | |
| HQ1123A | *glo* | | lyase/ dioxygenase (probable lactoylglutathione lyase (EC 4.4.1.5), aromatic compounds dioxygenase (EC 1.13.11.-)) | |
| HQ1135A | *-* | | sugar epimerase/dehydratase homolog | |
| HQ1146A | *-* | | 3-beta hydroxysteroid dehydrogenase/isomerase family | |
| HQ1161A | *blp* | | bacterioopsin-linked protein blp | |
| HQ1165A | *-* | | conserved nad operon protein | |
| HQ1168A | *-* | | S-adenosylmethionine-dependent methyltransferase homolog (EC 2.1.1.-) | |
| HQ1170A | *-* | | probable hydrolase | |
| HQ1173A | *-* | | predicted Fe-S oxidoreductase | |
| HQ1180A | *-* | | glycosyltransferase homolog | |
| HQ1182A | *-* | | aldo/keto reductase family protein | |
| HQ1183A | *-* | | sugar epimerase/dehydratase homolog | |
| HQ1224A | *-* | | probable protease | |
| HQ1229A | *-* | | conserved rnhA operon protein | |
| HQ1233A | *-* | | rhomboid family protein/GlpG-like protein | |
| HQ1235A | *-* | | probable short chain dehydrogenase/ reductase (EC 1.1.1.-) | |
| HQ1238A | *cinA* | | CinA-like domain protein | |
| HQ1241A | *nusG* | | transcription antitermination protein homolog | |
| HQ1248A | *-* | | homolog to Rieske iron sulfur protein | |
| HQ1256A | *-* | | ribosome biogenesis protein Nop10 | |
| HQ1259A | *phfZ* | | probable phenazine biosynthesis protein | |
| HQ1260A | *prt* | | predicted phosphoribosyltransferase (EC 2.4.2.-) | |
| HQ1263A | *-* | | conserved smc operon protein | |
| HQ1265A | *-* | | conserved smc operon protein | |
| HQ1271A | *kin* | | predicted archaeal sugar kinase | |
| HQ1275A | *-* | | glutathione S-transferase | |
| HQ1285A | *-* | | conserved rpo operon protein | |
| HQ1336A | *-* | | conserved rpo operon protein | |
| HQ1339A | *-* | | conserved rpo operon protein | |
| HQ1369A | *-* | | predicted ATPase | |
| HQ1377A | *-* | | hydrolase (probable hydroxyacylglutathione hydrolase) (EC 3.1.2.6) | |
| HQ1392A | *-* | | homolog of lysine biosynthesis regulator/ribosomal protein S6 modification enzyme (glutaminyl transferase) | |
| HQ1403A | *minD* | | cell division inhibitor MinD | |
| HQ1412A | *-* | | conserved cobalamine operon protein; predicted hydrolase | |
| HQ1416A | *-* | | S-adenosylmethionine-dependent methyltransferase (EC 2.1.1.-) | |
| HQ1430A | *-* | | CBS domain protein | |
| HQ1457A | *mutT* | | mut/nudix family protein | |
| HQ1461A | *-* | | probable short chain dehydrogenase/ reductase (EC 1.1.1.-) | |
| HQ1473A | *prt* | | phosphoribosyltransferase (EC 2.4.2.-) | |
| HQ1492A | *-* | | stomatin homolog | |
| HQ1514A | *-* | | S-adenosylmethionine-dependent methyltransferase homolog (EC 2.1.1.-) | |
| HQ1533A | *oxr* | | oxidoreductase aldo/keto reductase family (EC 1.1.1.-) | |
| HQ1537A | *-* | | electron transfer protein homolog | |
| HQ1555A | *-* | | probable hydrolase | |
| HQ1566A | *-* | | probable short chain dehydrogenase/ reductase (EC 1.1.1.-) | |
| HQ1567A | *-* | | metallophosphoesterase | |
| HQ1579A | *taqD* | | glycerol-3-phosphate cytidyltransferase homolog | |
| HQ1584A | *-* | | homolog to phycocyanobilin lyase subunit (EC 4.-.-.-) | |
| HQ1607A | *drg* | | GTP-binding protein | |
| HQ1609A | *-* | | conserved cox cluster protein | |
| HQ1610A | *-* | | conserved cox cluster protein | |
| HQ1612A | *-* | | conserved cox cluster protein | |
| HQ1620A | *-* | | protein N-acetyltransferase homolog | |
| HQ1634A | *-* | | conserved purK operon protein / membrane-bound mannosyltransferase | |
| HQ1656A | *cbiX* | | predicted sirohydrochlorin cobaltochelatase (EC 4.99.1.3) | |
| HQ1665A | *-* | | glutaredoxin homolog | |
| HQ1690A | *-* | | mut/nudix family protein | |
| HQ1697A | *-* | | metallo-beta-lactamase family protein / hydrolase | |
| HQ1721A | *glo* | | lactoylglutathione lyase (EC 4.4.1.5) | |
| HQ1728A | *-* | | probable glycosyl transferase | |
| HQ1741A | *-* | | probable FAD-dependent monooxygenase | |
| HQ1755A | *hadL* | | 2-haloalkanoic acid dehalogenase (EC 3.8.1.2) | |
| HQ1758A | *-* | | Predicted deacylase | |
| HQ1778A | *parA* | | parA domain protein (chromosome partitioning protein) (ATPase) | |
| HQ1799A | *-* | | Acetyltransferase, GNAT family | |
| HQ1820A | *-* | | probable short chain dehydrogenase/ reductase (EC 1.1.1.-) | |
| HQ1822A | *-* | | probable alkaline phosphatase; probable cell surface glycoprotein | |
| HQ1828A | *cbs* | | CBS domain protein | |
| HQ1830A | *-* | | conserved cobalamin operon protein | |
| HQ1840A | *-* | | probable short chain dehydrogenase/ reductase (EC 1.1.1.-) | |
| HQ1847A | *-* | | Putative acetamidase/formamidase (EC 3.5.1.-) | |
| HQ1851A | *-* | | predicted helicase (EC 3.6.1.-) | |
| HQ1869A | *-* | | rhomboid family protein/intramembrane serine protease (EC 3.4.21.-) | |
| HQ1905A | *-* | | S-adenosylmethionine-dependent methyltransferase homolog (EC 2.1.1.-) | |
| HQ1916A | *uspA* | | universal stress protein | |
| HQ1919A | *speE* | | spermidine synthase (EC 2.5.1.16) | |
| HQ1933A | *-* | | probable antibiotic hydrolase; X-Pro dipeptidyl-peptidase (S15 family); probable cocaine esterase | |
| HQ1938A | *-* | | probable cyclase | |
| HQ1941A | *xdhC* | | xanthine and CO dehydrogenases maturation factor, XdhC/CoxF family | |
| HQ1961A | *-* | | CBS-domain protein | |
| HQ1964A | *bcp* | | peroxiredoxin homolog | |
| HQ1965A | *rtcB* | | RtcB-like protein | |
| HQ1968A | *-* | | predicted dehydrogenase (EC 1.-.-.-) | |
| HQ1978A | *-* | | NCAIR mutase (PurE)-related protein | |
| HQ1999A | *-* | | predicted dehydrogenase (EC 1.-.-.-) | |
| HQ2004A | *nolA* | | NADH dehydrogenase 32K chain homolog | |
| HQ2009A | *bcp* | | thioredoxin-dependent hydroperoxide peroxidase (EC 1.11.1.-) | |
| HQ2027A | *-* | | probable ATP/GTP-binding protein | |
| HQ2028A | *nudF* | | ADP-ribose pyrophosphatase (EC 3.6.1.13) | |
| HQ2029A | *cad* | | pterin-4a-carbinolamine dehydratase (EC 4.2.1.96) | |
| HQ2035A | *-* | | mandelate racemase/muconate lactonizing enzyme family protein | |
| HQ2043A | *cinA* | | CinA-like domain protein | |
| HQ2062A | *ldcA* | | LD-carboxypeptidase | |
| HQ2150A | *-* | | putative protein-tyrosine-phosphatase | |
| HQ2153A | *-* | | predicted ATP-dependent Lon-type protease | |
| HQ2178A | *rad25* | | DNA repair helicase RAD25 | |
| HQ2202A | *-* | | PemK-like growth inhibitor protein (MazF protein) | |
| HQ2207A | *sqlE* | | probable oxidoreductase | |
| HQ2226A | *pncA* | | isochorismatase | |
| HQ2232A | *-* | | restriction system mrr homolog | |
| HQ2269A | *-* | | CBS domain membrane associated protein | |
| HQ2288A | *-* | | thiosulfate sulfurtransferase homolog (EC 2.8.1.1)/ hydrolase (probable hydroxyacylglutathione hydrolase (EC 3.1.2.6)) | |
| HQ2309A | *phaB* | | 3-oxoacyl-[acyl-carrier-protein] reductase (3-ketoacyl-acyl carrier protein reductase) (EC 1.1.1.100) PhaB | |
| HQ2310A | *phaC* | | Poly-beta-hydroxybutyrate polymerase (EC 2.3.1.-) Polyhydroxyalkanoic acid synthase PhaC | |
| HQ2325A | *-* | | probable oxidoreductase, oxygen dependent, FAD-dependent protein | |
| HQ2335A | *-* | | Nucleoside diphosphate sugar epimerase | |
| HQ2339A | *hflX* | | GTP-binding protein | |
| HQ2359A | *-* | | conserved rps operon protein | |
| HQ2360A | *-* | | conserved rps operon protein | |
| HQ2380A | *-* | | probable short chanin dehydrogenase/ reductase (EC 1.1.1.-) | |
| HQ2391A | *apa* | | probable bis(5'-nucleosyl)-tetraphosphatase (asymmetrical) (Diadenosine tetraphosphatase) | |
| HQ2398A | *-* | | probable methyltransferase | |
| HQ2401A | *glo* | | lyase / dioxygenase (probable lactoylglutathione lyase (EC 4.4.1.5), aromatic compounds dioxygenase (EC 1.13.11.-)) | |
| HQ2403A | *-* | | probable phosphoesterase | |
| HQ2406A | *-* | | predicted transporter (predicted permease) | |
| HQ2425A | *tdh* | | oxidoreductase (probable Zn-dependent dehydrogenases (EC 1.1.1.-), threonine 3-dehydrogenase (EC 1.1.1.103)) | |
| HQ2431A | *-* | | deoxyribodipyrimidine photolyase-related protein | |
| HQ2445A | *trxB* | | thioredoxin reductase homolog | |
| HQ2457A | *-* | | probable hydrolase | |
| HQ2488A | *cinA* | | CinA-like domain protein | |
| HQ2522A | *-* | | protein N-acetyltransferase homolog | |
| HQ2528A | *rhl* | | DNA repair helicase | |
| HQ2566A | *gcs2* | | carboxylate-amine ligase (EC 6.3.-.-) | |
| HQ2587A | *minD* | | cell division inhibitor MinD-like (ATPase involved in chromosome partitioning) | |
| HQ2595A | *pat* | | acetyltransferase protein (conserved in phopsphonate uptake operon) (EC 2.3.1.-) | |
| HQ2628A | *gbp* | | probable GTP-binding protein | |
| HQ2634A | *rad25* | | DNA repair protein Rad25 | |
| HQ2664A | *-* | | CBS domain protein, probable transcriptional regulator | |
| HQ2678A | *-* | | S-adenosylmethionine-dependent methyltransferase homolog (EC 2.1.1.-) | |
| HQ2686A | *-* | | probable hexosyltransferase; glycosyltransferase | |
| HQ2711A | *-* | | probable acetyltransferase | |
| HQ2716A | *-* | | AAA-type ATPase (transitional ATPase homolog) | |
| HQ2717A | *bcp* | | peroxiredoxin (thioredoxin-dependent hydroperoxide peroxidase) (EC 1.11.1.-) | |
| HQ2718A | *-* | | conserved primase operon protein | |
| HQ2721A | *-* | | hesB/IscA family protein | |
| HQ2733A | *ctaA* | | cytochrome-c-aa3 oxidase assembly factor CtaA | |
| HQ2743A | *-* | | ribosomal protein S10 homolog | |
| HQ2744A | *-* | | indole-3-acetyl-L-aspartic acid hydrolase (EC 3.5.1.-) | |
| HQ2753A | *cdc48* | | AAA-type ATPase (transitional ATPase homolog) | |
| HQ2769A | *ephA* | | epoxide hydrolase-related protein | |
| HQ2771A | *-* | | 3-oxoacyl-[acyl-carrier-protein] reductase (3-ketoacyl-acyl carrier protein reductase) (EC 1.1.1.100) | |
| HQ2775A | *-* | | protein N-acetyltransferase | |
| HQ2799A | *caaX* | | CAAX prenyl proteinase / zinc metalloproteinase | |
| HQ2843A | *-* | | conserved rpl operon protein | |
| HQ2861A | *-* | | probable carotenoid biosynthesis protein | |
| HQ2866A | *-* | | metalloenzyme, probable TatD related DNAse | |
| HQ2871A | *-* | | acetyltransferase | |
| HQ2890A | *-* | | predicted hydrolase | |
| HQ2891A | *-* | | small nuclear ribonucleoprotein (snRNP) homolog | |
| HQ2899A | *-* | | Predicted Zn-ribbon RNA-binding protein | |
| HQ2927A | *elaC* | | Ribonuclease Z (RNase Z) (tRNA 3 endonuclease) (EC 3.1.26.11) | |
| HQ2945A | *stt3* | | transmembrane oligosaccharyl transferase | |
| HQ2947A | *guaA* | | guanosine monophosphate synthetase (glutamine-hydrolyzing) (GMP synthase) | |
| HQ2949A | *mrp* | | ATP-binding protein Mrp | |
| HQ2961A | *-* | | protein N-acetyltransferase homolog | |
| HQ2974A | *-* | | predicted metal-dependent hydrolase of the TIM-barrel fold | |
| HQ3000A | *spoVR* | | spore cortext formation protein homolog | |
| HQ3001A | *-* | | conserved prkA cluster protein | |
| HQ3017A | *-* | | conserved acc operon protein | |
| HQ3020A | *bcp* | | peroxiredoxin homolog, bacterioferritin comigrating protein | |
| HQ3031A | *-* | | conserved cobalamin operon protein | |
| HQ3046A | *-* | | conserved coaE operon protein | |
| HQ3060A | *-* | | serine/threonine protein kinase related protein | |
| HQ3065A | *-* | | superfamily II helicase | |
| HQ3070A | *-* | | predicted ATPase | |
| HQ3084A | *nudF* | | ADP-ribose pyrophosphatase (EC 3.6.1.13); Mut/nudix family protein | |
| HQ3104A | *-* | | AAA-type ATPase | |
| HQ3105A | *phnP* | | PhnP-like protein | |
| HQ3107A | *nifUx* | | nifU protein C-terminal domain homolog | |
| HQ3118A | *rimI* | | protein N-acetyltransferase | |
| HQ3119A | *-* | | predicted NAD/FAD-dependent oxidoreductase | |
| HQ3122A | *trpD* | | anthranilate phosphoribosyltransferase (EC 2.4.2.18) | |
| HQ3137A | *-* | | hydrolase (probable hydroxyacylglutathione hydrolase (EC 3.1.2.6)) | |
| HQ3140A | *-* | | GTP-binding protein | |
| HQ3146A | *nolA* | | NADH dehydrogenase 32K chain | |
| HQ3150A | *engB* | | probable GTP-binding protein | |
| HQ3152A | *-* | | homolog to thiosulfate sulfurtransferase (EC 2.8.1.1) | |
| HQ3172A | *cbs* | | CBS domain protein | |
| HQ3177A | *aaa* | | AAA-type ATPase (transitional ATPase homolog) | |
| HQ3180A | *vacB* | | ribonuclease R homolog | |
| HQ3191A | *ghmP* | | GHMP family kinase | |
| HQ3209A | *pphA* | | serine/threonine protein phosphatase | |
| HQ3263A | *-* | | replication factor A homolog | |
| HQ3266A | *-* | | homolog to predicted helicase (EC 3.6.1.-) | |
| HQ3270A | *-* | | hypothetical protein; probable calcium binding | |
| HQ3271A | *int* | | phage integrase | |
| HQ3274A | *-* | | predicted helicase (EC 3.6.1.-) | |
| HQ3284A | *htpX* | | protease htpX protein homolog | |
| HQ3297A | *-* | | AAA-type ATPase (cell division control protein homolog) | |
| HQ3351A | *-* | | conserved tyrA operon protein | |
| HQ3365A | *-* | | oxidoreductase homolog | |
| HQ3387A | *-* | | predicted allosteric regulator of homoserine dehydrogenase | |
| HQ3403A | *elaC* | | ribonuclease Z (RNase Z) (tRNA 3 endonuclease) (EC 3.1.26.11) | |
| HQ3425A | *-* | | tetratricopeptide repeat protein | |
| HQ3428A | *nifU* | | homolog to nitrogen fixation protein NifU | |
| HQ3431A | *htpX* | | htpX protein homolog | |
| HQ3436A | *dinG* | | Rad3-related DNA helicases DinG | |
| HQ3460A | *-* | | conserved polA operon protein (predicted DNA-binding protein) | |
| HQ3465A | *-* | | nifU protein C-terminal domain homolog | |
| HQ3483A | *mutT* | | Mut/nudix family protein | |
| HQ3512A | *-* | | probable SAM-dependent methyltransferase | |
| HQ3602A | *bcp* | | peroxiredoxin | |
| HQ3640A | *-* | | phosphoesterase, metallo-phosphoesterase-calcineurin-like | |
| HQ3656A | *minD* | | cell division inhibitor MinD-like (ATPase involved in chromosome partitioning) | |
| HQ3668A | *guaA* | | guanosine monophosphate synthetase (glutamine-hydrolyzing) (GMP synthase) | |
| HQ3672A | *-* | | probable acetyltransferase (GNAT) family | |
| HQ3707A | *-* | | peroxiredoxin homolog | |
| HQ3710A | *-* | | homolog to dolichol kinase | |
| HQ3713A | *-* | | arg cluster protein | |
| HQ3733A | *-* | | probable S-adenosylmethionine-dependent methyltransferase (24-sterol C-methyltransferase homolog) (EC 2.1.1.-) | |
| HQ3738A | *-* | | probable GTP-binding protein | |
| HQ4026A | *parA* | | parA domain protein (chromosome partitioning protein) (ATPase) | |
| HQ4032A | *mrr* | | restriction system mrr homolog | |
| UNASS |  | | unassigned | |
| **CHY** |  | | **conserved hypothetical protein** | |
| ***code*** | ***gene*** | | ***protein name*** | |
| HQ1004A | *-* | | conserved hypothetical protein | |
| HQ1007A | *-* | | conserved hypothetical protein | |
| HQ1009A | *-* | | conserved hypothetical protein | |
| HQ1010A | *-* | | conserved hypothetical protein | |
| HQ1013A | *-* | | conserved hypothetical protein | |
| HQ1015A | *-* | | conserved hypothetical protein | |
| HQ1023A | *-* | | conserved hypothetical protein | |
| HQ1027A | *-* | | conserved hypothetical protein | |
| HQ1028A | *-* | | conserved hypothetical protein | |
| HQ1030A | *-* | | conserved hypothetical protein | |
| HQ1031A | *-* | | conserved hypothetical protein | |
| HQ1034A | *-* | | conserved hypothetical protein | |
| HQ1037A | *-* | | conserved hypothetical protein | |
| HQ1038A | *-* | | conserved hypothetical protein | |
| HQ1039A | *-* | | conserved hypothetical protein | |
| HQ1040A | *-* | | conserved hypothetical protein | |
| HQ1053A | *-* | | conserved hypothetical protein | |
| HQ1055A | *-* | | conserved hypothetical protein | |
| HQ1057A | *-* | | conserved hypothetical protein | |
| HQ1059A | *-* | | conserved hypothetical protein | |
| HQ1062A | *-* | | conserved hypothetical protein | |
| HQ1067A | *-* | | conserved hypothetical protein | |
| HQ1068A | *-* | | conserved hypothetical protein | |
| HQ1071A | *-* | | conserved hypothetical protein | |
| HQ1077A | *-* | | conserved hypothetical protein | |
| HQ1078A | *-* | | conserved hypothetical protein | |
| HQ1079A | *-* | | prophage maintenance system killer protein homolog | |
| HQ1086A | *-* | | conserved hypothetical protein | |
| HQ1087A | *-* | | conserved hypothetical protein | |
| HQ1088A | *-* | | conserved hypothetical protein | |
| HQ1089A | *-* | | conserved hypothetical protein | |
| HQ1090A | *-* | | conserved hypothetical protein | |
| HQ1094A | *-* | | conserved hypothetical protein | |
| HQ1100A | *-* | | conserved hypothetical protein | |
| HQ1103A | *-* | | conserved hypothetical protein | |
| HQ1106A | *-* | | conserved hypothetical protein | |
| HQ1109A | *-* | | conserved hypothetical protein | |
| HQ1112A | *-* | | conserved hypothetical protein | |
| HQ1113A | *-* | | conserved hypothetical protein | |
| HQ1115A | *-* | | conserved hypothetical protein | |
| HQ1117A | *-* | | conserved hypothetical protein | |
| HQ1134A | *-* | | conserved hypothetical protein | |
| HQ1136A | *-* | | conserved hypothetical protein | |
| HQ1137A | *-* | | conserved hypothetical protein | |
| HQ1140A | *-* | | conserved hypothetical protein | |
| HQ1141A | *-* | | conserved hypothetical protein | |
| HQ1149A | *-* | | conserved hypothetical protein | |
| HQ1162A | *-* | | conserved hypothetical protein | |
| HQ1171A | *-* | | conserved hypothetical protein | |
| HQ1172A | *-* | | conserved hypothetical protein | |
| HQ1174A | *-* | | conserved hypothetical protein | |
| HQ1176A | *-* | | conserved hypothetical protein | |
| HQ1178A | *-* | | nudix family protein | |
| HQ1179A | *-* | | conserved hypothetical protein | |
| HQ1184A | *-* | | conserved hypothetical protein | |
| HQ1185A | *-* | | conserved hypothetical protein | |
| HQ1186A | *-* | | conserved hypothetical protein | |
| HQ1187A | *-* | | conserved hypothetical protein | |
| HQ1188A | *-* | | conserved hypothetical protein | |
| HQ1189A | *-* | | conserved hypothetical protein | |
| HQ1192A | *-* | | probable dentin sialophosphoprotein precursor | |
| HQ1194A | *-* | | conserved hypothetical protein | |
| HQ1201A | *-* | | conserved hypothetical protein | |
| HQ1203A | *-* | | conserved hypothetical protein | |
| HQ1212A | *-* | | conserved hypothetical protein | |
| HQ1214A | *-* | | conserved hypothetical protein (probable surface protein) | |
| HQ1217A | *-* | | conserved hypothetical protein | |
| HQ1218A | *-* | | conserved hypothetical protein | |
| HQ1219A | *-* | | conserved hypothetical protein | |
| HQ1221A | *-* | | conserved hypothetical protein | |
| HQ1222A | *-* | | conserved hypothetical protein | |
| HQ1223A | *-* | | conserved hypothetical protein | |
| HQ1226A | *-* | | conserved hypothetical protein | |
| HQ1232A | *-* | | conserved hypothetical protein | |
| HQ1239A | *-* | | conserved hypothetical protein | |
| HQ1240A | *-* | | conserved hypothetical protein | |
| HQ1244A | *-* | | conserved hypothetical protein | |
| HQ1246A | *-* | | conserved hypothetical protein | |
| HQ1251A | *-* | | conserved hypothetical protein | |
| HQ1252A | *-* | | conserved hypothetical protein | |
| HQ1257A | *-* | | conserved hypothetical protein | |
| HQ1272A | *-* | | conserved hypothetical protein | |
| HQ1277A | *-* | | conserved hypothetical protein | |
| HQ1279A | *-* | | conserved hypothetical protein | |
| HQ1282A | *-* | | conserved hypothetical protein | |
| HQ1287A | *-* | | conserved hypothetical protein | |
| HQ1288A | *-* | | conserved hypothetical protein | |
| HQ1290A | *-* | | conserved hypothetical protein | |
| HQ1291A | *-* | | conserved hypothetical protein | |
| HQ1294A | *-* | | conserved hypothetical protein | |
| HQ1303A | *-* | | conserved hypothetical protein | |
| HQ1304A | *-* | | conserved hypothetical protein | |
| HQ1305A | *-* | | conserved hypothetical protein | |
| HQ1312A | *-* | | conserved hypothetical protein | |
| HQ1318A | *-* | | conserved hypothetical protein | |
| HQ1321A | *-* | | conserved hypothetical protein | |
| HQ1322A | *-* | | conserved hypothetical protein | |
| HQ1330A | *-* | | conserved hypothetical protein | |
| HQ1331A | *-* | | conserved hypothetical protein | |
| HQ1332A | *-* | | conserved hypothetical protein | |
| HQ1333A | *-* | | conserved hypothetical protein | |
| HQ1334A | *-* | | conserved hypothetical protein | |
| HQ1342A | *-* | | conserved hypothetical protein | |
| HQ1344A | *-* | | conserved hypothetical protein | |
| HQ1350A | *pchB* | | potassium channel protein homolog | |
| HQ1352A | *-* | | conserved hypothetical protein | |
| HQ1354A | *-* | | conserved hypothetical protein | |
| HQ1358A | *-* | | conserved hypothetical protein | |
| HQ1361A | *-* | | conserved hypothetical protein | |
| HQ1365A | *-* | | conserved hypothetical protein | |
| HQ1366A | *-* | | conserved hypothetical protein | |
| HQ1371A | *-* | | conserved hypothetical protein | |
| HQ1374A | *-* | | conserved hypothetical protein | |
| HQ1375A | *-* | | conserved hypothetical protein | |
| HQ1376A | *-* | | conserved hypothetical protein | |
| HQ1378A | *-* | | conserved hypothetical protein | |
| HQ1379A | *-* | | conserved hypothetical protein | |
| HQ1389A | *-* | | conserved hypothetical protein | |
| HQ1390A | *-* | | conserved hypothetical protein | |
| HQ1397A | *-* | | conserved hypothetical protein | |
| HQ1402A | *-* | | conserved hypothetical protein | |
| HQ1413A | *-* | | conserved hypothetical protein | |
| HQ1427A | *-* | | conserved hypothetical protein | |
| HQ1429A | *-* | | conserved hypothetical protein | |
| HQ1448A | *-* | | conserved hypothetical protein | |
| HQ1450A | *-* | | conserved hypothetical protein | |
| HQ1452A | *-* | | conserved hypothetical protein | |
| HQ1453A | *-* | | conserved hypothetical protein | |
| HQ1454A | *-* | | conserved hypothetical protein | |
| HQ1475A | *-* | | conserved hypothetical protein | |
| HQ1476A | *-* | | conserved hypothetical protein | |
| HQ1490A | *-* | | conserved hypothetical protein | |
| HQ1491A | *-* | | conserved hypothetical protein | |
| HQ1496A | *-* | | conserved hypothetical protein | |
| HQ1497A | *-* | | conserved hypothetical protein | |
| HQ1499A | *-* | | conserved hypothetical protein | |
| HQ1500A | *-* | | conserved hypothetical protein | |
| HQ1505A | *-* | | conserved hypothetical protein | |
| HQ1506A | *-* | | conserved hypothetical protein | |
| HQ1518A | *-* | | conserved hypothetical protein | |
| HQ1519A | *-* | | conserved hypothetical protein | |
| HQ1524A | *-* | | conserved hypothetical protein | |
| HQ1528A | *-* | | conserved hypothetical protein | |
| HQ1529A | *-* | | conserved hypothetical protein | |
| HQ1530A | *-* | | conserved hypothetical protein | |
| HQ1531A | *-* | | conserved hypothetical protein | |
| HQ1532A | *-* | | conserved hypothetical protein | |
| HQ1540A | *-* | | conserved hypothetical protein (probable surface protein) | |
| HQ1543A | *-* | | conserved hypothetical protein (probable surface protein) | |
| HQ1546A | *-* | | conserved hypothetical protein | |
| HQ1547A | *-* | | conserved hypothetical protein | |
| HQ1549A | *-* | | conserved hypothetical protein | |
| HQ1552A | *-* | | conserved hypothetical protein | |
| HQ1553A | *-* | | conserved hypothetical protein | |
| HQ1554A | *-* | | conserved hypothetical protein | |
| HQ1556A | *-* | | conserved hypothetical protein | |
| HQ1559A | *-* | | conserved hypothetical protein | |
| HQ1562A | *-* | | conserved hypothetical protein | |
| HQ1575A | *-* | | conserved hypothetical protein | |
| HQ1576A | *-* | | conserved hypothetical protein | |
| HQ1580A | *-* | | conserved hypothetical protein | |
| HQ1587A | *-* | | conserved hypothetical protein | |
| HQ1588A | *-* | | conserved hypothetical protein | |
| HQ1589A | *-* | | conserved hypothetical protein | |
| HQ1591A | *-* | | conserved hypothetical protein | |
| HQ1594A | *-* | | conserved hypothetical protein | |
| HQ1597A | *-* | | conserved hypothetical protein | |
| HQ1598A | *-* | | conserved hypothetical protein | |
| HQ1599A | *-* | | conserved hypothetical protein | |
| HQ1602A | *-* | | conserved hypothetical protein | |
| HQ1604A | *-* | | conserved hypothetical protein | |
| HQ1606A | *-* | | conserved hypothetical protein | |
| HQ1608A | *-* | | conserved hypothetical protein | |
| HQ1621A | *-* | | conserved hypothetical protein | |
| HQ1623A | *-* | | conserved hypothetical protein | |
| HQ1630A | *-* | | conserved hypothetical protein | |
| HQ1649A | *-* | | phosphoesterase homolog | |
| HQ1651A | *-* | | conserved hypothetical protein | |
| HQ1653A | *-* | | conserved hypothetical protein | |
| HQ1655A | *-* | | conserved hypothetical protein | |
| HQ1657A | *-* | | conserved hypothetical protein | |
| HQ1658A | *-* | | conserved hypothetical protein | |
| HQ1659A | *-* | | conserved hypothetical protein | |
| HQ1666A | *-* | | conserved hypothetical protein | |
| HQ1669A | *-* | | conserved hypothetical protein | |
| HQ1671A | *-* | | conserved hypothetical protein | |
| HQ1673A | *-* | | conserved hypothetical protein | |
| HQ1684A | *-* | | conserved hypothetical protein | |
| HQ1698A | *-* | | conserved hypothetical protein | |
| HQ1702A | *-* | | conserved hypothetical protein | |
| HQ1703A | *-* | | conserved hypothetical protein | |
| HQ1704A | *-* | | conserved hypothetical protein | |
| HQ1709A | *-* | | conserved hypothetical protein | |
| HQ1711A | *-* | | conserved hypothetical protein | |
| HQ1712A | *-* | | conserved hypothetical protein | |
| HQ1718A | *-* | | conserved hypothetical protein | |
| HQ1719A | *-* | | conserved hypothetical protein | |
| HQ1723A | *-* | | conserved hypothetical protein | |
| HQ1732A | *-* | | conserved hypothetical protein | |
| HQ1738A | *-* | | conserved hypothetical protein | |
| HQ1746A | *-* | | conserved hypothetical protein | |
| HQ1747A | *-* | | conserved hypothetical protein | |
| HQ1748A | *-* | | conserved hypothetical protein | |
| HQ1749A | *-* | | conserved hypothetical protein | |
| HQ1751A | *-* | | conserved hypothetical protein | |
| HQ1753A | *-* | | conserved hypothetical protein | |
| HQ1754A | *-* | | conserved hypothetical protein | |
| HQ1759A | *-* | | conserved hypothetical protein | |
| HQ1764A | *-* | | conserved hypothetical protein | |
| HQ1788A | *-* | | conserved hypothetical protein | |
| HQ1789A | *-* | | conserved hypothetical protein | |
| HQ1790A | *-* | | conserved hypothetical protein | |
| HQ1791A | *-* | | conserved hypothetical protein | |
| HQ1796A | *-* | | conserved hypothetical protein | |
| HQ1800A | *-* | | conserved hypothetical protein | |
| HQ1801A | *-* | | conserved hypothetical protein | |
| HQ1804A | *-* | | conserved hypothetical protein | |
| HQ1807A | *-* | | conserved hypothetical protein | |
| HQ1808A | *-* | | conserved hypothetical protein | |
| HQ1809A | *-* | | conserved hypothetical protein | |
| HQ1813A | *-* | | conserved hypothetical protein (probable surface protein) | |
| HQ1818A | *-* | | conserved hypothetical protein | |
| HQ1819A | *-* | | conserved hypothetical protein | |
| HQ1823A | *-* | | conserved hypothetical protein | |
| HQ1825A | *-* | | conserved hypothetical protein | |
| HQ1839A | *-* | | conserved hypothetical protein | |
| HQ1853A | *-* | | conserved hypothetical protein | |
| HQ1854A | *-* | | conserved hypothetical protein | |
| HQ1860A | *-* | | conserved hypothetical protein | |
| HQ1870A | *-* | | conserved hypothetical protein | |
| HQ1871A | *-* | | conserved hypothetical protein | |
| HQ1877A | *-* | | conserved hypothetical protein | |
| HQ1882A | *-* | | conserved hypothetical protein | |
| HQ1883A | *-* | | conserved hypothetical protein | |
| HQ1885A | *-* | | conserved hypothetical protein | |
| HQ1887A | *-* | | conserved hypothetical protein | |
| HQ1889A | *-* | | conserved hypothetical protein | |
| HQ1890A | *-* | | conserved hypothetical protein | |
| HQ1891A | *-* | | conserved hypothetical protein | |
| HQ1894A | *-* | | conserved hypothetical protein | |
| HQ1896A | *-* | | conserved hypothetical protein | |
| HQ1897A | *-* | | conserved hypothetical protein | |
| HQ1898A | *-* | | conserved hypothetical protein | |
| HQ1899A | *-* | | conserved hypothetical protein | |
| HQ1900A | *-* | | conserved hypothetical protein | |
| HQ1901A | *-* | | conserved hypothetical protein | |
| HQ1902A | *-* | | conserved hypothetical protein | |
| HQ1903A | *-* | | conserved hypothetical protein | |
| HQ1904A | *-* | | conserved hypothetical protein | |
| HQ1907A | *-* | | conserved hypothetical protein | |
| HQ1908A | *-* | | conserved hypothetical protein | |
| HQ1912A | *-* | | conserved hypothetical protein | |
| HQ1920A | *-* | | conserved hypothetical protein | |
| HQ1921A | *-* | | conserved hypothetical protein | |
| HQ1922A | *-* | | conserved hypothetical protein | |
| HQ1926A | *-* | | conserved hypothetical protein | |
| HQ1947A | *-* | | conserved hypothetical protein | |
| HQ1948A | *-* | | conserved hypothetical protein | |
| HQ1953A | *-* | | conserved hypothetical protein | |
| HQ1958A | *-* | | conserved hypothetical protein | |
| HQ1959A | *-* | | conserved hypothetical protein | |
| HQ1980A | *-* | | conserved hypothetical protein | |
| HQ1984A | *-* | | conserved hypothetical protein | |
| HQ1994A | *-* | | conserved hypothetical protein | |
| HQ2000A | *-* | | conserved hypothetical protein | |
| HQ2002A | *-* | | conserved hypothetical protein | |
| HQ2005A | *-* | | conserved hypothetical protein | |
| HQ2017A | *-* | | conserved hypothetical protein (probable surface protein) | |
| HQ2026A | *-* | | PHP domain protein | |
| HQ2034A | *-* | | conserved hypothetical protein | |
| HQ2039A | *-* | | conserved hypothetical protein | |
| HQ2041A | *-* | | conserved hypothetical protein | |
| HQ2055A | *-* | | conserved hypothetical protein | |
| HQ2056A | *-* | | conserved hypothetical protein | |
| HQ2057A | *-* | | conserved hypothetical protein | |
| HQ2059A | *-* | | conserved hypothetical protein | |
| HQ2061A | *-* | | conserved hypothetical protein | |
| HQ2068A | *-* | | conserved hypothetical protein | |
| HQ2083A | *-* | | probable haloviral protein | |
| HQ2084A | *-* | | conserved hypothetical protein | |
| HQ2085A | *-* | | conserved hypothetical protein | |
| HQ2086A | *-* | | conserved hypothetical protein | |
| HQ2090A | *-* | | conserved hypothetical protein | |
| HQ2092A | *-* | | conserved hypothetical protein | |
| HQ2093A | *-* | | conserved hypothetical protein | |
| HQ2097A | *-* | | conserved hypothetical protein | |
| HQ2100A | *-* | | conserved hypothetical protein | |
| HQ2101A | *-* | | conserved hypothetical protein | |
| HQ2105A | *-* | | conserved hypothetical protein | |
| HQ2106A | *-* | | conserved hypothetical protein | |
| HQ2109A | *-* | | conserved hypothetical protein | |
| HQ2113A | *-* | | conserved hypothetical protein | |
| HQ2114A | *-* | | pentapeptide repeats family protein | |
| HQ2115A | *-* | | conserved hypothetical protein | |
| HQ2117A | *-* | | conserved hypothetical protein | |
| HQ2121A | *-* | | conserved hypothetical protein | |
| HQ2123A | *-* | | conserved hypothetical protein | |
| HQ2124A | *-* | | conserved hypothetical protein | |
| HQ2128A | *-* | | conserved hypothetical protein | |
| HQ2129A | *-* | | conserved hypothetical protein | |
| HQ2130A | *-* | | conserved hypothetical protein | |
| HQ2131A | *-* | | conserved hypothetical protein | |
| HQ2132A | *-* | | conserved hypothetical protein | |
| HQ2133A | *-* | | conserved hypothetical protein | |
| HQ2134A | *-* | | conserved hypothetical protein | |
| HQ2135A | *-* | | conserved hypothetical protein | |
| HQ2136A | *-* | | conserved hypothetical protein | |
| HQ2137A | *-* | | conserved hypothetical protein | |
| HQ2138A | *-* | | conserved hypothetical protein | |
| HQ2139A | *-* | | conserved hypothetical protein | |
| HQ2140A | *-* | | conserved hypothetical protein | |
| HQ2145A | *-* | | conserved hypothetical protein | |
| HQ2146A | *-* | | conserved hypothetical protein | |
| HQ2148A | *-* | | conserved hypothetical protein | |
| HQ2152A | *-* | | conserved hypothetical protein | |
| HQ2157A | *-* | | conserved hypothetical protein | |
| HQ2158A | *-* | | conserved hypothetical protein | |
| HQ2159A | *-* | | conserved hypothetical protein | |
| HQ2160A | *-* | | conserved hypothetical protein | |
| HQ2161A | *-* | | conserved hypothetical protein | |
| HQ2162A | *-* | | conserved hypothetical protein | |
| HQ2163A | *-* | | conserved hypothetical protein | |
| HQ2164A | *-* | | conserved hypothetical protein | |
| HQ2165A | *-* | | conserved hypothetical protein | |
| HQ2167A | *-* | | conserved hypothetical protein | |
| HQ2168A | *-* | | conserved hypothetical protein | |
| HQ2169A | *-* | | conserved hypothetical protein | |
| HQ2170A | *-* | | conserved hypothetical protein | |
| HQ2171A | *-* | | conserved hypothetical protein | |
| HQ2172A | *-* | | conserved hypothetical protein | |
| HQ2173A | *-* | | conserved hypothetical protein | |
| HQ2174A | *-* | | conserved hypothetical protein | |
| HQ2175A | *-* | | conserved hypothetical protein | |
| HQ2176A | *-* | | conserved hypothetical protein | |
| HQ2177A | *-* | | conserved hypothetical protein | |
| HQ2180A | *-* | | conserved hypothetical protein | |
| HQ2183A | *-* | | conserved hypothetical protein | |
| HQ2198A | *-* | | homolog to transcription regulator | |
| HQ2201A | *-* | | conserved hypothetical protein | |
| HQ2206A | *-* | | conserved hypothetical protein | |
| HQ2208A | *-* | | conserved hypothetical protein | |
| HQ2209A | *-* | | conserved hypothetical protein | |
| HQ2214A | *-* | | conserved hypothetical protein | |
| HQ2215A | *-* | | conserved hypothetical protein | |
| HQ2217A | *-* | | conserved hypothetical protein | |
| HQ2222A | *-* | | conserved hypothetical protein | |
| HQ2223A | *-* | | conserved hypothetical protein | |
| HQ2224A | *-* | | conserved hypothetical protein | |
| HQ2228A | *-* | | conserved hypothetical protein | |
| HQ2234A | *-* | | conserved hypothetical protein | |
| HQ2235A | *-* | | conserved hypothetical protein | |
| HQ2243A | *-* | | conserved hypothetical protein | |
| HQ2249A | *-* | | conserved hypothetical protein | |
| HQ2253A | *-* | | conserved hypothetical protein | |
| HQ2254A | *-* | | conserved hypothetical protein | |
| HQ2255A | *-* | | conserved hypothetical protein | |
| HQ2256A | *-* | | conserved hypothetical protein | |
| HQ2257A | *-* | | conserved hypothetical protein | |
| HQ2258A | *-* | | conserved hypothetical protein | |
| HQ2260A | *-* | | conserved hypothetical protein | |
| HQ2262A | *-* | | conserved hypothetical protein | |
| HQ2263A | *-* | | conserved hypothetical protein | |
| HQ2271A | *-* | | ferredoxin domain protein | |
| HQ2273A | *-* | | conserved hypothetical protein | |
| HQ2279A | *-* | | conserved hypothetical protein | |
| HQ2281A | *-* | | conserved hypothetical protein | |
| HQ2282A | *-* | | conserved hypothetical protein | |
| HQ2283A | *-* | | conserved hypothetical protein | |
| HQ2287A | *-* | | conserved hypothetical protein | |
| HQ2289A | *-* | | conserved hypothetical protein | |
| HQ2294A | *-* | | conserved hypothetical protein | |
| HQ2308A | *-* | | conserved hypothetical protein | |
| HQ2311A | *-* | | phbC-phbA intergenic region protein | |
| HQ2312A | *-* | | conserved hypothetical protein | |
| HQ2313A | *-* | | conserved hypothetical protein | |
| HQ2316A | *-* | | conserved hypothetical protein | |
| HQ2320A | *-* | | conserved hypothetical protein | |
| HQ2323A | *-* | | conserved hypothetical protein | |
| HQ2328A | *-* | | conserved hypothetical protein | |
| HQ2329A | *glpG* | | rhomboid family protein | |
| HQ2330A | *-* | | conserved hypothetical protein | |
| HQ2331A | *-* | | conserved hypothetical protein | |
| HQ2338A | *-* | | conserved hypothetical protein | |
| HQ2343A | *-* | | conserved hypothetical protein | |
| HQ2345A | *-* | | conserved hypothetical protein | |
| HQ2347A | *-* | | conserved hypothetical protein | |
| HQ2349A | *-* | | conserved hypothetical protein | |
| HQ2350A | *-* | | conserved hypothetical protein | |
| HQ2355A | *-* | | conserved hypothetical protein | |
| HQ2362A | *-* | | conserved hypothetical protein | |
| HQ2364A | *-* | | conserved hypothetical protein | |
| HQ2368A | *-* | | homolog to lyase/ dioxygenase | |
| HQ2371A | *-* | | conserved hypothetical protein | |
| HQ2378A | *-* | | conserved hypothetical protein | |
| HQ2384A | *-* | | conserved hypothetical protein | |
| HQ2385A | *-* | | conserved hypothetical protein | |
| HQ2388A | *-* | | conserved hypothetical protein | |
| HQ2392A | *-* | | conserved hypothetical protein | |
| HQ2393A | *-* | | conserved hypothetical protein | |
| HQ2395A | *-* | | conserved hypothetical protein | |
| HQ2397A | *-* | | conserved hypothetical protein | |
| HQ2399A | *-* | | conserved hypothetical protein | |
| HQ2404A | *-* | | conserved hypothetical protein | |
| HQ2408A | *-* | | conserved hypothetical protein | |
| HQ2409A | *-* | | conserved hypothetical protein | |
| HQ2410A | *-* | | conserved hypothetical protein | |
| HQ2411A | *-* | | conserved hypothetical protein | |
| HQ2413A | *-* | | conserved hypothetical protein | |
| HQ2415A | *-* | | conserved hypothetical protein | |
| HQ2417A | *-* | | conserved hypothetical protein | |
| HQ2421A | *-* | | conserved hypothetical protein | |
| HQ2422A | *-* | | conserved hypothetical protein | |
| HQ2429A | *-* | | conserved hypothetical protein | |
| HQ2435A | *-* | | conserved hypothetical protein | |
| HQ2437A | *-* | | conserved hypothetical protein | |
| HQ2442A | *-* | | conserved hypothetical protein | |
| HQ2447A | *-* | | conserved hypothetical protein | |
| HQ2458A | *-* | | conserved hypothetical protein | |
| HQ2462A | *-* | | conserved hypothetical protein | |
| HQ2464A | *-* | | conserved hypothetical protein | |
| HQ2466A | *-* | | conserved hypothetical protein | |
| HQ2469A | *-* | | conserved hypothetical protein | |
| HQ2470A | *-* | | conserved hypothetical protein | |
| HQ2474A | *-* | | conserved hypothetical protein | |
| HQ2479A | *-* | | conserved hypothetical protein | |
| HQ2489A | *-* | | conserved hypothetical protein | |
| HQ2490A | *-* | | conserved hypothetical protein | |
| HQ2492A | *-* | | conserved hypothetical protein | |
| HQ2496A | *-* | | conserved hypothetical protein | |
| HQ2502A | *-* | | conserved hypothetical protein | |
| HQ2504A | *-* | | conserved hypothetical protein | |
| HQ2507A | *-* | | conserved hypothetical protein | |
| HQ2512A | *-* | | conserved hypothetical protein | |
| HQ2517A | *-* | | conserved hypothetical protein | |
| HQ2520A | *-* | | conserved hypothetical protein | |
| HQ2524A | *-* | | conserved hypothetical protein | |
| HQ2529A | *-* | | conserved hypothetical protein | |
| HQ2530A | *-* | | conserved hypothetical protein | |
| HQ2531A | *btpA* | | photosystem I biogenesis protein homolog | |
| HQ2535A | *-* | | conserved hypothetical protein | |
| HQ2538A | *-* | | conserved hypothetical protein | |
| HQ2546A | *-* | | conserved hypothetical protein | |
| HQ2547A | *-* | | conserved hypothetical protein | |
| HQ2552A | *-* | | conserved hypothetical protein | |
| HQ2555A | *-* | | conserved hypothetical protein | |
| HQ2559A | *-* | | thioredoxin domain containing protein | |
| HQ2561A | *-* | | acetyltransferase homolog | |
| HQ2563A | *-* | | conserved hypothetical protein | |
| HQ2569A | *-* | | conserved hypothetical protein | |
| HQ2570A | *-* | | conserved hypothetical protein | |
| HQ2574A | *-* | | conserved hypothetical protein | |
| HQ2575A | *-* | | conserved hypothetical protein | |
| HQ2576A | *-* | | conserved hypothetical protein | |
| HQ2582A | *-* | | conserved hypothetical protein | |
| HQ2583A | *-* | | conserved hypothetical protein | |
| HQ2588A | *-* | | conserved hypothetical protein | |
| HQ2589A | *-* | | conserved hypothetical protein | |
| HQ2591A | *-* | | conserved hypothetical protein | |
| HQ2592A | *-* | | conserved hypothetical protein | |
| HQ2606A | *-* | | conserved hypothetical protein | |
| HQ2610A | *-* | | conserved hypothetical protein | |
| HQ2611A | *-* | | conserved hypothetical protein / permease | |
| HQ2615A | *-* | | conserved hypothetical protein | |
| HQ2617A | *-* | | conserved hypothetical protein | |
| HQ2618A | *-* | | conserved hypothetical protein | |
| HQ2622A | *-* | | conserved hypothetical protein | |
| HQ2623A | *-* | | conserved hypothetical protein | |
| HQ2624A | *-* | | conserved hypothetical protein | |
| HQ2626A | *-* | | conserved hypothetical protein | |
| HQ2627A | *-* | | conserved hypothetical protein | |
| HQ2635A | *-* | | conserved hypothetical protein | |
| HQ2636A | *-* | | conserved hypothetical protein | |
| HQ2638A | *-* | | conserved hypothetical protein | |
| HQ2642A | *-* | | conserved hypothetical protein | |
| HQ2643A | *-* | | homolog to acetyltransferases | |
| HQ2644A | *-* | | conserved hypothetical protein | |
| HQ2658A | *-* | | conserved hypothetical protein | |
| HQ2660A | *-* | | conserved hypothetical protein | |
| HQ2663A | *-* | | conserved hypothetical protein | |
| HQ2669A | *-* | | conserved hypothetical protein | |
| HQ2670A | *-* | | conserved hypothetical protein | |
| HQ2676A | *-* | | conserved hypothetical protein | |
| HQ2677A | *-* | | conserved hypothetical protein | |
| HQ2679A | *-* | | conserved hypothetical protein | |
| HQ2685A | *-* | | conserved hypothetical protein | |
| HQ2688A | *-* | | conserved hypothetical protein | |
| HQ2695A | *-* | | conserved hypothetical protein | |
| HQ2696A | *-* | | conserved hypothetical protein | |
| HQ2697A | *-* | | conserved hypothetical protein | |
| HQ2698A | *-* | | conserved hypothetical protein | |
| HQ2699A | *-* | | conserved hypothetical protein | |
| HQ2712A | *-* | | conserved hypothetical protein | |
| HQ2715A | *-* | | conserved hypothetical protein | |
| HQ2722A | *-* | | conserved hypothetical protein | |
| HQ2724A | *-* | | conserved hypothetical protein | |
| HQ2727A | *-* | | conserved hypothetical protein | |
| HQ2737A | *-* | | conserved hypothetical protein | |
| HQ2739A | *-* | | conserved hypothetical protein | |
| HQ2741A | *-* | | conserved hypothetical protein | |
| HQ2747A | *-* | | conserved hypothetical protein | |
| HQ2748A | *-* | | conserved hypothetical protein | |
| HQ2760A | *-* | | conserved hypothetical protein | |
| HQ2766A | *-* | | conserved hypothetical protein | |
| HQ2768A | *-* | | conserved hypothetical protein | |
| HQ2770A | *-* | | conserved hypothetical protein | |
| HQ2778A | *-* | | conserved hypothetical protein | |
| HQ2780A | *-* | | conserved hypothetical protein | |
| HQ2782A | *-* | | conserved hypothetical protein | |
| HQ2783A | *-* | | conserved hypothetical protein | |
| HQ2785A | *-* | | conserved hypothetical protein | |
| HQ2786A | *-* | | conserved hypothetical protein | |
| HQ2789A | *-* | | conserved hypothetical protein | |
| HQ2792A | *-* | | conserved hypothetical protein | |
| HQ2793A | *-* | | conserved hypothetical protein | |
| HQ2794A | *-* | | conserved hypothetical protein | |
| HQ2797A | *-* | | conserved hypothetical protein | |
| HQ2804A | *-* | | conserved hypothetical protein | |
| HQ2813A | *-* | | conserved hypothetical protein | |
| HQ2844A | *-* | | conserved hypothetical protein | |
| HQ2845A | *-* | | conserved hypothetical protein | |
| HQ2846A | *-* | | conserved hypothetical protein | |
| HQ2853A | *nif3* | | conserved hypothetical protein | |
| HQ2854A | *-* | | conserved hypothetical protein | |
| HQ2855A | *-* | | conserved hypothetical protein | |
| HQ2856A | *-* | | conserved hypothetical protein | |
| HQ2865A | *-* | | conserved hypothetical protein | |
| HQ2867A | *-* | | conserved hypothetical protein | |
| HQ2870A | *-* | | conserved hypothetical protein | |
| HQ2872A | *-* | | conserved hypothetical protein | |
| HQ2877A | *-* | | conserved hypothetical protein | |
| HQ2886A | *-* | | conserved hypothetical protein | |
| HQ2887A | *-* | | conserved hypothetical protein | |
| HQ2894A | *-* | | conserved hypothetical protein | |
| HQ2900A | *-* | | conserved hypothetical protein | |
| HQ2916A | *-* | | conserved hypothetical protein | |
| HQ2917A | *-* | | conserved hypothetical protein | |
| HQ2918A | *-* | | conserved hypothetical protein | |
| HQ2931A | *-* | | conserved hypothetical protein | |
| HQ2932A | *-* | | conserved hypothetical protein | |
| HQ2933A | *-* | | conserved hypothetical protein | |
| HQ2950A | *-* | | conserved hypothetical protein | |
| HQ2951A | *-* | | conserved hypothetical protein | |
| HQ2953A | *-* | | cell division control protein cdc6 homolog | |
| HQ2955A | *-* | | cell division control protein cdc6 homolog | |
| HQ2956A | *-* | | conserved hypothetical protein | |
| HQ2958A | *-* | | conserved hypothetical protein | |
| HQ2966A | *-* | | conserved hypothetical protein | |
| HQ2967A | *-* | | conserved hypothetical protein | |
| HQ2971A | *-* | | conserved hypothetical protein | |
| HQ2973A | *-* | | conserved hypothetical protein | |
| HQ2975A | *-* | | conserved hypothetical protein | |
| HQ2978A | *-* | | conserved hypothetical protein | |
| HQ2979A | *-* | | conserved hypothetical protein | |
| HQ2982A | *-* | | conserved hypothetical protein | |
| HQ2993A | *-* | | conserved hypothetical protein | |
| HQ2998A | *-* | | conserved hypothetical protein | |
| HQ2999A | *-* | | conserved hypothetical protein | |
| HQ3004A | *-* | | conserved hypothetical protein | |
| HQ3005A | *-* | | succinylglutamate desuccinylase homolog | |
| HQ3006A | *-* | | conserved hypothetical protein | |
| HQ3009A | *-* | | conserved hypothetical protein | |
| HQ3011A | *-* | | conserved hypothetical protein | |
| HQ3012A | *-* | | conserved hypothetical protein | |
| HQ3015A | *-* | | conserved hypothetical protein | |
| HQ3023A | *-* | | conserved hypothetical protein | |
| HQ3025A | *-* | | conserved hypothetical protein | |
| HQ3026A | *-* | | conserved hypothetical protein | |
| HQ3027A | *-* | | conserved hypothetical protein | |
| HQ3028A | *-* | | conserved hypothetical protein | |
| HQ3030A | *-* | | conserved hypothetical protein | |
| HQ3032B | *-* | | conserved cobalamin operon protein | |
| HQ3040A | *-* | | conserved hypothetical protein | |
| HQ3043A | *-* | | conserved hypothetical protein | |
| HQ3045A | *-* | | conserved hypothetical protein | |
| HQ3050A | *-* | | conserved hypothetical protein | |
| HQ3054A | *-* | | conserved hypothetical protein | |
| HQ3055A | *-* | | conserved hypothetical protein | |
| HQ3056A | *-* | | conserved hypothetical protein | |
| HQ3061A | *-* | | conserved hypothetical protein | |
| HQ3067A | *-* | | conserved hypothetical protein | |
| HQ3068A | *-* | | conserved hypothetical protein | |
| HQ3069A | *-* | | conserved hypothetical protein | |
| HQ3071A | *-* | | conserved hypothetical protein | |
| HQ3073A | *-* | | conserved hypothetical protein | |
| HQ3075A | *-* | | conserved hypothetical protein | |
| HQ3076A | *-* | | conserved hypothetical protein | |
| HQ3078A | *-* | | conserved hypothetical protein | |
| HQ3079A | *-* | | azlC associated membrane protein homolog | |
| HQ3083A | *-* | | conserved hypothetical protein | |
| HQ3085A | *-* | | conserved hypothetical protein | |
| HQ3087A | *-* | | conserved hypothetical protein | |
| HQ3088A | *-* | | conserved hypothetical protein | |
| HQ3089A | *-* | | conserved hypothetical protein | |
| HQ3090A | *-* | | conserved hypothetical protein | |
| HQ3095A | *-* | | conserved hypothetical protein | |
| HQ3099A | *-* | | conserved hypothetical protein | |
| HQ3100A | *-* | | conserved hypothetical protein | |
| HQ3106A | *-* | | conserved hypothetical protein | |
| HQ3109A | *-* | | conserved hypothetical protein | |
| HQ3110A | *-* | | conserved hypothetical protein | |
| HQ3111A | *-* | | conserved hypothetical protein | |
| HQ3120A | *-* | | conserved hypothetical protein | |
| HQ3124A | *-* | | conserved hypothetical protein | |
| HQ3126A | *-* | | conserved hypothetical protein | |
| HQ3132A | *-* | | conserved hypothetical protein | |
| HQ3133A | *-* | | conserved hypothetical protein | |
| HQ3134A | *-* | | conserved hypothetical protein | |
| HQ3139A | *-* | | conserved hypothetical protein | |
| HQ3141A | *-* | | conserved hypothetical protein | |
| HQ3142A | *-* | | conserved hypothetical protein | |
| HQ3144A | *-* | | conserved hypothetical protein | |
| HQ3148A | *-* | | conserved hypothetical protein | |
| HQ3149A | *-* | | conserved hypothetical protein | |
| HQ3151A | *-* | | conserved hypothetical protein | |
| HQ3160A | *-* | | conserved hypothetical protein | |
| HQ3161A | *-* | | conserved hypothetical protein | |
| HQ3164A | *-* | | conserved hypothetical protein | |
| HQ3165A | *-* | | conserved hypothetical protein | |
| HQ3174A | *-* | | conserved hypothetical protein | |
| HQ3176A | *-* | | conserved hypothetical protein | |
| HQ3179A | *-* | | conserved hypothetical protein | |
| HQ3181A | *-* | | conserved hypothetical protein | |
| HQ3188A | *-* | | conserved hypothetical protein | |
| HQ3192A | *-* | | conserved hypothetical protein | |
| HQ3193A | *-* | | conserved hypothetical protein | |
| HQ3196A | *-* | | conserved hypothetical protein | |
| HQ3200A | *-* | | conserved hypothetical protein | |
| HQ3204A | *-* | | conserved hypothetical protein | |
| HQ3213A | *-* | | conserved hypothetical protein | |
| HQ3214A | *-* | | conserved hypothetical protein | |
| HQ3220A | *-* | | conserved hypothetical protein | |
| HQ3224A | *-* | | conserved hypothetical protein | |
| HQ3226A | *-* | | conserved hypothetical protein | |
| HQ3231A | *-* | | conserved hypothetical protein | |
| HQ3232A | *-* | | conserved hypothetical protein | |
| HQ3238A | *-* | | conserved hypothetical protein | |
| HQ3252A | *-* | | conserved hypothetical protein | |
| HQ3253A | *-* | | conserved hypothetical protein | |
| HQ3254A | *-* | | conserved hypothetical protein | |
| HQ3256A | *-* | | conserved hypothetical protein | |
| HQ3257A | *-* | | conserved hypothetical protein | |
| HQ3259A | *-* | | conserved hypothetical protein | |
| HQ3262A | *-* | | conserved hypothetical protein | |
| HQ3264A | *-* | | conserved hypothetical protein | |
| HQ3265A | *-* | | conserved hypothetical protein | |
| HQ3272A | *-* | | conserved hypothetical protein | |
| HQ3283A | *-* | | conserved hypothetical protein | |
| HQ3288A | *-* | | conserved hypothetical protein | |
| HQ3291A | *-* | | bacterial conjugation protein homolog | |
| HQ3293A | *-* | | conserved hypothetical protein | |
| HQ3295A | *-* | | conserved hypothetical protein | |
| HQ3298A | *-* | | conserved hypothetical protein | |
| HQ3306A | *-* | | conserved hypothetical protein | |
| HQ3310A | *-* | | conserved hypothetical protein | |
| HQ3312A | *-* | | conserved hypothetical protein | |
| HQ3313A | *-* | | conserved hypothetical protein | |
| HQ3315A | *fsxA* | | FsxA protein (similar to protein affecting phage T7 exclusion by the F plasmid) | |
| HQ3316A | *-* | | conserved hypothetical protein | |
| HQ3317A | *-* | | conserved hypothetical protein | |
| HQ3318A | *-* | | conserved hypothetical protein | |
| HQ3320A | *-* | | conserved hypothetical protein | |
| HQ3324A | *-* | | conserved hypothetical protein | |
| HQ3325A | *-* | | conserved hypothetical protein | |
| HQ3327A | *-* | | conserved hypothetical protein | |
| HQ3330A | *-* | | conserved hypothetical protein | |
| HQ3333A | *-* | | conserved hypothetical protein | |
| HQ3339A | *-* | | conserved hypothetical protein | |
| HQ3366A | *-* | | conserved hypothetical protein | |
| HQ3368A | *-* | | conserved hypothetical protein | |
| HQ3372A | *-* | | conserved hypothetical protein | |
| HQ3373A | *-* | | conserved hypothetical protein | |
| HQ3376A | *-* | | conserved hypothetical protein | |
| HQ3389A | *-* | | conserved hypothetical protein | |
| HQ3402A | *-* | | conserved hypothetical protein | |
| HQ3404A | *-* | | conserved hypothetical protein | |
| HQ3411A | *-* | | conserved hypothetical protein | |
| HQ3426A | *-* | | conserved hypothetical protein | |
| HQ3430A | *-* | | conserved hypothetical protein | |
| HQ3437A | *-* | | conserved hypothetical protein | |
| HQ3438A | *-* | | conserved hypothetical protein | |
| HQ3439A | *-* | | conserved hypothetical protein | |
| HQ3440A | *-* | | conserved hypothetical protein | |
| HQ3441A | *-* | | conserved hypothetical protein | |
| HQ3442A | *-* | | conserved hypothetical protein | |
| HQ3458A | *-* | | conserved hypothetical protein | |
| HQ3463A | *-* | | phosphatase homolog | |
| HQ3466A | *-* | | conserved hypothetical protein | |
| HQ3471A | *-* | | conserved hypothetical protein | |
| HQ3473A | *-* | | conserved hypothetical protein | |
| HQ3475A | *-* | | conserved hypothetical protein | |
| HQ3480A | *-* | | conserved hypothetical protein | |
| HQ3482A | *-* | | conserved hypothetical protein | |
| HQ3485A | *-* | | conserved hypothetical protein | |
| HQ3486A | *-* | | conserved hypothetical protein | |
| HQ3494A | *-* | | conserved hypothetical protein | |
| HQ3497A | *-* | | conserved hypothetical protein | |
| HQ3499A | *-* | | conserved hypothetical protein | |
| HQ3500A | *-* | | conserved hypothetical protein | |
| HQ3505A | *-* | | conserved hypothetical protein | |
| HQ3506A | *-* | | conserved hypothetical protein | |
| HQ3511A | *-* | | conserved hypothetical protein | |
| HQ3513A | *-* | | conserved hypothetical protein | |
| HQ3515A | *-* | | conserved hypothetical protein | |
| HQ3522A | *-* | | conserved hypothetical protein | |
| HQ3525A | *-* | | conserved hypothetical protein | |
| HQ3530A | *-* | | conserved hypothetical protein | |
| HQ3537A | *-* | | conserved hypothetical protein | |
| HQ3538A | *-* | | conserved hypothetical protein | |
| HQ3542A | *-* | | conserved hypothetical protein | |
| HQ3548A | *-* | | conserved hypothetical protein | |
| HQ3550A | *-* | | conserved hypothetical protein | |
| HQ3561A | *-* | | conserved hypothetical protein | |
| HQ3563A | *-* | | conserved hypothetical protein | |
| HQ3564A | *-* | | conserved hypothetical protein | |
| HQ3565A | *-* | | conserved hypothetical protein | |
| HQ3567A | *-* | | conserved hypothetical protein | |
| HQ3568A | *-* | | conserved hypothetical protein | |
| HQ3570A | *-* | | conserved hypothetical protein | |
| HQ3571A | *-* | | conserved hypothetical protein | |
| HQ3572A | *-* | | conserved hypothetical protein | |
| HQ3573A | *-* | | conserved hypothetical protein | |
| HQ3575A | *-* | | conserved hypothetical protein | |
| HQ3577A | *-* | | conserved hypothetical protein | |
| HQ3578A | *-* | | conserved hypothetical protein | |
| HQ3579D | *-* | | conserved hypothetical protein | |
| HQ3580A | *-* | | conserved hypothetical protein | |
| HQ3581A | *-* | | conserved hypothetical protein | |
| HQ3583A | *-* | | conserved hypothetical protein | |
| HQ3584A | *-* | | conserved hypothetical protein | |
| HQ3585A | *-* | | conserved hypothetical protein | |
| HQ3587A | *-* | | conserved hypothetical protein | |
| HQ3592A | *-* | | conserved hypothetical protein | |
| HQ3593A | *-* | | conserved hypothetical protein | |
| HQ3594A | *-* | | conserved hypothetical protein | |
| HQ3595A | *-* | | conserved hypothetical protein | |
| HQ3597A | *-* | | conserved hypothetical protein | |
| HQ3598A | *-* | | conserved hypothetical protein | |
| HQ3600A | *-* | | chitinase TP rich domain homolog | |
| HQ3603A | *-* | | conserved hypothetical protein | |
| HQ3604A | *-* | | conserved hypothetical protein | |
| HQ3605A | *-* | | conserved hypothetical protein | |
| HQ3606A | *-* | | conserved hypothetical protein | |
| HQ3610A | *-* | | conserved hypothetical protein | |
| HQ3633A | *-* | | conserved hypothetical protein | |
| HQ3637A | *-* | | conserved hypothetical protein | |
| HQ3639A | *-* | | conserved hypothetical protein | |
| HQ3644A | *-* | | membrane-associated phosphatase homolog, PAP2 family | |
| HQ3645A | *-* | | conserved hypothetical protein | |
| HQ3647A | *-* | | conserved hypothetical protein | |
| HQ3649A | *-* | | conserved hypothetical protein | |
| HQ3653A | *-* | | conserved hypothetical protein | |
| HQ3657A | *-* | | conserved hypothetical protein | |
| HQ3658A | *-* | | conserved hypothetical protein | |
| HQ3659A | *-* | | conserved hypothetical protein | |
| HQ3660A | *-* | | conserved hypothetical protein | |
| HQ3663A | *-* | | conserved hypothetical protein | |
| HQ3677A | *-* | | conserved hypothetical protein | |
| HQ3678A | *-* | | conserved hypothetical protein | |
| HQ3691A | *-* | | conserved hypothetical protein | |
| HQ3693A | *-* | | conserved hypothetical protein | |
| HQ3705A | *-* | | CBS domain protein | |
| HQ3722A | *-* | | conserved hypothetical protein | |
| HQ3724A | *-* | | conserved hypothetical protein | |
| HQ3727A | *-* | | conserved hypothetical protein | |
| HQ3728A | *-* | | conserved flagella cluster protein (probable integral membrane protein) | |
| HQ3734A | *-* | | conserved hypothetical protein | |
| HQ3735A | *-* | | conserved hypothetical protein | |
| HQ3736A | *-* | | conserved hypothetical protein | |
| HQ3737A | *-* | | conserved hypothetical protein | |
| HQ4003A | *-* | | conserved hypothetical protein | |
| HQ4004A | *-* | | conserved hypothetical protein | |
| HQ4009A | *-* | | conserved hypothetical protein | |
| HQ4010A | *-* | | conserved hypothetical protein | |
| HQ4012A | *-* | | conserved hypothetical protein | |
| HQ4016A | *-* | | conserved hypothetical protein | |
| HQ4030A | *-* | | conserved hypothetical protein | |
| HQ4035A | *-* | | conserved hypothetical protein | |
| **HY** |  | | **hypothetical protein** | |
| *code* | *gene* | | *protein name* | |
| HQ1032A | *-* | | Hypothetical protein | |
| HQ1072A | *-* | | hypothetical protein | |
| HQ1092A | *-* | | hypothetical protein | |
| HQ1096A | *-* | | hypothetical protein | |
| HQ1111A | *-* | | hypothetical protein | |
| HQ1114A | *-* | | hypothetical protein | |
| HQ1122A | *-* | | hypothetical protein | |
| HQ1131A | *-* | | hypothetical protein | |
| HQ1133A | *-* | | hypothetical protein | |
| HQ1139A | *-* | | hypothetical protein | |
| HQ1142A | *-* | | hypothetical protein | |
| HQ1148A | *-* | | hypothetical protein | |
| HQ1150A | *-* | | hypothetical protein | |
| HQ1191A | *-* | | hypothetical protein | |
| HQ1199A | *-* | | hypothetical protein | |
| HQ1202A | *-* | | hypothetical protein | |
| HQ1204A | *-* | | hypothetical protein | |
| HQ1210A | *-* | | hypothetical protein | |
| HQ1211A | *-* | | hypothetical protein | |
| HQ1258A | *-* | | hypothetical protein | |
| HQ1293A | *-* | | hypothetical protein | |
| HQ1297A | *-* | | hypothetical protein | |
| HQ1311A | *-* | | hypothetical protein | |
| HQ1320A | *-* | | hypothetical protein | |
| HQ1326A | *-* | | hypothetical protein | |
| HQ1327A | *-* | | hypothetical protein | |
| HQ1347A | *-* | | hypothetical protein | |
| HQ1351A | *-* | | hypothetical protein | |
| HQ1362A | *-* | | hypothetical protein | |
| HQ1400A | *-* | | hypothetical protein | |
| HQ1401A | *-* | | hypothetical protein | |
| HQ1428A | *-* | | hypothetical protein | |
| HQ1440A | *-* | | hypothetical protein | |
| HQ1441A | *-* | | hypothetical protein | |
| HQ1446A | *-* | | hypothetical protein | |
| HQ1447A | *-* | | hypothetical protein | |
| HQ1458A | *-* | | hypothetical protein | |
| HQ1459A | *-* | | hypothetical protein | |
| HQ1463A | *-* | | hypothetical protein | |
| HQ1498A | *-* | | hypothetical protein | |
| HQ1501A | *-* | | hypothetical protein | |
| HQ1502A | *-* | | hypothetical protein | |
| HQ1571A | *-* | | hypothetical protein | |
| HQ1574A | *-* | | hypothetical protein | |
| HQ1593A | *-* | | hypothetical protein | |
| HQ1600A | *-* | | hypothetical protein | |
| HQ1694A | *-* | | hypothetical protein | |
| HQ1757A | *-* | | hypothetical protein | |
| HQ1766A | *-* | | hypothetical protein | |
| HQ1817A | *-* | | hypothetical protein | |
| HQ1821A | *-* | | hypothetical protein | |
| HQ1824A | *-* | | hypothetical protein | |
| HQ1826A | *-* | | hypothetical protein | |
| HQ1827A | *-* | | hypothetical protein | |
| HQ1848A | *-* | | hypothetical protein | |
| HQ1849A | *-* | | hypothetical protein | |
| HQ1865A | *-* | | hypothetical protein | |
| HQ1893A | *-* | | hypothetical protein | |
| HQ1895A | *-* | | hypothetical protein | |
| HQ1906A | *-* | | hypothetical protein | |
| HQ1913A | *-* | | hypothetical protein | |
| HQ1915A | *-* | | hypothetical protein | |
| HQ1917A | *-* | | hypothetical protein | |
| HQ1918A | *-* | | hypothetical protein | |
| HQ1923A | *-* | | hypothetical protein | |
| HQ1925A | *-* | | hypothetical protein | |
| HQ1935A | *-* | | hypothetical protein | |
| HQ1962A | *-* | | hypothetical protein | |
| HQ1963A | *-* | | hypothetical protein | |
| HQ1966A | *-* | | hypothetical protein | |
| HQ1967A | *-* | | hypothetical protein | |
| HQ1971A | *-* | | hypothetical protein | |
| HQ1995A | *-* | | hypothetical protein | |
| HQ1997A | *-* | | hypothetical protein | |
| HQ2003A | *-* | | hypothetical protein | |
| HQ2008A | *-* | | hypothetical protein | |
| HQ2012A | *-* | | hypothetical protein | |
| HQ2013A | *-* | | hypothetical protein | |
| HQ2016A | *-* | | hypothetical protein | |
| HQ2037A | *-* | | hypothetical protein | |
| HQ2042A | *-* | | hypothetical protein | |
| HQ2044A | *-* | | hypothetical protein | |
| HQ2058A | *-* | | hypothetical protein | |
| HQ2060A | *-* | | hypothetical protein | |
| HQ2063A | *-* | | hypothetical protein | |
| HQ2065A | *-* | | hypothetical protein | |
| HQ2067A | *-* | | hypothetical protein | |
| HQ2069A | *-* | | hypothetical protein | |
| HQ2070A | *-* | | hypothetical protein | |
| HQ2071A | *-* | | hypothetical protein | |
| HQ2073A | *-* | | hypothetical protein | |
| HQ2074A | *-* | | hypothetical protein | |
| HQ2076A | *-* | | hypothetical protein | |
| HQ2081A | *-* | | hypothetical protein | |
| HQ2082A | *-* | | hypothetical protein | |
| HQ2088A | *-* | | hypothetical protein | |
| HQ2089A | *-* | | hypothetical protein | |
| HQ2091A | *-* | | hypothetical protein | |
| HQ2094A | *-* | | hypothetical protein | |
| HQ2095A | *-* | | hypothetical protein | |
| HQ2099A | *-* | | hypothetical protein | |
| HQ2102A | *-* | | hypothetical protein | |
| HQ2103A | *-* | | hypothetical protein | |
| HQ2104A | *-* | | hypothetical protein | |
| HQ2107A | *-* | | hypothetical protein | |
| HQ2108A | *-* | | hypothetical protein | |
| HQ2110A | *-* | | hypothetical protein | |
| HQ2111A | *-* | | hypothetical protein | |
| HQ2112A | *-* | | hypothetical protein | |
| HQ2116A | *-* | | hypothetical protein | |
| HQ2122A | *-* | | hypothetical protein | |
| HQ2125A | *-* | | hypothetical protein | |
| HQ2126A | *-* | | hypothetical protein | |
| HQ2142A | *-* | | hypothetical protein | |
| HQ2143A | *-* | | hypothetical protein | |
| HQ2147A | *-* | | hypothetical protein | |
| HQ2151A | *-* | | hypothetical protein | |
| HQ2179A | *-* | | hypothetical protein | |
| HQ2181A | *-* | | hypothetical protein | |
| HQ2182A | *-* | | hypothetical protein | |
| HQ2187A | *-* | | hypothetical protein | |
| HQ2188A | *-* | | hypothetical protein | |
| HQ2199A | *-* | | hypothetical protein | |
| HQ2203A | *-* | | hypothetical protein | |
| HQ2216A | *-* | | hypothetical protein | |
| HQ2219A | *-* | | hypothetical protein | |
| HQ2220A | *-* | | hypothetical protein | |
| HQ2227A | *-* | | hypothetical protein | |
| HQ2229A | *-* | | hypothetical protein | |
| HQ2239A | *-* | | hypothetical protein | |
| HQ2250A | *-* | | hypothetical protein | |
| HQ2251A | *-* | | hypothetical protein | |
| HQ2261A | *-* | | hypothetical protein | |
| HQ2264A | *-* | | hypothetical protein | |
| HQ2268A | *-* | | hypothetical protein | |
| HQ2272A | *-* | | hypothetical protein | |
| HQ2274A | *-* | | hypothetical protein | |
| HQ2275A | *-* | | hypothetical protein | |
| HQ2277A | *-* | | hypothetical protein | |
| HQ2278A | *-* | | hypothetical protein | |
| HQ2284A | *-* | | hypothetical protein | |
| HQ2286A | *-* | | hypothetical protein | |
| HQ2290A | *-* | | hypothetical protein | |
| HQ2293A | *-* | | hypothetical protein | |
| HQ2318A | *-* | | hypothetical protein | |
| HQ2333A | *-* | | hypothetical protein | |
| HQ2337A | *-* | | hypothetical protein | |
| HQ2348A | *-* | | hypothetical protein | |
| HQ2377A | *-* | | hypothetical protein | |
| HQ2383A | *-* | | hypothetical protein | |
| HQ2386A | *-* | | hypothetical protein | |
| HQ2387A | *-* | | hypothetical protein | |
| HQ2443A | *-* | | hypothetical protein | |
| HQ2444A | *-* | | hypothetical protein | |
| HQ2449A | *-* | | hypothetical protein | |
| HQ2459A | *-* | | hypothetical protein | |
| HQ2480A | *-* | | hypothetical protein | |
| HQ2484A | *-* | | hypothetical protein | |
| HQ2486A | *-* | | hypothetical protein | |
| HQ2513A | *-* | | hypothetical protein | |
| HQ2514A | *-* | | hypothetical protein | |
| HQ2516A | *-* | | hypothetical protein | |
| HQ2518A | *-* | | hypothetical protein | |
| HQ2525A | *-* | | hypothetical protein | |
| HQ2526A | *-* | | hypothetical protein | |
| HQ2532A | *-* | | hypothetical protein | |
| HQ2534A | *-* | | hypothetical protein | |
| HQ2553A | *-* | | hypothetical protein (probable membrane protein) | |
| HQ2554A | *-* | | hypothetical protein | |
| HQ2558A | *-* | | hypothetical protein | |
| HQ2572A | *-* | | hypothetical protein | |
| HQ2633A | *-* | | hypothetical protein | |
| HQ2645A | *-* | | hypothetical protein | |
| HQ2662A | *-* | | hypothetical protein | |
| HQ2666A | *-* | | hypothetical protein | |
| HQ2701A | *-* | | hypothetical protein | |
| HQ2742A | *-* | | hypothetical protein | |
| HQ2745A | *-* | | hypothetical protein | |
| HQ2761A | *-* | | hypothetical protein | |
| HQ2784A | *-* | | hypothetical protein | |
| HQ2802A | *-* | | hypothetical protein | |
| HQ2803A | *-* | | hypothetical protein | |
| HQ2810A | *-* | | hypothetical protein | |
| HQ2811A | *-* | | hypothetical protein | |
| HQ2812A | *-* | | hypothetical protein | |
| HQ2815A | *-* | | hypothetical protein | |
| HQ2816A | *-* | | hypothetical protein | |
| HQ2817A | *-* | | hypothetical protein | |
| HQ2818A | *-* | | hypothetical protein | |
| HQ2848A | *-* | | hypothetical protein | |
| HQ2851A | *-* | | hypothetical protein | |
| HQ2875A | *-* | | hypothetical protein | |
| HQ2876A | *-* | | hypothetical protein | |
| HQ2897A | *-* | | hypothetical protein | |
| HQ2904A | *-* | | hypothetical protein | |
| HQ2911A | *-* | | hypothetical protein | |
| HQ2913A | *-* | | hypothetical protein | |
| HQ2914A | *-* | | hypothetical protein | |
| HQ2924A | *-* | | hypothetical protein | |
| HQ2946A | *-* | | hypothetical protein | |
| HQ2957A | *-* | | hypothetical protein | |
| HQ2960A | *-* | | hypothetical protein | |
| HQ2968A | *-* | | hypothetical protein | |
| HQ2976A | *-* | | hypothetical protein | |
| HQ2977A | *-* | | hypothetical protein | |
| HQ2980A | *-* | | hypothetical protein | |
| HQ2983A | *-* | | hypothetical protein | |
| HQ2988A | *-* | | hypothetical protein | |
| HQ2991A | *-* | | hypothetical protein | |
| HQ3008A | *-* | | hypothetical protein | |
| HQ3019A | *-* | | hypothetical protein | |
| HQ3037A | *-* | | hypothetical protein | |
| HQ3038A | *-* | | hypothetical protein | |
| HQ3041A | *-* | | hypothetical protein | |
| HQ3082A | *-* | | hypothetical protein | |
| HQ3103A | *-* | | hypothetical protein | |
| HQ3155A | *-* | | hypothetical protein | |
| HQ3156A | *-* | | hypothetical protein | |
| HQ3210A | *-* | | hypothetical protein | |
| HQ3223A | *-* | | hypothetical protein | |
| HQ3225A | *-* | | hypothetical protein | |
| HQ3258A | *-* | | hypothetical protein | |
| HQ3273A | *-* | | hypothetical protein | |
| HQ3278A | *-* | | hypothetical protein | |
| HQ3279A | *-* | | hypothetical protein | |
| HQ3280A | *-* | | hypothetical protein | |
| HQ3281A | *-* | | hypothetical protein | |
| HQ3282A | *-* | | hypothetical protein | |
| HQ3285A | *-* | | hypothetical protein | |
| HQ3286A | *-* | | hypothetical protein | |
| HQ3287A | *-* | | hypothetical protein | |
| HQ3290A | *-* | | hypothetical protein | |
| HQ3292A | *-* | | hypothetical protein | |
| HQ3294A | *-* | | hypothetical protein | |
| HQ3296A | *-* | | hypothetical protein | |
| HQ3314A | *-* | | hypothetical protein | |
| HQ3329A | *-* | | hypothetical protein | |
| HQ3377A | *-* | | hypothetical protein | |
| HQ3401A | *-* | | hypothetical protein | |
| HQ3409A | *-* | | hypothetical protein | |
| HQ3446A | *-* | | hypothetical protein | |
| HQ3453A | *-* | | hypothetical protein | |
| HQ3462A | *-* | | hypothetical protein | |
| HQ3481A | *-* | | hypothetical protein | |
| HQ3487A | *-* | | hypothetical protein | |
| HQ3488A | *-* | | hypothetical protein | |
| HQ3495A | *-* | | hypothetical protein | |
| HQ3501A | *-* | | hypothetical protein | |
| HQ3502A | *-* | | hypothetical protein | |
| HQ3514A | *-* | | hypothetical protein | |
| HQ3531A | *-* | | hypothetical protein | |
| HQ3534A | *-* | | hypothetical protein | |
| HQ3536A | *-* | | hypothetical protein | |
| HQ3541A | *-* | | hypothetical protein | |
| HQ3546A | *-* | | hypothetical protein | |
| HQ3576A | *-* | | hypothetical protein | |
| HQ3582A | *-* | | hypothetical protein | |
| HQ3589A | *-* | | hypothetical protein | |
| HQ3591A | *-* | | hypothetical protein | |
| HQ3596A | *-* | | hypothetical protein | |
| HQ3599A | *-* | | hypothetical protein | |
| HQ3674A | *-* | | hypothetical protein | |
| HQ3687A | *-* | | hypothetical protein | |
| HQ3703A | *-* | | hypothetical protein | |
| HQ3726A | *-* | | hypothetical protein | |
| HQ3732A | *-* | | hypothetical protein | |
| HQ4002A | *-* | | hypothetical protein | |
| HQ4005A | *-* | | hypothetical protein | |
| HQ4006A | *-* | | hypothetical protein | |
| HQ4008A | *-* | | hypothetical protein | |
| HQ4011A | *-* | | hypothetical protein | |
| HQ4013A | *-* | | hypothetical protein | |
| HQ4017A | *-* | | hypothetical protein | |
| HQ4018A | *-* | | hypothetical protein | |
| HQ4021A | *-* | | hypothetical protein | |
| HQ4024A | *-* | | hypothetical protein | |
| HQ4025A | *-* | | hypothetical protein | |
| HQ4027A | *-* | | hypothetical protein | |
| HQ4028A | *-* | | hypothetical protein | |
| HQ4029A | *-* | | hypothetical protein | |
| HQ4031A | *-* | | hypothetical protein | |
| HQ4034A | *-* | | hypothetical protein | |
| HQ4036A | *-* | | hypothetical protein | |
| HQ4039A | *-* | | hypothetical protein | |
